# Functional landscape of mechanistic diversity in 27 claudin family members at tight junctions

**DOI:** 10.1126/sciadv.adx7431

**Published:** 2025-10-31

**Authors:** Hiroka Kashihara, Hiroo Tanaka, Manabu Kitamata, Gen Shiratsuchi, Tatsuya Katsuno, Kazuto Tsukita, Tomoki Nishida, Mayumi Hamasaki, Fabian Eisenstein, Hiroshi Suzuki, Shun Nakamura, Koji Aoyama, Takeshi Yagi, Radostin Danev, Yoshinori Fujiyoshi, Atsushi Tamura, Sachiko Tsukita

**Affiliations:** ^1^ Advanced Comprehensive Research Organization, Teikyo University, Itabashi-ku, Japan.; ^2^ Department of Pharmacology, Teikyo University School of Medicine, Itabashi-ku, Japan.; ^3^ Graduate School of Frontier Biosciences, Osaka University, Suita, Japan.; ^4^ Center for Anatomical Studies, Graduate School of Medicine, Kyoto University, Sakyo-ku, Japan.; ^5^ Department of Neurology, Graduate School of Medicine, Kyoto University, Sakyo-ku, Japan.; ^6^ Japan Textile Products Quality and Technology Center, Chuo-ku, Japan.; ^7^ Graduate School of Medicine, The University of Tokyo, Bunkyo-ku, Japan.; ^8^ Advanced Research Initiative, Institute of Integrated Research, Institute of Science Tokyo, Bunkyo-ku, Japan.

## Abstract

The diversity of epithelial paracellular barriers, essential for various biological functions, is primarily determined by the combination of 27 claudins (Cldns) that form tight junctions (TJs). However, the basis of their functional diversity remains largely unexplored. Here, we generated complete *Cldn*-null mouse epithelial cells to reconstitute the TJ paracellular barrier (TJ barrier) with individual Cldns. Each Cldn establishes its respective TJ barrier, either autonomously or nonautonomously, exhibiting distinct ion conductivity and selectivity. Clustering algorithms revealed a previously unidentified classification of Cldns into four main classes with well-defined subclasses, moving beyond the conventional paracellular barrier–versus–channel-forming dichotomy. Our findings, including the in vivo Cldn dynamics, provide a framework for understanding TJ barrier diversity and plasticity, offering insights into organ-specific homeostasis and guiding therapeutic strategies targeting TJ barriers in health and disease.

## Teaser

Unraveling the functions of 27 claudins uncovers a framework for tight junction paracellular barrier diversity and plasticity.

## INTRODUCTION

Vertebrates are organized by distinct fluid compartments, delineated by epithelial, endothelial, and mesothelial cell sheets, which serve as platforms for biological systems (*1*–*3*). Typically, epithelial cell sheets contain tight junctions (TJs) located at the most apical region of cell-cell junctions (TJ region). TJs minimize intercellular space to form the continuous TJ paracellular barrier (TJ barrier) (*1*–*8*). TJ barriers are fundamental to the function of biological systems in vivo such as the blood-brain barrier, skin barrier, and blood-testis barrier, among others. Deficiencies in TJ barriers are linked to diseases, including cancer, inflammation, metabolic disorders, and neurological disorders (*1*–*14*). In TJs, claudins (Cldns), four–transmembrane-spanning cell-cell adhesion molecules of 20 to 30 kDa, polymerize in antiparallel double rows to form TJ strands (*15*–*18*). These strands interact with those from adjacent cells and are essential for the structural and functional integrity of TJ barriers.

The *Cldn* family comprises 27 members in mice and humans (with Cldn13 absent in humans) (*19*), expressed in diverse combinations and quantities across organs and cell lines, thereby basically establishing organ- and cell-specific TJ barriers (*9*–*12*, *20*–*24*). Some Cldns create paracellular channel functions within TJ barriers (*25*–*30*), enabling selective ion and water transport in organs such as the kidney, intestine, and liver (*31*–*35*). The functional diversity among 27 Cldns is thought to shape the diversity of TJ barriers across various biological and pathological systems (*1*–*8*). However, our understanding of intrinsic properties of individual Cldns at TJs remains very limited because nearly all studies have relied on overexpression or suppression of Cldns in TJ barriers, which include multiple endogenous Cldns. To date, Cldns have been functionally categorized primarily as forming either paracellular barriers or channels in preexisting TJ barriers (*1*–*8*).

To explore intrinsic properties of individual Cldns for TJ barrier diversity, we generated the first complete *Cldn*-null epithelial cell line, entirely devoid of all 27 *Cldn* family members, unlike previously reported knockout (KO) cells that retained some Cldns (*36*). We then expressed each of the 27 mouse *Cldn* family members in the *Cldn*-null cells, establishing 27 kinds of single *Cldn*-expressing cells. On the basis of our morphological and functional analyses of those cells, clustering algorithms led us to a classification of Cldns into four main classes, with well-defined subclasses. Furthermore, in vivo studies revealed the spatiotemporal dynamics of Cldns in response to homeostatic cues, at least indirectly related to certain Cldns’ dynamic behavior in cancer and other diseases, suggesting promising therapeutic targets (*37*–*40*). Our single Cldn analyses provide an indispensable framework for understanding and manipulating TJ barriers composed of multiple Cldns in vivo, highlighting their intricate diversity across biological and pathological systems, with implications for health promotion and disease treatment.

## RESULTS

### First complete *Cldn*-null epithelial cells

**Fig. 1.**
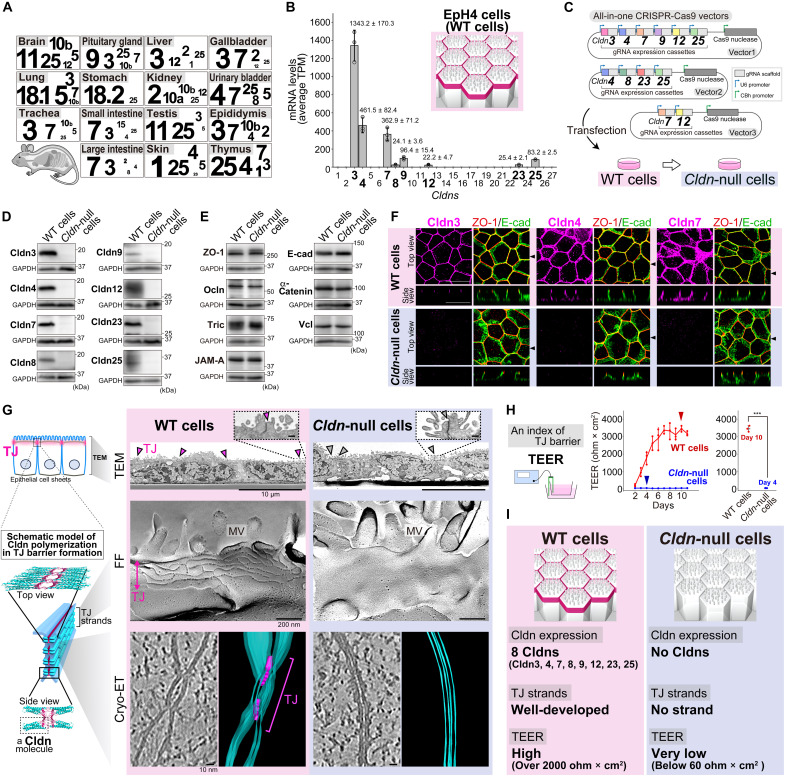
Generation of *Cldn*-null epithelial cells. (**A**) Quantitative PCR (qPCR) of *Cldns* in mouse organs, normalized to *Gapdh*. Font size indicates relative expression. (**B**) RNA-seq of *Cldns* in EpH4 cells (WT cells), shown as transcripts per million (TPM; *n* = 3 libraries). The same data were used in fig. S2C. Values are means ± SDs. (**C**) Generation of *Cldn*-null cells via CRISPR-Cas9 targeting *Cldn3*, *4*, *7*, *8*, *9*, *12*, *23*, and *25*. (**D** and **E**) Immunoblotting of Cldns (D), and TJ proteins [zonula occludens-1 (ZO-1), occludin (Ocln), tricellulin (Tric), and junctional adhesion molecule-A (JAM-A)] and adherens junction proteins [E-cadherin (E-cad), α-catenin, and vinculin (Vcl)] (E) in WT and *Cldn*-null cells. GAPDH (glyceraldehyde-3-phosphate dehydrogenase): loading control. (**F**) Super-resolution immunofluorescence (IF) microscopy of WT and *Cldn*-null cells for Cldn3, 4, or 7 with ZO-1 and E-cad. Z-stacks (top) and orthogonal views at arrowheads (bottom). Scale bars, 10 μm. (**G**) Thin-section electron microscopy (TEM), freeze-fracture electron microscopy (FF), and cryo–electron tomography (cryo-ET) of WT and *Cldn*-null cells. Arrowheads: TJs. Cryo-ET shows tomographic slices and three-dimensional (3D) reconstructions from the most apical region of lateral membranes (false-colored images), highlighting intercellular regions of TJs (magenta) and lipid bilayers (cyan). The same data of cryo-ET were used in fig. S3A. For TEM and FF, at least 10 profiles were analyzed, whereas for cryo-ET, a minimum of 5 profiles were examined. MV, microvilli. Scale bars, 200 nm (inset). (**H**) Transepithelial electrical resistance (TEER) of WT and *Cldn*-null cells (*n* = 3 wells). Time courses of the TEER values from day 2 to 11 (left) and the peak TEER values (right) are shown. Arrowheads indicate the individual peaks. The same data were used in fig. S2E, where statistical significance was tested together. Values are means ± SDs. ***P < 0.001 [Welch’s one-way analysis of variance (ANOVA) with Games-Howell test]. (**I**) Characteristics of WT and *Cldn*-null cells.

Twenty-seven *Cldn* family members exhibited varied expression profiles across mouse organs and cell lines (Fig. 1, A and B, and fig. S1A), which are key determinants of TJ barrier diversity (*1*–*8*). To investigate the mechanisms underlying TJ barrier diversity by the 27 Cldns, we first generated a complete *Cldn*-null cell line using mouse mammary gland–derived epithelial EpH4 cells [wild-type (WT) cells], allowing subsequent expression of each of the 27 *Cldns* in the *Cldn*-null cells to establish single *Cldn*-expressing cells for all 27 *Cldns*. WT cells endogenously express eight *Cldns* (*Cldn3*, *4*, *7*, *8*, *9*, *12*, *23*, and *25*), forming strong TJ barriers in confluent epithelial cell sheets (Fig. 1B). A multiplexed CRISPR-Cas9 system enabled the complete KO of *Cldn3, 4, 7, 8, 9, 12, 23,* and *25* without compensatory expression of other *Cldns* in *Cldn*-null cells, as confirmed by sequencing and immunoblotting (Fig. 1, C and D, and figs. S1, B to D, and S2C). RNA sequencing (RNA-seq) and immunoblotting revealed almost no notable changes in mRNA or protein expression of other cell adhesion–related genes (Fig. 1E and fig. S2D).

A monolayer configuration of WT and *Cldn*-null cells in confluent cultures was revealed by super-resolution immunofluorescence (IF) and conventional thin-section electron microscopy (TEM) (Fig. 1, F and G). IF showed that Cldn3, 4, 7, 8, 9, 12, 23, and 25 colocalized with TJ scaffold protein zonula occludens-1 (ZO-1) at TJs in WT cells but not in *Cldn*-null cells, where ZO-1 and other cell adhesion proteins remained localized (Fig. 1F and fig. S2, A and B). Freeze-fracture electron microscopy (FF) revealed a complete loss of TJ strands in *Cldn*-null cells, contrasting with well-formed TJ strands in WT cells (Fig. 1G). For these morphological analyses, more than 10 independent cell profiles were systematically examined. Cryo–electron tomography (Cryo-ET) of rapidly frozen cells, without chemical fixation, showed TJs as “kissing points,” very close cell-cell contacts forming a TJ barrier in WT cells, which were absent in *Cldn*-null cells (Fig. 1G, fig. S3A, and movies S1 and S2), based on the analysis of at least five representative profiles. However, conventional TEM of chemically fixed cells showed kissing-point–like structures in *Cldn*-null cells (fig. S3B), likely artifacts from fixation-induced membrane distortions (*41*).

Physiologically, transepithelial electrical resistance (TEER) measurements revealed a loss of TJ barrier function in *Cldn*-null cells (below 60 ohms × cm^2^) contrasted to high TJ barrier function in WT cells (more than 2000 ohms × cm^2^) under confluent conditions (Fig. 1H). These results are consistent with findings from *ZO-1*/*ZO-2* double KO cells (*42*), which exhibited almost no TJ barriers due to the lack of Cldn polymerization (fig. S2E). Collectively, *Cldn*-null cells lack TJs morphologically and functionally (Fig. 1I), providing an excellent foundation for establishing single *Cldn*-expressing epithelial cells, a promising system for the comparative functional analysis of individual Cldns.

### TJ barriers reconstituted by Cldn2 or Cldn3 as typical examples

**Fig. 2.**
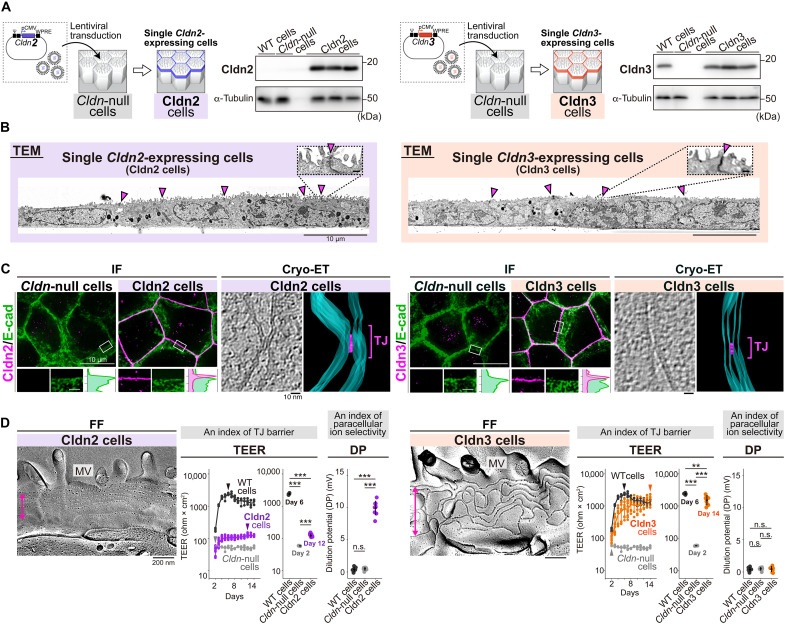
TJ reconstitution by exogenous *Cldn2* or *Cldn3* expression in *Cldn*-null cells. (**A**) Generation of single *Cldn2*- or *Cldn3*-expressing cells (Cldn2 cells or Cldn3 cells) via lentivirus-mediated gene transduction into *Cldn*-null cells. Immunoblotting confirmed Cldn2 or Cldn3 expressions in Cldn2 or Cldn3 cells (three clones, respectively). α-Tubulin: loading control. (**B**) TEM of Cldn2 and Cldn3 cells. Arrowheads: TJs. Scale bars, 10 μm and 200 nm (insets). (**C**) IF and cryo-ET of Cldn2 and Cldn3 cells. IF shows costaining of Cldn2 or Cldn3 with E-cad, with magnified views and intensity plots (bottom panels). Scale bars, 10 μm and 1 μm (insets). Cryo-ET shows tomographic slices and 3D reconstructions from the most apical region of the lateral membranes (false-colored images), highlighting the intercellular regions of TJs (magenta) and lipid bilayers (cyan). The same data of cryo-ET were used in fig. S3A. Scale bars, 10 nm. (**D**) FF, TEER, and dilution potential (DP) of Cldn2 and Cldn3 cells for morphological and functional evaluation of TJ barriers. TEER values were measured from day 2 to 14 (*n* = 4, 4, 12, and 9 wells for WT, *Cldn*-null, Cldn2, and Cldn3 cells, respectively), and DP values were measured on day 14 (*n* = 9 wells, respectively). The peak values of TEER are also shown. Arrowheads indicate the individual peaks. Values are means ± SDs. **P < 0.01, ***P < 0.001, and n.s.: not significant (Welch’s one-way ANOVA with Games-Howell test). Scale bars, 200 nm.

To assess their suitability for single *Cldn* expression, we generated stable cell lines expressing *Cldn2* or *Cldn3* in *Cldn*-null cells (Cldn2 or Cldn3 cells) (Fig. 2A and fig. S3C). In cells with preexisting TJs formed by multiple Cldns, Cldn2 and Cldn3 are generally recognized as forming typical TJ paracellular channels or TJ barriers, respectively (*21*, *24*, *25*, *27*, *28*, *33*–*35*). Cldn2 and Cldn3 cells exhibited a monolayer configuration at full confluence (Fig. 2, B and C), with each Cldn colocalizing with ZO-1 at TJs (fig. S6). Cryo-ET revealed TJ kissing points in both cell lines (Fig. 2C, fig. S3A, and movies S3 and S4), consistent with well-formed TJ strands observed in FF (Fig. 2D).

TEER measurement showed that Cldn3 cells exhibited high TEER (~1500 ohms × cm^2^), indicating a strong TJ barrier, while Cldn2 cells showed intermediate TEER (~120 ohms × cm^2^), still higher than *Cldn*-null cells (below 60 ohms × cm^2^), also suggesting a functional TJ barrier. Within the TJ barrier, Cldn2 cells formed cation-selective paracellular channels, as shown by the positive transepithelial dilution potential (DP) in Ussing chamber experiments (Fig. 2D). Furthermore, the addition of lanthanum chloride (LaCl_3_) to Cldn2 cells, which has been reported to inhibit the paracellular channel activity mediated by Cldn2 (*43*), resulted in a marked increase in TEER (fig. S3D), highlighting the TJ barrier function and the paracellular channel function of Cldn2. Although the formation of paracellular channels or TJ barriers by Cldn2 or Cldn3 has been reported in multiple Cldns backgrounds (*21*, *24*, *25*, *27*, *28*, *33*–*35*), our cell lines provided evidence that they autonomously form TJ barriers in the absence of other members, either with or without paracellular channel function. Thus, we confirmed the suitability of *Cldn*-null cells for single *Cldn* expression to elucidate the intrinsic properties of individual Cldns.

### TJ barriers reconstituted with each of 27 Cldns

**Fig. 3.**
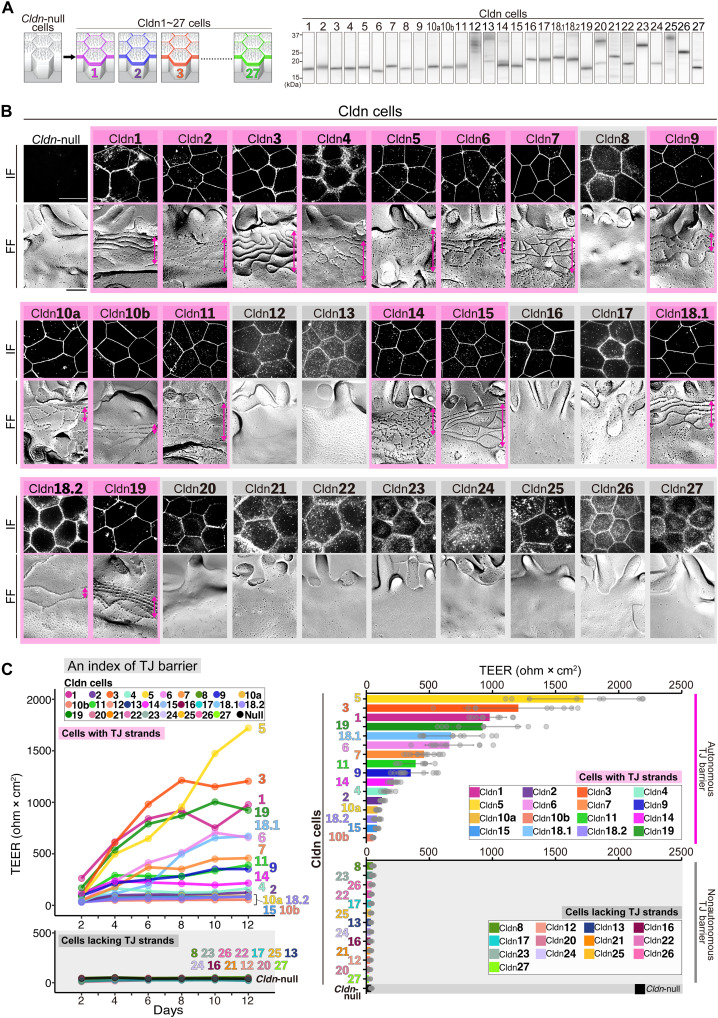
Generation of single *Cldn*-expressing epithelial cells for 27 *Cldn* family members. (**A**) Generation of single *Cldn*-expressing cells for 27 *Cldns* (Cldn1 to 27 cells). Immunoblotting for individual Cldns in their respective cells using specific anti-Cldn antibodies for Cldn1 to 12, 14, 15, 18.1, 18.2, and 19, along with anti-Flag antibody for the remaining Cldns. (**B**) IF and FF of Cldn1 to 27 cells. IF using specific anti-Cldn antibodies for Cldn1 to 11, 14, 15, 18.1, 18.2, and 19 and anti-Flag antibody for the remaining Cldns are shown. FF shows TJ strands (bidirectional arrows) in specific Cldn cells (pink), with no TJ strands in the remaining Cldn cells (gray). IF (Cldn3 staining) and FF of *Cldn*-null cells confirm the absence of TJs. Scale bars, 10 μm (IF) and 200 nm (FF). (**C**) TJ barriers of Cldn1 to 27 cells. Average TEER values of Cldn1 to 27 cells (*n* = 6 to 12 wells, respectively) and *Cldn*-null cells (*n* = 4 wells) measured from day 2 to 12 are shown in the left graph. TEER values at day 12 during the culture period are arranged in descending order and shown in the right graph. Values are means ± SDs.

We next generated a series of cell lines expressing each of all 27 *Cldn* family members individually (Cldn1 to 27 cells) (Fig. 3A and fig. S3C), including isoforms such as *Cldn10a/10b* and *Cldn18.1/18.2* (*22*, *26*, *44*), on the *Cldn*-null cell background. These lines were established using nontagged constructs of *Cldns*, screened with specific antibodies, except for *Cldn12*, *13*, *16*, *17*, and *20* to *27*, where Flag-tagged constructs were used because of the lack of antibodies. The Flag tag had almost no effect on TEER, unlike the enhanced green fluorescent protein (EGFP) tag (fig. S4A). Three cell clones were established for each Cldn (fig. S3C), except for Cldn13, 20, 25, and 27, for which two clones each were generated. Expression was confirmed by immunoblotting and IF, and almost no apparent differences were observed among clones (Fig. 3, A and B, and figs. S5 to S7).

IF and TEM showed that all 27 single *Cldn*-expressing cell lines formed continuous monolayers at full confluence, with each Cldn localized at the ZO-1–positive TJ region (Fig. 3B and figs. S6 and S7). FF revealed that TJ strands autonomously formed in 16 single *Cldn*-expressing cell lines (Cldn1 to 7, 9, 10a, 10b, 11, 14, 15, 18.1, 18.2, and 19 cells) but were absent in others (Cldn8, 12, 13, 16, 17, and 20 to 27 cells) (Fig. 3B and fig. S8). The latter showed diffuse IF localization of each Cldn in the ZO-1–positive TJ region (Fig. 3B and figs. S6 and S7). Given that none of the Flag-tagged Cldns formed TJ strands, we investigated whether the presence of the Flag tag interferes with strand formation. To directly assess this, we examined TJ strand formation by Cldn3 and Cldn2 with the Flag tag. We confirmed the previous conclusion that the Flag tag does not affect TJ strand formation (Fig. 3B and fig. S4B). In addition, we analyzed TJ strand formation by Cldn12, 16, 17, and 25 without the Flag tag, as we were able to obtain antibodies against these Cldns, albeit with some cross-reactivity. FF revealed that none of these Cldns formed TJ strands, consistent with the results obtained using their Flag-tagged counterparts (Flag-Cldn12, 16, 17, and 25) (Fig. 3B and fig. S4C).

TEER measurements showed that 16 *Cldn*-expressing cell lines with TJ strands, as revealed by FF, exhibited elevated TEER (~60 to ~1700 ohms × cm^2^), exceeding ~60 ohms × cm^2^ in *Cldn*-null cells (Cldn5, 3, 1, 19, 18.1, 6, 7, 11, 9, 14, 4, 2, 10a, 18.2, 15, and 10b cells, in descending order) (Fig. 3C). This variation suggests the autonomous formation of diverse TJ barriers with distinct paracellular channel functions. In contrast, cells lacking TJ strands (Cldn8, 12, 13, 16, 17, and 20 to 27 cells) showed low TEER (~60 ohms × cm^2^), similar to *Cldn*-null cells, indicating the absence of autonomous TJ barrier formation (Fig. 3C).

Collectively, our single *Cldn*-expressing cells revealed insights into the intrinsic properties of individual Cldns, morphologically and functionally. The broad TEER range in our cell lines, although masked under conventional conditions with multiple endogenous Cldns, highlights the need for a more refined classification beyond the conventional dichotomous classification of TJ barrier–versus–channel functions.

### Functional diversity of Cldns in TJ barriers

**Fig. 4.**
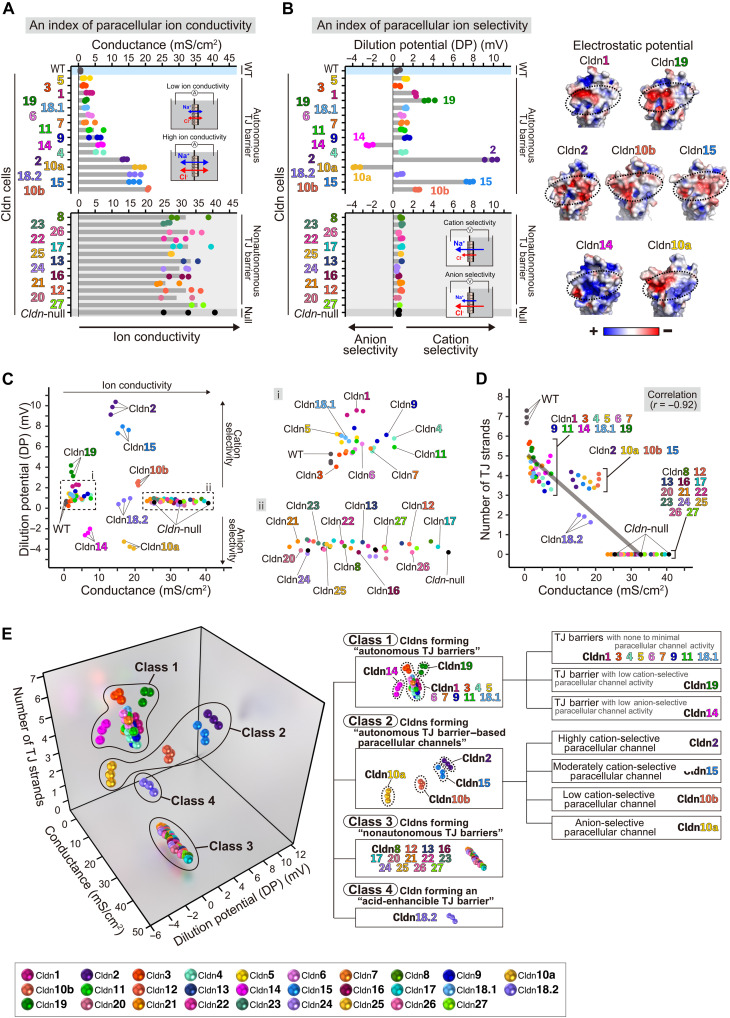
Functional diversity of 27 Cldn family members at TJs. (**A** and **B**) Transepithelial electrical conductance (A) and DP (B; left) of Cldn1 to 27 cells. The average conductance and DP values of Cldn1 to 27 cells, WT, and *Cldn*-null cells (*n* = 2 to 3 wells, respectively) are displayed in ascending order in (A) and shown in the same order in (B; left). Structural model–based electrostatic potential analysis (B; right) shows negatively charged residues (red) on the extracellular β sheet domains of Cldn1, 2, 10b, 15, and 19 and positively charged residues (blue) on those of Cldn10a and 14. (**C**) 2D plot of conductance and DP of Cldn1 to 27 cells, WT, and *Cldn*-null cells from the data shown in (A) and (B), with enlarged images of selected regions (i and ii). (**D**) 2D plot of conductance and number of TJ strands of Cldn1 to 27 cells, WT, and *Cldn*-null cells from data shown in (A) and fig. S8. *r*, correlation coefficient. (**E**) Classification of Cldn family members based on a 3D plot depicting conductance, DP, and number of TJ strands of Cldn1 to 27 cells, derived from data in (C) and (D).

We comprehensively examined the electrophysiological properties of 27 single *Cldn*-expressing cell sheets at full confluence, focusing on transepithelial electrical conductance and DP, indicators of paracellular ion conductivity and selectivity. Thus, TJ strand–forming cells (Cldn1 to 7, 9, 10a, 10b, 11, 14, 15, 18.1, 18.2, and 19 cells) exhibited low to high conductance (2 to 21 mS/cm^2^) compared to that of *Cldn*-null cells, which was even higher (33 mS/cm^2^) (Fig. 4A). The wide conductance range suggested diverse autonomous TJ barrier functions. Conversely, Cldn8, 12, 13, 16, 17, and 20 to 27 cells, lacking TJ strands, exhibited high conductance similar to *Cldn*-null cells, indicating no autonomous TJ barriers (Fig. 4A).

Notably, TJ strand–forming Cldn cells exhibited varying degrees of positive or negative DP, indicating TJ barriers with different levels of paracellular cation or anion selectivity, respectively (Fig. 4B). Cldn2, 10b, and 15 cells displayed high conductance and high levels of positive DP (Fig. 4, A and B), reflecting TJ barriers with distinct cation-selective conductivity (Cldn2 > 15 > 10b), which is commonly referred to as cation-selective paracellular channel activities, whereas Cldn10a cells exhibited high conductance with distinct negative DP (Fig. 4, A and B), reflecting TJ barriers associated with anion-selective paracellular channels. Thus, each of these Cldns autonomously formed TJ barriers with paracellular channels in the absence of other members.

Cldn19 autonomously formed strong TJ barriers with low conductance and positive DP, indicative of low cation-selective paracellular channel activity. Cldn14 autonomously formed strong TJ barriers with low conductance and negative DP, indicative of low anion-selective paracellular channel activity (Fig. 4, A and B). These findings challenge the conventional view that ion selectivity is specific to traditional channel-forming Cldns, such as Cldn2 and Cldn15 (*1*–*8*). These diverse ion-selective paracellular channel activities aligned with structural modeling, which revealed negatively or positively charged residues in extracellular domains of the respective Cldns (Fig. 4B).

Two-dimensional mapping of conductance and DP illustrated the functional diversity of members (Fig. 4C). In addition, mapping conductance against TJ strand counts—indicators of paracellular ion conductivity and its structural basis—revealed a strong correlation (Fig. 4D), further highlighting their diversity.

### Classification of Cldns

Through comprehensive analyses of Cldn family members and their functional diversity in TJ barriers, we classified the 27 Cldn family members using an unbiased two-step DBSCAN (Density-Based Spatial Clustering of Applications with Noise) algorithm. First, we analyzed electrical conductance and the number of TJ strands, indicators of paracellular ion conductivity and its morphological basis (Fig. 4D and fig. S9A). Next, we integrated DPs, which serve as indices of ion selectivity (Fig. 4C and fig. S9A). This approach classified the members into four main classes (classes 1 to 4), with additional subclasses (Fig. 4E, fig. S9, and movie S5).

#### Class 1: Cldns forming “autonomous TJ barriers”

Class 1 comprises Cldn1, 3, 4, 5, 6, 7, 9, 11, 14, 18.1, and 19. These Cldns autonomously create TJ barriers with a high number of TJ strands, low conductance, and very low DP. Cldn1, 3, 4, 5, 6, 7, 9, 11, and 18.1 are subclassified as TJ barriers with none to minimal paracellular channel activity. In contrast, Cldn19 forms a low-conductance barrier with a moderately positive DP, indicating low paracellular channel activity. Thus, it is subclassified as a TJ barrier with a low cation-selective paracellular channel activity. Cldn14, which forms a low-conductance barrier with a moderately negative DP, is subclassified as a TJ barrier with a low anion-selective paracellular channel activity.

#### Class 2: Cldns forming “autonomous TJ barrier–based paracellular channels”

Class 2 comprises Cldn2, 10a, 10b, and 15, which form TJ barriers with a high number of TJ strands, high conductance, and wide DP ranges. These TJ barriers are associated with high paracellular channel activity, with varying selectivity for ions. Cldn2, Cldn15, and Cldn10b exhibit high, moderate, and low cation selectivity, respectively, while Cldn10a exhibits anion selectivity.

#### Class 3: Cldns forming “nonautonomous TJ barriers”

Class 3 comprises Cldn8, 12, 13, 16, 17, and 20 to 27. These Cldns do not form autonomous TJ barriers, lacking TJ strands and exhibiting conductance and DP similar to *Cldn*-null cells. As shown by expression in WT cells (fig. S10) and partly consistent with previous reports, they integrate into preexisting TJs, if present, and become functional (*1*–*8*).

#### Class 4: Cldn forming an “acid-enhancible TJ barrier”

**Fig. 5.**
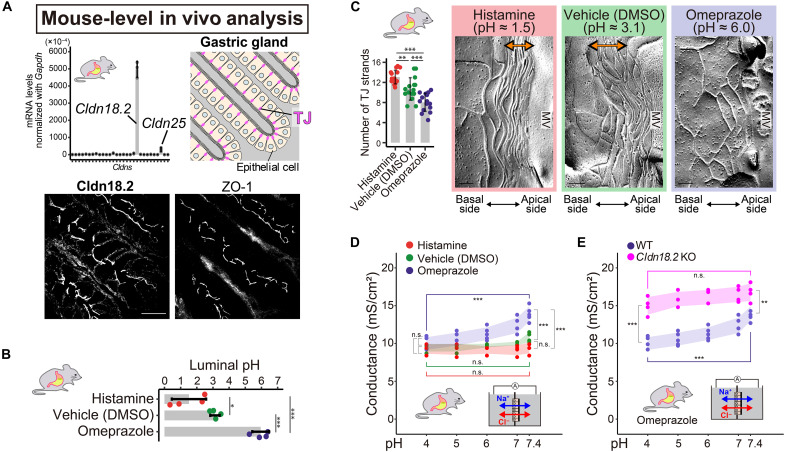
An acidic environment strengthening TJ barriers in the stomach in vivo. (**A**) qPCR of *Cldns* and IF of Cldn18.2 with ZO-1 in WT mouse stomachs (*n* = 3 mice). Scale bar, 20 μm. (**B** and **C**) Luminal pH (B) (*n* = 4 mice) and FF of histamine-, omeprazole-, or vehicle [dimethyl sulfoxide (DMSO)]–treated WT mouse stomachs (C) (*n* = 15 micrographs from four mice). Bidirectional arrows: dense TJ strands. Scale bars, 100 nm. The FF image size is normalized for consistent visualization of TJ strands, as the thickness of each TJ strand varies because of fluctuations in carbon and platinum shadowing. Values are means ± SDs. *P < 0.05, **P < 0.01, and ***P < 0.001 (Fisher’s one-way ANOVA with the Tukey-Kramer multiple-comparison test). (**D** and **E**) pH-dependent electrical conductance of gastric mucosa from WT mice treated with histamine, omeprazole, or vehicle (DMSO) (D) or from omeprazole-treated WT and *Cldn18.2* KO mice (E) (*n* = 4 to 5 samples). To assess pH-dependent changes, Fisher’s one-way ANOVA with the Tukey-Kramer multiple-comparison test was used for omeprazole-treated mice, and the Kruskal-Wallis test was used for histamine- and vehicle-treated mice. To evaluate treatment-dependent differences, Fisher’s one-way ANOVA with the Tukey-Kramer multiple-comparison test (at pH 7.4) and Kruskal-Wallis test (at pH 4.0) was used in (D), and Student’s *t* test was used in (E). Values are means ± SDs (error bands). **P < 0.01, ***P < 0.001, and n.s.: not significant.

Class 4 comprises Cldn18.2, which autonomously forms weak TJ barriers with few TJ strands, high conductance, and no DP at neutral pH; however, unlike other Cldns, this barrier is uniquely strengthened and significantly enhanced in acidic environments (Fig. 5 and fig. S11A). Our classification of Cldn family members (Fig. 4E, fig. S9, and movie S5) comprehensively elucidates their functional diversity, shaping TJ barrier diversity.

### In vivo homeostatic cues shape TJ barrier diversity

In addition to the intrinsic properties of individual single Cldns, various environmental factors are thought to influence the diversity of TJ barriers in vivo. Here, we examined TJ barrier changes in the stomach due to gastric acids as the homeostatic cue.

Our classification demonstrated that Cldn18.2, predominantly expressed in the stomach and highly enriched at TJs of mouse gastric glands (Fig. 5A), belongs to class 4, forming a weak paracellular barrier at neutral conditions (Fig. 4), contrasting with the expectation that it would form acid-resistant strong barriers (*22*). We stimulated or inhibited gastric acid secretion in vivo by histamine or omeprazole, leading to luminal pH of 1.5 or 6.0, respectively (Fig. 5B). Despite similar IF localization of Cldn18.2 at TJ across treatments (fig. S11A), FF revealed the gastric acid–enhanced polymerization and dense packing of TJ strands with histamine at pH 1.5 (Fig. 5C), suggesting an acid-enhancible TJ barrier.

In Ussing chamber experiments, omeprazole-treated WT stomachs exhibited high transmucosal conductance at pH 7.4, which decreased as the luminal pH shifted to 4.0 (Fig. 5D). In contrast, control and histamine-treated stomachs maintained low conductance at pH 7.4. These findings suggested that the Cldn18.2-based barrier was well developed under acidified conditions. Consistently, stomachs from *Cldn18.2* KO mice treated with omeprazole displayed higher conductance and negligible acid sensitivity compared to WT stomachs, highlighting Cldn18.2’s role in acid sensitivity (Fig. 5E).

**Fig. 6.**
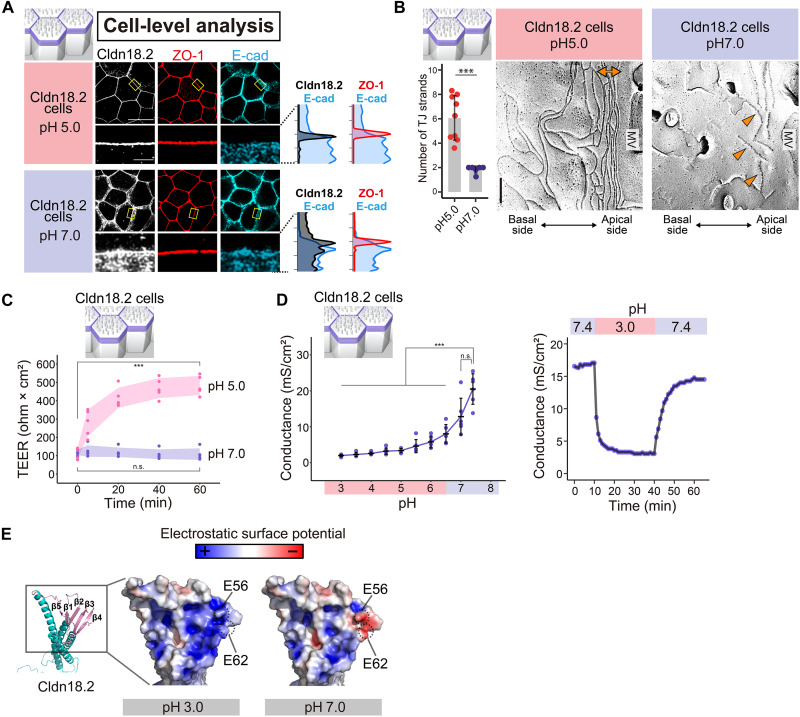
An acidic environment strengthening TJ barriers in single *Cldn18.2*-expressing cells. (**A**) IF of Cldn18.2 with ZO-1 and E-cad in single *Cldn18.2*-expressing cells (Cldn18.2 cells) at pH 5.0 and 7.0. Magnified views with intensity plots (bottom). Scale bars, 10 μm (top) and 1 μm (bottom). (**B**) FF of Cldn18.2 cells at pH 7.0 and 5.0 (*n* = 4 to 9 micrographs). Bidirectional arrow: dense TJ strands; arrowheads: partial strand polymerization. Scale bar, 200 nm. ***P < 0.001 (Brunner-Munzel test). (**C** and **D**) pH-dependent changes of TEER [*n* = 5 wells (C)] and electrical conductance [*n* = 6 wells (D); left] in Cldn18.2 cells. Time courses of the TEER values from 0 to 60 min are shown in (C). To assess time-dependent changes in TEER values, Fisher’s one-way ANOVA with the Tukey-Kramer multiple-comparison test was used (C). Values are means ± SDs (error bands). pH-dependent changes in the electrical conductances were assessed using Steel’s multiple-comparison test (D; left). Values are means ± SDs. Temporal dynamics of conductance in response to pH shifts in Cldn18.2 cells are also shown in (D; right). ***P < 0.001 and n.s.: not significant. (**E**) The extracellular β sheet (β1 to β5) of Cldn18.2, with negatively charged E56 and E62 at pH 7.0 (red), neutralized at pH 3.0 (white).

In cell-level analyses, at pH 5.0, Cldn18.2 concentrated at TJs alongside ZO-1, while at pH 7.0, it exhibited a diffuse distribution (Fig. 6A). FF revealed acid-enhanced polymerization and dense packing of TJ strands at pH 5.0 but only partially polymerized strands at pH 7.0 (Fig. 6B). Acidifying the apical medium to pH 5.0 increased TEER (Fig. 6C). Ussing chamber experiments showed that conductance dropped rapidly when the apical pH shifted from 7.4 to 3.0 and reversed upon returning to pH 7.4 (Fig. 6D). DP was negligible at pH 3.0 and 7.4 (fig. S11B). Furthermore, among the Cldn1 to 27 cells, none exhibited as pronounced an increase in TEER under pH 5.0 conditions as the Cldn18.2 cells (fig. S11C). These results strongly suggested that acid sensitivity is an intrinsic property of Cldn18.2, classifying it as class 4.

To uncover the structural basis of acid sensitivity in the model, we focused on residues E56 and E62 in Cldn18.2’s extracellular β sheet. These residues, which are implicated in ion permeability, were negatively charged at pH 7.0 but became neutral upon protonation at pH 3.0 (Fig. 6E). Protonation likely promotes Cldn18.2 polymerization for the TJ barrier function. These findings suggest that homeostatic cues can markedly modify the properties of individual single Cldns.

Our classification of Cldns offers valuable insights into interpreting their functions within TJ barriers in vivo. However, applying this knowledge remains an issue for future investigation.

## DISCUSSION

Our comprehensive analyses of single Cldn family member–expressing cells, based on *Cldn*-null cell backgrounds, revealed profound functional diversity among Cldn family members and classified the 27 members into four main classes (classes 1 to 4) based on morphological and functional aspects. These results partly align with a recent classification using super-resolution stimulated emission depletion microscopy, due to its specific focus on morphology (*45*). Our single Cldn analyses, which are highly consistent with the molecular model (*15*–*18*), provide a foundation for understanding and manipulating TJs composed of multiple Cldns in vivo, offering an opportunity to revisit and reinterpret previous findings.

Among class 1 Cldns, deficiencies in Cldn3 to 7, 11, and 18.1 have been linked to cancers, colitis, infertility, respiratory infections, neurological disorders, and other disorders, as demonstrated largely by KO mouse studies (*11*, *14*, *23*, *44*, *46*). We revealed that these Cldns autonomously form robust TJ barriers, further supporting their roles in pathomechanisms.

Our observation of Cldn4 autonomously forming TJ strands in epithelial cell lines contrasts with previous studies reporting that Cldn4 does not form TJ strands in nonepithelial cells (*45*, *47*). Nevertheless, the fragmentary TJ strands and the relatively diffuse distribution of Cldn4 around TJs in our data are at least partly consistent with those reports. These findings seem to be consistent with the description in the previous report that Cldn4’s direct contributions to barrier function are minimal (*47*).

Notably, class 1 Cldn1 and Cldn19 form TJ barriers with minimal and low cation-selective paracellular channel activities, respectively. Cldn1’s behavior is particularly surprising, given its reported role as a strong water barrier in skin (*9*, *12*), although it may be reasonable considering the moist nature of the skin. Conversely, Cldn19’s function may align with previous mutation studies on familial hypomagnesemia with hypercalciuria and nephrocalcinosis (*48*). Class 1 Cldn14 forms TJ barriers with low anion-selective paracellular channel activity, also supporting its reported role as a cation blocker in the kidney (*49*).

Class 2 Cldns (Cldn2, 10a, 10b, and 15) exhibit channel activity, consistent with previous studies on their channel-forming activity in the kidney, liver, and intestine (*31*–*35*). We found that Cldn18.2 in class 4 forms an acid-enhancible TJ barrier in the stomach in vivo, highlighting its dynamic, environment-responsive nature.

Class 3 Cldns (Cldn8, 12, 13, 16, 17, and 20 to 27) do not autonomously form TJ barriers, although Cldn8, 12, 16, and 17 are reportedly involved in selective ion transport in the kidney and Cldn21 acts as a cation-selective paracellular channel (*29*, *48*, *50*–*52*). Thus, even for Cldns that do not autonomously form TJ barriers, their cooperation with other Cldns likely contributes to essential TJ functions in vivo.

Most epithelial cells, both in vivo and in culture, coexpress multiple Cldns. Therefore, understanding how TJ strands form under the combined expression of Cldns is critical for elucidating Cldn function in vivo. For example, it was reported that in nonepithelial cultured cells, neither Cldn4 nor Cldn8 alone formed TJ strands, whereas their coexpression resulted in TJ strand formation (*45*). Furthermore, Cldn4 was shown to disrupt TJ strands formed by Cldn2, 7, 15, and 19, increasing TEER by antagonizing the channel functions of Cldn2 and Cldn15 (*47*). In addition, coexpression of Cldn16 and Cldn19 is required for forming a paracellular channel permeable to divalent cations in cultured epithelial cells (*48*). Cldn23 has been reported to be associated with other Cldns, particularly Cldn3 and Cldn4, likely forming heteromeric complexes that contribute to TJ strand organization in cultured epithelial cells (*53*). More recently, Cldn25 was implicated in the modulation of TJ barrier function (*54*). Collectively, these findings point to the importance of Cldn-Cldn interactions in TJ organization and function. Our data further suggest a role for class 3 Cldns in modulating TJ barrier function.

In vivo studies using genetically modified mice—including KO, knockdown, and transgenic models—have implicated the roles of individual Cldns (Cldn1 to 5, 7, 11, 14, 16, 18.1, 18.2, and 19), revealing alterations in TJ barrier or paracellular channel functions associated with various pathological conditions such as inflammation, cancer, metabolic disorders, and deafness (*2*, *34*). The *Cldn*-null cell-based system for TJ reconstitution developed in this study is expected to serve as a valuable tool for future mechanistic analyses, as it enables systematic evaluation of the effects of single or combined Cldn expression on TJ barrier and paracellular channel functions.

It has been reported that the expression of various Cldns is either up-regulated or down-regulated in different cancer types (*55*). In this context, antibodies targeting Cldns are attracting increasing attention as potential therapeutic agents. In particular, an anti-Cldn18.2 antibody has already been developed and approved for the treatment of gastric cancer (*39*). This is closely related to the fact that Cldn18.2 is specifically expressed in the gastric epithelium and that during epithelial-to-mesenchymal transition, malignant transformation leads to a loss of cell polarity, resulting in altered expression and mislocalization of Cldn18.2 away from TJs. This mislocalized Cldn18.2 is thought to become accessible to antibody binding, thereby inducing cytotoxic effects via antibody-dependent cellular cytotoxicity. However, the precise mechanisms underlying the antitumor effects of these antibodies remain to be fully elucidated.

Although the comprehensive characterization of all 27 Cldn family members—whether expressed individually or in combination—remains an ongoing challenge, our findings lay the foundation not only for understanding the basic biology of Cldn-based TJ function but also for developing Cldn-targeted therapeutic strategies to promote health, prevent disease, and improve clinical management.

## MATERIALS AND METHODS

### EpH4, CSG120/7, MTD-1A, Lenti-X 293T, and *ZO-1*/*ZO-2* double KO cells

EpH4 mouse mammary gland epithelial cells (RRID: CVCL_0073) (*56*) were a gift from E. Reichmann (Research Institute of Molecular Pathology, Vienna, Austria). CSG120/7 mouse salivary gland carcinoma cells (*57*) were a gift from C. Birchmeier (Max Delbrück Center for Molecular Medicine, Berlin, Germany). MTD-1A mouse mammary gland epithelial cells (RRID: CVCL_EG11) (*58*) were a gift from M. Takeichi. Lenti-X 293T cells were purchased from Takara Bio Inc. (Shiga, Japan; catalog no. 632180). *ZO-1*/*ZO-2* double KO cells were previously established from *ZO-1* KO EpH4 cells by CRISPR-Cas9 genome editing (*42*, *59*). Cells were maintained in Dulbecco’s modified Eagle’s medium (DMEM) (Shimadzu Diagnostics Corporation, Tokyo, Japan; catalog no. 05919) supplemented with 10% (v/v) fetal bovine serum (FBS) (Thermo Fisher Scientific, Waltham, MA, USA; catalog no. 10270-106), 2 mM l-glutamine, and 0.45% (w/v) d(+)-glucose at 37°C with 5% CO_2_.

### *Cldn*-null cells

For efficient KO of eight endogenously expressed *Cldn* genes in EpH4 cells (WT cells), we used a multiplex CRISPR-Cas9–based genome editing system. This system uses an all-in-one CRISPR-Cas9 vector containing one Cas9 nuclease and up to seven guide RNA (gRNA) expression cassettes for simultaneous targeting of multiple genomic loci (*60*).

Two types of all-in-one CRISPR-Cas9 vectors were initially constructed using the Multiplex CRISPR/Cas9 Assembly System Kit (Addgene, Watertown, MA, USA; catalog no. 1000000055): Vector 1 contained six gRNA expression cassettes targeting *Cldn3*, *4*, *7*, *9*, *12*, and *25* genes, while vector 2 contained four gRNA expression cassettes targeting *Cldn4*, *8*, *23*, and *25* genes. Vectors 1 and 2 were simultaneously transfected into WT cells along with a *pPGK-puro* plasmid (Addgene; catalog no. 11349) using Lipofectamine 3000 (Thermo Fisher Scientific, Waltham, MA, USA; catalog no. L3000001). After 5 to 7 days of selection with puromycin dihydrochloride (5 μg/ml) (Sigma-Aldrich Inc., St. Louis, MO, USA; catalog no. P8833), puromycin-resistant colonies (70 colonies) were isolated and screened for mutations in the eight *Cldn* genes using a T7 endonuclease 1 assay (*61*). Among 70 colonies, 11 exhibited relevant mutations and were subcloned by limiting dilution. Following genomic DNA sequencing of the resulting single-cell–derived clones (90 clones), frameshift insertion/deletion (indel) mutations were detected in *Cldn3*, *4*, *8*, *9*, *23*, and *25*, except for *Cldn7* and *12,* in one candidate cell clone. Subsequently, an additional all-in-one CRISPR-Cas9 vector containing gRNAs targeting *Cldn7* and *12* (designated as an all-in-one CRISPR-Cas9 vector 3) was transfected into the candidate clone along with a *pTK-Hyg* vector (Takara Bio Inc., Shiga, Japan; catalog no. 631750) using Lipofectamine 3000. After selection with a medium containing hygromycin B (200 μg/ml) (FUJIFILM Wako Pure Chemical Corporation, Osaka, Japan; catalog no. 080-07683), hygromycin B–resistant colonies (10 colonies) were screened for mutations in *Cldn7* and *12* using the T7 endonuclease 1 assay and DNA sequencing. One candidate colony was subcloned by limiting dilution, resulting in 10 single-cell–derived clones. DNA sequencing definitively identified frameshift indel mutations in *Cldn7* and *12* and in *Cldn3*, *4*, *8*, *9*, *23*, and *25*, in one cell clone, indicating the establishment of *Cldn*-null cells.

### Single *Cldn*-expressing cells

Lentivirus-mediated gene transduction was used to introduce each mouse *Cldn* gene (with or without tagging) into *Cldn*-null cells, generating a series of single *Cldn*-expressing epithelial cells. Lentiviruses carrying individual *Cldn* gene were prepared by transfecting the *pLVSIN-CMV Neo* vector (Takara Bio Inc.; catalog no. 6181) containing each *Cldn* gene (*pLVSIN Cldn1–27*) with a Lentiviral High Titer Packaging Mix (Takara Bio Inc.; catalog no. 6194) into Lenti-X 293T cells (Takara Bio Inc.; catalog no. 632180). A mixture of 1 μg of *pLVSIN Cldn* plasmid and 1.3 μl of Lentiviral High Titer Packaging Mix was combined with 60 μl of PEI MAX transfection reagent (Polysciences Inc., Warrington, PA, USA; catalog no. 24765-1) and 300 μl of Opti-MEM I Reduced Serum Medium (Thermo Fisher Scientific; catalog no. 31985070) following the manufacturer’s instructions. This mixture was added to Lenti-X 293T cells cultured at 80% confluency in 10 ml of medium with 10% (v/v) FBS. After 48 hours posttransfection, 20 ml of lentivirus-containing medium was harvested and filtered through a polyethersulfone (PES) filter with 0.45-μm pore sizes (Merck, Darmstadt, Germany; catalog no. SLHP033NK). After filtration, the medium was incubated with 10 mM Hepes (pH 7.5), 8% (v/v) polyethylene glycol, molecular weight 6000 (FUJIFILM Wako Pure Chemical Corporation; catalog no. 169-09125), and 100 mM NaCl at 4°C overnight to precipitate lentiviruses. Precipitates were collected by centrifugation at 440*g* and 4°C for 30 min and gently suspended with 500 μl of medium by pipetting. The lentiviruses in the suspension were then used to infect *Cldn*-null cells, which were cultured at a density of 0.5 × 10^5^ cells per well in a 12-well culture plate. At 24 hours postinfection, the medium was replaced with a fresh medium containing G418 (500 μg/ml) (Nacalai Tesque, Kyoto, Japan; catalog no. 09380-44). G418-resistant cells underwent subcloning by limiting dilution to obtain single-cell–derived clones in the presence of G418. These clones, which stably expressed a single *Cldn*, underwent screening using immunoblot and IF analyses, leading to the establishment of two or three distinct cell clones.

### Mice

Eight-week-old male WT C57BL/6J mice, purchased from Japan SLC, were used. *Cldn18.2* KO mice were previously generated (*22*, *62*). Eight-week-old male KO mice were used. Mice were maintained in a specific pathogen–free environment with a 12-hour light/dark cycle and provided ad libitum access to water and standard chow. Experiments were approved by the Animal Experiment Committee of Teikyo University (TDR 22-022) and Osaka University (FBS-17-003).

### Plasmids

To generate *Cldn*-null epithelial cells, all-in-one CRISPR-Cas9 vectors were constructed using the Multiplex CRISPR/Cas9 Assembly System Kit (Addgene, Watertown, MA, USA; catalog no. 1000000055). According to a previously described protocol (*60*), sense and antisense oligonucleotides of a gRNA expression cassette targeting each *Cldn* gene were annealed and inserted into the BbsI-digested *pX330A* or *pX330S* vector (Addgene; catalog no. 1000000055). The oligonucleotides were designed using the online CRISPR design tool (https://crispr.dbcls.jp) (*63*). These vectors, each containing a single gRNA expression cassette, were assembled into an all-in-one vector harboring multiple gRNA cassettes using the Golden Gate assembly technique (*60*).

To express Cldns, the cDNA of each of the 27 mouse *Cldn* family members was amplified by polymerase chain reaction (PCR) using PrimeSTAR Max DNA Polymerase (Takara Bio Inc., Shiga, Japan; catalog no. R045A) and subcloned into the *pLVSIN-CMV Neo* vector (Takara Bio Inc.; catalog no. 6181) using the In-Fusion HD Cloning Kit (Takara Bio Inc.; catalog no. 639650), yielding *pLVSIN Cldn1–27*. Oligonucleotides of *Flag* sequences were synthesized (FASMAC Co. Ltd., Kanagawa, Japan) and subcloned into *pLVSIN Cldn12*, *13*, *16*, *17*, and *20 to 27* using the In-Fusion HD Cloning Kit to obtain *pLVSIN Flag-Cldn12*, *13*, *16*, *17*, and *20 to 27*, respectively. *EGFP* sequences were amplified by PCR using PrimeSTAR Max DNA Polymerase from the *pCAGGS-EGFP* vector and subcloned into *pLVSIN Cldn3* using In-Fusion cloning to generate *pLVSIN EGFP-Cldn3*. In addition, several “silent mutations” were introduced into the PAM or gRNA recognition sequences of *pLVSIN Cldn3*, *4*, *7*, *8*, and *9* and *pLVSIN Flag-Cldn12*, *23*, and *25* and *pLVSIN EGFP-Cldn3* to prevent CRISPR-Cas9 genome editing.

To express the glutathione *S*-transferase (GST)–Flag fusion protein and GST-fusion protein of the C-terminal domain of mouse Cldn1 to 12, 14, 15, 18, and 19, the oligonucleotides of *Flag* sequences were synthesized (FASMAC Co. Ltd.), and C-terminal cytosolic domain of mouse *Cldn1* to *12, 14, 15, 18, and 19* were amplified using PCR PrimeSTAR Max DNA Polymerase from *pLVSIN Cldn1* to *12, 14, 15, 18, and 19*, respectively. Subsequently, they were subcloned into the *pGEX-6P-2* vector (Merck, Darmstadt, Germany; catalog no. GE28-9546-50) using the In-Fusion HD Cloning Kit. This resulted in the generation of *pGEX-6P-2 Flag* and *pGEX-6P-2 Cldn1* to *12, 14, 15, 18, and 19 C-term* constructs. All constructed plasmids were subjected to DNA sequencing to ensure accuracy.

### Quantitative real-time PCR

For relative quantification of mRNA expression levels in cell lines, cells were seeded in a 12-well plate and cultured for 10 days at 37°C with 5% CO_2_. Total RNA was isolated using the RNeasy Mini Kit (QIAGEN N.V., Limburg, Netherlands; catalog no. 74104), and cDNA was synthesized using ReverTra Ace qPCR RT Master Mix with gDNA Remover (TOYOBO Co. Ltd., Osaka, Japan; catalog no. FSQ-301). Quantitative real-time PCR was performed using PowerUP SYBR Green Master Mix for qPCR (Thermo Fisher Scientific; catalog no. A25742) and the QuantStudio 5 Real-Time PCR System (384-well block; Thermo Fisher Scientific; catalog no. A28140). Each reaction contained 2.5 ng of cDNA and 500 nM forward and reverse primers targeting each gene (table S1). The relative gene expression levels were calculated by comparing the *C*_t_ (cycle threshold) values of the target genes to those of *Gapdh*. For relative quantification of mRNA expression levels in mouse organs, organs from 8-week-old male C57/BL6J mice were cut into pieces of ~3 mm by 3 mm and immersed in RNAlater Stabilization Solution (Thermo Fisher Scientific; catalog no. AM7020). The organs were then frozen in liquid nitrogen and crushed using a cryomill (Tokken Inc., Chiba, Japan; catalog no. SK-100). The resulting specimens underwent RNA isolation using the RNeasy Mini Kit. The brain, pituitary gland, and lung were immersed directly in TRIzol Reagent (Thermo Fisher Scientific; catalog no. 15596026) and homogenized using a plastic pestle. Total RNA was purified according to the protocol for TRIzol Reagent. cDNA synthesis and quantitative real-time PCR analysis were performed as described above.

For absolute quantification of the mRNA expression level of *Cldn* in each respective single *Cldn*-expressing cell line, quantitative real-time PCR was performed as described above, using 10 ng of cDNA per reaction. Absolute quantification was achieved by generating standard curves from a 10-fold serial dilution of plasmid DNA, with concentrations ranging from 1 × 10^8^ to 1 × 10^2^ copies per reaction. The plasmids used as standards were as follows: *pLVSIN Cldn1*, *pLVSIN Cldn19*, and *pLVSIN Flag* (for *Cldn1*, *Cldn19*, and *Flag-Cldn12*, *13*, *16*, *17*, and *20* to *27* mRNA quantification, respectively) (table S1). mRNA expression levels were calculated from the standard curve and expressed as copies per 1 ng of cDNA. Specific primers targeting mRNAs of *Cldn1*, *Cldn19*, and *Flag* (for *Flag-Cldn12*, *13*, *16*, *17*, and *20* to *27*) were used, respectively (table S1).

### RNA sequencing

Cells were seeded on a semipermeable polyethylene terephthalate (PET) membrane filter (Greiner Bio-One Co. Ltd., Baden-Württemberg, Germany; catalog no. 662641) featuring 0.4-μm pores and providing a growth area of 1.13 cm^2^ and cultured for 7 days at 37°C with 5% CO_2_. Total RNA was purified from the cells using the RNeasy Mini Kit (QIAGEN N.V.; catalog no. 74104). The quality of the samples was assessed by Takara Bio Inc. (Shiga, Japan) using a Fragment Analyzer, demonstrating RNA integrity number (RIN) values ≥ 7. RNA-seq was performed by Takara Bio Inc. using the NovaSeq X Plus platform to generate paired-end reads. For the processing of RNA-seq data, adapter sequences were first removed, and reads were quality trimmed using Trimmomatic (version 0.36). The trimming process involved removing Nextera adapter sequences, trimming low-quality bases from both the leading and trailing ends, and applying a sliding window trimming approach. The minimum length threshold for reads after trimming was set to 50 bases. Posttrimming, the quality of the reads was assessed using FastQC (version 0.11.9). The reference genome for *Mus musculus* (GRCm39) was downloaded from the Ensembl database and used for subsequent alignment and gene expression quantification. Trimmed reads were aligned to the reference genome using STAR (version 2.7.9a) with the aligner configured to run in paired-end mode using 10 threads. The output files included sorted BAM files and transcriptome alignments for downstream analysis. Gene expression levels were quantified using RSEM (version 1.3.3), with quantification performed in paired-end mode, specifying reverse strandedness. For the differential gene expression analysis, we used the edgeR package (version 3.42.4). Gene counts were normalized using the calcNormFactors function, and mRNA expression levels were quantified primarily as transcripts per million (TPM). Genes with low expression were filtered out by retaining only those with a counts-per-million value above 10 in at least one sample. A linear model was fitted using the voom method from the limma package (version 3.56.2), and differential expression was tested using an empirical Bayes approach.

### Purification of genomic DNA, T7 endonuclease 1 assay, and DNA sequencing

Cells were plated onto a 12-well plate and incubated for 5 days at 37°C with 5% CO_2_. Subsequently, cell lysis was performed using 300 μl of a solution consisting of 0.2% (w/v) SDS, 6 mAnson U/ml proteinase K (Takara Bio Inc., Shiga, Japan; catalog no. 9034), 5 mM EDTA, 200 mM NaCl, and 100 mM tris-HCl (pH 8.0) for 60 min at 60°C. To precipitate the genomic DNA, 300 μl of isopropanol was added to the lysate, followed by centrifugation at 20,400*g* for 30 min at 4°C. The resulting genomic DNA pellet was then treated with 1 ml of 70% (v/v) ethanol, centrifuged again at 20,400*g* for 5 min at 4°C, and air dried for 15 min at room temperature. The pellet was reconstituted in 50 μl of Tris-EDTA (TE) buffer to serve as the genomic DNA sample. Subsequently, PCR was performed using 100 ng of the genomic DNA sample as a template. The resulting PCR products underwent purification using ExoSAP-IT PCR Product Cleanup Reagent (Thermo Fisher Scientific; catalog no. 78200.200.UL) according to the manufacturer’s protocol. Last, the purified samples underwent a T7 endonuclease 1 assay using T7 Endonuclease I reaction Mix (NIPPON GENE Co. Ltd., Tokyo, Japan; catalog no. 313-08801). In certain cases, the purified samples were sent to FASMAC Co. Ltd. for Sanger sequencing.

### Antibody

The rabbit polyclonal anti-Cldn23 antibody was raised against the C-terminal cytosolic region of mouse Cldn23, corresponding to amino acids 183 to 296. The rat monoclonal anti-Cldn25 antibody was produced against the extracellular region of mouse Cldn25, corresponding to amino acids 41 to 60. All procedures were approved by the Animal Experiment Committee of Osaka University. Other antibodies were previously generated or are commercially available, as detailed in table S1.

### Immunoblotting

Cells were seeded in a six-well plate and cultured for 10 days at 37°C with 5% CO_2_. The cells were lysed using SDS sample buffer containing 62.5 mM tris-HCl (pH 6.8), 2% (w/v) SDS, 5% (v/v) glycerol, and 0.0125% bromophenol blue. Subsequently, the lysate underwent sonication for 30 s using a Sonifier 250 (Branson), operating at an output level of 1 with a 50% duty cycle. After sonication, the sample was incubated on ice for 30 s before undergoing another round of sonication. The sample was centrifuged at 20,400*g* for 10 min at 4°C, and the amount of total proteins in the resulting supernatant was determined using the Pierce 660-nm Protein Assay Kit (Thermo Fisher Scientific; catalog no. 22662), with a dilution series of bovine serum albumin (BSA) as standards. Following this, 10 μg of total proteins, supplemented with dithiothreitol to a final concentration of 50 mM, underwent SDS–polyacrylamide gel electrophoresis (SDS-PAGE) without boiling. Subsequently, they were transferred to a polyvinylidene difluoride (PVDF) membrane with 0.45-μm pores (Merck; catalog no. IPVH00010) at a constant current of 150 mA for Cldn detection and 500 mA for ZO-1, occludin (Ocln), tricellulin (Tric), junctional adhesion molecule-A (JAM-A), E-cadherin (E-cad), α-catenin, and vinculin (Vcl) detection for 60 min on ice. In addition, to quantify Cldn and Flag-Cldn expressions, dilution series of purified GST-Cldn C-terminal or GST-Flag proteins served as standards after SDS-PAGE and transfer to the same PVDF membrane.

After blocking with a blocking buffer containing 5% (w/v) nonfat skim milk in tris-buffered saline (TBS) with 0.05% Tween 20 (TBS-T), the membrane was incubated with a primary antibody in the blocking buffer or Can Get Signal Solution 1 (TOYOBO Co. Ltd., Osaka, Japan; catalog no. NKB-101) for 90 min at room temperature. After three 10-min TBS-T washes, the membrane was incubated with a horseradish peroxidase (HRP)–conjugated secondary antibody in blocking buffer or Can Get Signal Solution 2 (TOYOBO Co. Ltd.; catalog no. NKB-101) for 60 min at room temperature. Following another three washes with TBS-T for 10 min each, the membrane was treated with Immobilon Western Chemiluminescent HRP Substrate (Merck; catalog no. WBKLS0100), and chemiluminescence signals were visualized using an imager (Cytiva; catalog no. Amersham Imager 680) and quantified using the ImageJ software (National Institutes of Health). Antibodies used in the experiments are detailed in table S1.

### IF microscopy

For IF analyses, cells were seeded on a semipermeable PET membrane filter (Greiner Bio-One Co. Ltd., Baden-Württemberg, Germany; catalog no. 662641) featuring 0.4-μm pores and providing a growth area of 1.13 cm^2^ and cultured for ~14 days at 37°C with 5% CO_2_. The cells were fixed with ice-cold methanol for 5 min at −20°C. Subsequently, they were blocked with 1% (v/v) FBS in Hanks’ balanced salt solution (HBSS) for 10 min at room temperature. Next, the cells were incubated with primary antibodies in blocking buffer or Can Get Signal Immunoreaction Enhancer Solution A (TOYOBO Co. Ltd.; catalog no. NKB-501) for 60 min at room temperature, followed by three washes of 10 min each with HBSS. Last, they were incubated with secondary antibodies in blocking buffer for 60 min at room temperature. Following three washes (10 min each) with HBSS, the cells were rinsed with Milli-Q water and mounted using ProLong Diamond Antifade Mountant (Thermo Fisher Scientific; catalog no. P36961). IF images were captured using a spinning disk super-resolution confocal microscope (Evident, Tokyo, Japan; catalog no. SpinSR10) with silicon oil-immersion objective lenses (UPLSAPO60XS2 or UPLSAPO100XS, Evident), a CSU-W1 SoRa-Unit (Yokogawa Electric Corporation, Tokyo, Japan), and an ORCA-Flash4.0 V3 digital CMOS camera (Hamamatsu Photonics, Shizuoka, Japan), controlled by MetaMorph software Ver. 7.1.0.1.161 (Molecular Devices, USA). Selected images were then subjected to deconvolution using cellSens Dimension Desktop 3.2 (Evident, Japan).

For IF analyses, freshly isolated mouse organs were embedded in Tissue-Tek O.C.T. Compound (Sakura Finetek Japan Co. Ltd., Tokyo, Japan; catalog no. 4583) and promptly frozen using liquid nitrogen. The frozen specimens were sectioned to a thickness of 5 μm at −20°C using a cryostat (Leica Microsystems, Hesse, Germany; catalog no. CM1850). Subsequently, the sections were air dried for 10 min at room temperature and fixed with ice-cold methanol for 5 min at −20°C. Following three washes with phosphate-buffered saline (PBS), the sections were blocked with 1% (w/v) BSA in PBS for 10 min at room temperature. The sections were subsequently incubated with suitable primary and secondary antibodies, followed by IF analyses using a spinning disk super-resolution confocal microscope following the procedures described above. The antibodies used in the experiments are detailed in table S1.

### Purification of the GST-flag protein and GST-fusion protein with the C-terminal domain of Cldn

To purify the GST-Flag protein (GST-Flag) or GST-fusion protein of the C-terminal domain of Cldn (GST-Cldn C-term), BL21 *Escherichia coli* (Takara Bio Inc., Shiga, Japan; catalog no. 9126), transformed with *pGEX-6P-2 Flag* or *pGEX-6P-2 Cldn1* to *12, 14, 15, 18, and 19 C-term*, was cultured in 200 ml of LB medium supplemented with ampicillin (100 μg/ml) (Nacalai Tesque, Kyoto, Japan; catalog no. 19769-64) at 37°C with agitation at 180 rpm for 3 hours. Protein expression was induced by adding 1 mM isopropyl-β-D-thiogalactopyranoside (Nacalai Tesque; catalog no. 19742-94) to the culture, followed by incubation overnight at 16°C with agitation at 180 rpm. The cells were then harvested by centrifugation at 6000*g* and 4°C for 10 min. The cell pellets were resuspended in 15 ml of ice-cold radioimmunoprecipitation assay (RIPA) buffer containing 50 mM tris-HCl (pH 7.5), 150 mM NaCl, 0.5% (w/v) sodium deoxycholate, 0.1% (w/v) SDS, and 1% (v/v) Nonidet P-40, supplemented with a protease inhibitor cocktail (Nacalai Tesque; catalog no. 03969-21). Samples were sonicated for 1 min using a Sonifier 250 (Branson) set at an output level of 1 with a constant duty cycle. The lysate was incubated on ice for 10 min, followed by centrifugation at 6000*g* at 4°C for 20 min. The supernatant was then incubated with 300 μl of glutathione Sepharose 4B (Cytiva, Marlborough, MA, USA; catalog no. GE17-0756-01) at 4°C for 120 min. The Sepharose was washed five times with 10 ml of RIPA buffer, and the GST-Flag protein or GST-fusion protein of the C-terminal domain of Cldn bound to Sepharose was eluted with 1 ml of a buffer containing 20 mM reduced glutathione (FUJIFILM Wako Pure Chemical Corporation; catalog no. 073-02013) and 50 mM tris-HCl (pH 8.0). GST-Flag and GST-Cldn C-term proteins were analyzed for purity and concentration by SDS-PAGE. Following this, images of a polyacrylamide gel stained with Coomassie Brilliant Blue (CBB) R-250 (Nacalai Tesque; catalog no. 09408-52) were obtained using a scanner (Seiko Epson Corporation, Nagano, Japan; catalog no. GT-X970). Quantitative densitometry of the CBB-stained proteins was performed using the ImageJ software (National Institutes of Health, Bethesda, MD, USA), with a dilution series of BSA (Nacalai Tesque; catalog no. 01860-36) standards for protein quantification.

### Thin-section electron microscopy

Cells were seeded onto a semipermeable PET membrane filter featuring 0.4-μm pores and providing a growth area of 1.13 cm^2^ at a density of 0.89 × 10^5^ cells/cm^2^ and cultured for ~14 days at 37°C with 5% CO_2_. Subsequently, they were fixed with a fixative solution containing 2.5% (v/v) EM-grade glutaraldehyde (TAAB Laboratories Equipment Ltd., Berkshire, England; catalog no. G004), 2.0% (w/v) paraformaldehyde (FUJIFILM Wako Pure Chemical Corporation; catalog no. 168-23255), and 100 mM Hepes (pH 7.4) for 1 hour at room temperature. After three washes with 100 mM Hepes (pH 7.4) buffer for 10 min each, the cells were postfixed with 1% (w/v) OsO_4_ [Heraeus South Africa (Pty) Ltd., Eastern Cape, South Africa; catalog no. 5027800] in 100 mM Hepes buffer (pH 7.4) for 2 hours on ice. Following three additional rinses with Milli-Q water, the cells were stained with 1% (w/v) uranyl acetate for 1 hour at room temperature. The cells were dehydrated using a graded series of ethanol [55, 65, 75, 85, 95, and 99.5% (v/v)], followed by absolute ethanol three times for 10 min each and propylene oxide twice for 10 min each. The dehydrated cells were immersed overnight at room temperature in a mixture of the Epon Quetol-812 (Nisshin EM Co. Ltd., Tokyo, Japan; catalog no. 340) and propylene oxide (1:1 ratio). Following this, the cells were embedded in Epon Quetol-812 and solidified for 2 days at 60°C. The Epon-embedded samples were used to prepare ultrathin sections using an ultramicrotome (Leica Microsystems, Hesse, Germany; catalog no. Leica EM UC6). In fig. S3B, sections mounted on a copper grid coated with 0.5% formvar (Nisshin EM Co. Ltd.; catalog no. 604) were imaged using an electron microscope (JEOL Ltd., Tokyo, Japan; catalog no. JEM-1400Flash). In Figs. 1G and 2B and fig. S6, sections mounted on a cleaned silicon wafer strip (Canosis Co. Ltd., Tokyo, Japan; catalog no. SiD-2) were imaged with a different electron microscope (JEOL Ltd., Tokyo, Japan; catalog no. JSM-7900F).

### Freeze-fracture electron microscopy

For FF, cells were seeded onto a 6-cm dish at a density of 0.89 × 10^5^ cells/cm^2^ and cultured for ~14 days at 37°C with 5% CO_2_. Subsequently, they were fixed with the fixative solution containing 2.5% (v/v) EM-grade glutaraldehyde, 2.0% (w/v) paraformaldehyde, and 100 mM Hepes (pH 7.4) for 1 hour at room temperature. After three washes with 100 mM Hepes (pH 7.4) buffer for 10 min each, cell sheets were detached using a rubber scraper and then immersed in a solution containing 35% (v/v) glycerol and 100 mM Hepes (pH 7.4) overnight at 4°C. Subsequently, the cell sheets were rapidly frozen in a nitrogen slush using a nitrogen slusher machine (JEOL Ltd.; catalog no. EM-19510SNPD). To obtain replicas, the frozen cell sheets were fractured at −120°C and then coated with platinum-carbon (JEOL Ltd.; catalog no. 781121370) at a 45° angle for 20 s using a voltage of 1.8 kV and an emission current of 80 mA using a freeze-fracture equipment (JEOL Ltd.; catalog no. EM19500-NFSDT). Carbon (JEOL Ltd.; catalog no. 781121361) was then added at a 90° flat angle for 10 s, using a voltage of 2.2 kV and an emission current of 90 mA with the same freeze-fracture equipment. The replica samples were immersed in household bleach for 2 hours at room temperature. Subsequently, the replicas were washed three times with Milli-Q water containing a 1000-fold dilution of a Photo-Flo 200 Solution (Eastman Kodak Company, Rochester, NY, USA; catalog no. 146 4510). The replica was mounted on a copper grid coated with a 0.5% formvar solution (Nisshin EM Co. Ltd.; catalog no. 604), and images of the replica were captured using an electron microscope (JEOL Ltd.; catalog no. JEM-1400 plus) at a magnification of ×20,000. For morphometric analyses, TJ strand abundance was determined by manually enumerating the intersections between TJ strands and a line drawn along the axis from the apical to basal side at intervals of 200 nm. The complexity of TJ strands was assessed by manually enumerating branch points per unit length (1 μm) along the TJ strands.

For FF of a mouse stomach, the stomach was incised along its greater curvature. The corpus of the stomach was cut into pieces of ~3 mm by 3 mm and directly immersed in the fixative solution as described above for 1 hour at room temperature with agitation. After three washes with 100 mM Hepes (pH 7.4) buffer for 10 min each, the specimens were immersed in a solution containing 20% (v/v) glycerol and 20 mM Hepes (pH 7.4) for 3 hours at 4°C. The procedures for specimen freezing, replica fabrication, and replica image acquisition were similar to those described above.

### Cryo–focused ion beam milling

Titanium grids (100 mesh) (Ted Pella Inc., Redding, CA, USA; catalog no. 1GT100) and gold grids (150 mesh) (Nisshin EM Co. Ltd., Tokyo, Japan; catalog no. 2612-1) were placed on cover glass slides, coated with formvar-carbon, and sterilized under ultraviolet light. Cells were carefully plated on the prepared grids in a six-well plate at a density of 0.89 × 10^5^ cells/cm^2^ to ensure that cells attached to only one side of the grids. The medium was changed daily for 7 to 9 days until the cell sheets matured after they covered the whole grid. Before the freezing procedure, in some cases, 10% (v/v) dimethyl sulfoxide (DMSO) was added to the well for 5 min to aid vitrification. Sample grids were taken from cell culture wells and mounted in a Vitrobot Mark IV (Thermo Fisher Scientific). The sample chamber was kept at 37°C and 80% humidity, and grids were blotted for 10 s with filter papers on both sides before being plunged into a liquid ethane-propane mixture. In some cases, after 5 s of blotting, 0.1% (v/v) Triton X-100 was applied to the grids before another 10 s of blotting. Grids were stored under liquid nitrogen until use.

Grids were mounted in autogrids modified with a cutout on one side for better access for the ion beam (*64*). All cryo–focused ion beam milling procedures were done using an Aquilos 2 dual-beam instrument (Thermo Fisher Scientific). Before milling, samples were sputter coated with Pt for 15 s at 30 mA and coated with a layer of organometallic Pt for 10 s using the integrated gas injection system. After target identification, 12 mm–by–15 mm trenches above and below the target area were milled manually. Automated rough milling was set up using Maps and AutoTEM (Thermo Fisher Scientific), and milling currents were reduced stepwise from 0.5 to 0.1 nA. Microexpansion joints (*65*) were used for stress relief. Final lamella thinning from ~250 to <200 nm was done manually using an ion beam current of 50 or 30 pA. Samples were removed from the instrument and stored under liquid nitrogen.

### Cryo–electron tomography

A Thermo Fisher Scientific Krios G4 transmission electron microscope operated at 300 kV and equipped with a BioQuantum energy filter and a K3 direct electron detector (Gatan, Pleasanton, CA, USA) was used. Tilt series with a range of 120° and 3° increments were acquired dose-symmetrically (*66*) in parallel using PACEtomo (*67*) within SerialEM (*68*, *69*). After the alignment of movie frames using alignframes in IMOD (*70*, *71*), tilt series were aligned by patch tracking and reconstructed using back projection using IMOD. Tomograms were denoised for visualization using cryo-CARE (*72*).

### TEER measurement

Cells were plated onto the semipermeable PET membrane filter with 0.4-μm pores and a growth area of 0.33 cm^2^ at a density of 0.89 × 10^5^ cells/cm^2^ and maintained at 37°C with 5% CO_2_. The culture medium on both the apical and basal sides of the filter was replaced daily. The TEER was measured daily in the culture medium for ~15 days using a Millicell ERS-2 instrument (Merck; catalog no. MERS00002). TEER, expressed as ohms × square centimeters, was calculated by subtracting the background value obtained from a blank filter and then multiplying it by 0.33 cm^2^ (the growth area of the filter).

### Electrical conductance and DP measurement

Electrophysiological analyses of cultured cells were performed as described previously (*29*). Cells were cultured on a semipermeable PET membrane filter featuring 0.4-μm pores and providing a growth area of 1.13 cm^2^. The cells were seeded at a density of 0.89 × 10^5^ cells/cm^2^ and cultured for ~14 days at 37°C with 5% CO_2_. The cell layer on the filter was placed into a Ussing chamber and prewarmed to 37°C, with a pore diameter of 5 mm. Both the apical and basal sides of the chamber were filled with 5 ml of solution, containing 150 mM NaCl, 2 mM CaCl_2_, 1 mM MgCl_2_, 10 mM mannitol, and 10 mM tris-Hepes at pH 7.4, and prewarmed to 37°C. The transepithelial voltage of the cell layer was measured using voltage amplifiers (Nihon Kohden Corporation, Tokyo, Japan; catalog no. CEZ-9100) through 3 M KCl agar bridges connected to calomel electrodes. The transepithelial electrical conductance of the cell layer was calculated according to Ohm’s law by passing current and measuring the potential change across the cell layer. The current was applied through a pair of Ag/AgCl electrodes placed in contact with the bathing solutions via a pair of 1 M NaCl agar bridges. Before the experiments, both the background fluid resistance and the potential difference of the system were adjusted to zero using a blank filter.

To determine the NaCl DP, the basal side of the solution was substituted with a solution containing 75 mM NaCl instead of the original 150 mM NaCl (maintaining osmolarity with mannitol). The voltage electrodes were placed to show cation selectivity as a positive value. Subsequently, the resulting DP of the cell layer was adjusted to account for that of the blanked filter. Throughout the analysis, the Ussing chamber and solution were maintained at 37°C.

### LaCl_3_ treatment for cultured cells

For the investigation of LaCl_3_-induced changes in TEER of cultured cells, cells were seeded onto a semipermeable PET membrane filter with 0.4-μm pores and a growth area of 0.33 cm^2^ at a density of 0.89 × 10^5^ cells/cm^2^. The cells were cultured for ~14 days at 37°C with 5% CO_2_. Both the apical and basal sides of the filter were replaced with Ringer solution [140 mM NaCl, 2 mM CaCl_2_, 1 mM MgCl_2_, 10 mM glucose, 20 mM mannitol, and 10 mM tris-Hepes (pH 7.4)] supplemented with 5 mM LaCl_3_. For control experiments, Ringer solution without LaCl_3_ was used. TEER of the cell sheets was measured at 0, 5, 10, and 15 min after the medium replacement.

### Electrostatic surface potential of Cldn structures

Structural models of mouse Cldns were obtained from the AlphaFold Protein Structure Database (*73*). The surface distribution of the electrostatic potential was calculated using the Adaptive Poisson-Boltzmann Solver (*74*). All structural figures were prepared using PyMOL (www.pymol.org/).

### Classification of Cldn family members

For the classification of Cldn family members, transepithelial electrical conductance, DP, and TJ strand numbers for single *Cldn*-expressing cells were standardized into *z*-scores using the “scales” package in R. Using the DBSCAN algorithm (*75*) in the “dbscan” package in R (*76*), members were initially classified on the basis of two indexes, electrical conductance and the number of TJ strands, resulting in four main classes (classes 1 to 4). Subsequently, DP was incorporated to further subclassify the members in classes 1 and 2. The DBSCAN parameter “minPts” was set to 3, and “epsilon” was determined from *k*-nn distance plots using the kNNdist function in R.

### pH measurement for mouse stomachs

A mouse stomach treated with histamine, omeprazole, or DMSO was incised along its greater curvature. Following the removal of luminal contents, a voltage-sensing electrode was gently placed in contact with the lumen of the stomach corpus to measure the voltage using a pH meter (HORIBA Ltd., Kyoto, Japan; catalog no. F-71) acting as a voltmeter. Voltages of solutions with known pH values of 4.01, 6.86, and 9.18 were measured as standards.

### Acid loading for mouse stomachs

To acidify the luminal pH of the mouse stomach, histamine (FUJIFILM Wako Pure Chemical Corporation; catalog no. 081-03551) dissolved in 10% (v/v) DMSO (Nacalai Tesque; catalog no.08904-14) containing 150 mM NaCl was intraperitoneally injected into each mouse at a dose of 10 mg/kg of body weight 12 and 3 hours before euthanasia. For neutralization of the lumen of the mouse stomach, omeprazole (FUJIFILM Wako Pure Chemical Corporation; catalog no. 158-03491) dissolved in the same solution was injected into each mouse at a dose of 60 mg/kg of body weight using the same procedure, 12 and 3 hours before euthanasia. Mice were injected with a 10% (v/v) DMSO solution containing 150 mM NaCl as a control experiment.

To measure acid-induced changes in electrical conductance, a mouse stomach treated with histamine, omeprazole, or DMSO was incised along its greater curvature. The stomach lumen was gently rinsed with an ice-cold solution comprising 150 mM NaCl, 2 mM CaCl_2_, 1 mM MgCl_2_, 10 mM mannitol, and 10 mM tris-Hepes at pH 7.4. Subsequently, the corpus of the gastric mucosa was mounted onto filter paper soaked with the solution and inserted into a Ussing chamber with a pore size of 5 mm in diameter. Both the luminal and submucosal sides of the chamber were filled with a solution containing 150 mM NaCl, 2 mM CaCl_2_, 1 mM MgCl_2_, 10 mM mannitol, and 10 mM tris-Hepes at pH 7.4, supplemented with 10% (v/v) FBS. Before experiments, both the background fluid resistance and potential difference of the system were nullified and adjusted to zero using the blank filter paper. Subsequently, the solution on the luminal side was replaced sequentially with the solutions at pH 7.0, 6.0, 5.0, and 4.0. After each pH change, transmucosal electrical conductance of the gastric mucosa was measured for 5 min at 1-min intervals.

For an investigation into acid-induced alterations in the distribution of cell-cell adhesion proteins through IF, a mouse stomach treated with histamine, omeprazole, or DMSO was incised along its greater curvature. Subsequently, it was directly embedded in Tissue-Tek O.C.T. Compound and rapidly frozen using liquid nitrogen. The frozen stomach was then subjected to IF.

For FF of acid-induced changes in TJ strand formation in the stomach, the corpus of the histamine-, omeprazole-, or DMSO-treated stomach was cut into pieces of ~3 mm by 3 mm. These sections were then directly immersed in the fixative solution, as described previously, for 1 hour at room temperature with agitation. Subsequently, the fixed stomach sections underwent FF using the previously outlined protocol.

### Acid loading for cultured cells

For an investigation into acid-induced changes in TEER of cultured cells, cells were seeded onto a semipermeable PET membrane filter with 0.4-μm pores and a growth area of 0.33 cm^2^ at a density of 0.89 × 10^5^ cells/cm^2^. The cells were cultured for 21 days at 37°C with 5% CO_2_. To induce acid loading, the medium on the apical side of the filter was replaced with DMEM adjusted to pH 5.0 using HCl, supplemented with 10% (v/v) FBS, 2 mM l-glutamine, and 0.45% (w/v) d(+)-glucose. For control experiments, the medium on the apical side of the filter was replaced with pH 7.0 medium. TEER of the cell sheets was measured at 0, 5, 20, 49, and 60 min after the medium change.

To measure acid-induced changes in the electrical conductance of cultured cells, cells were seeded onto the semipermeable PET membrane filter with 0.4-μm pores and a growth area of 1.13 cm^2^ at a density of 0.89 × 10^5^ cells/cm^2^. The cells were cultured for 21 days at 37°C with 5% CO_2_. The cell layer on the filter was placed into a Ussing chamber and prewarmed to 37°C, with a pore diameter of 5 mm. Both the apical and basal sides of the chamber were filled with 5 ml of solution containing 150 mM NaCl, 2 mM CaCl_2_, 1 mM MgCl_2_, 10 mM mannitol, and 10 mM tris-Hepes at pH 7.4 supplemented with 10% (v/v) FBS. Subsequently, the solution on the apical side was replaced sequentially with solutions at pH 7.0, 6.5, 6.0, 5.5, 5.0, 4.5, 4.0, 3.5, and 3.0. After each pH change, transepithelial electrical conductance of the cell layer was measured for 5 min at 1-min intervals. In another set of experiments, the DP was assessed following the measurement of electrical conductance. This was accomplished by replacing the basal side solution in the chamber with a solution containing 75 mM NaCl, 2 mM CaCl_2_, 1 mM MgCl_2_, 10 mM mannitol, and 10 mM tris-Hepes at pH 7.4, supplemented with 10% (v/v) FBS. The luminal side was maintained at pH 7.4 or 3.0.

To investigate acid-induced changes in the distribution of cell-cell adhesion proteins via IF, cells were seeded onto the semipermeable PET membrane filter with 0.4-μm pores and a growth area of 1.13 cm^2^ at a density of 0.89 × 10^5^ cells/cm^2^. The cells were cultured for 21 days at 37°C with 5% CO_2_. Thirty minutes after acid loading, performed as described above, the cells were fixed with ice-cold methanol for 10 min at −20°C. The fixed cells were evaluated using IF as described above.

For FF of acid-induced changes in TJ strand formation in cultured cells, cells were seeded onto a 6-cm dish at a density of 0.89 × 10^5^ cells/cm^2^ and cultured for 21 days at 37°C with 5% CO_2_. Thirty minutes after acid loading, the cells were fixed with a solution containing 2.5% (v/v) EM-grade glutaraldehyde, 2.0% (w/v) paraformaldehyde, and 100 mM Hepes (pH 7.4) for 1 hour at room temperature. The fixed cells underwent FF as described above.

### Quantification and statistical analysis

Statistical analysis was performed using Excel (Microsoft, Redmond, WA, USA), R version 4.3.3 (the R Foundation, RRID: SCR_001905), and RStudio (RStudio, PBC, RRID: SCR_000432). The normality of the data distribution was tested using the Shapiro-Wilk test, and either a parametric or nonparametric statistical method was selected. Homogeneity of variances was tested using the *F* test when comparing two groups or the Bartlett test when comparing more than two groups. For parametric analysis between two groups, Student’s *t* test was used when the assumption of homogeneity of variance was met, and Welch’s *t* test was used when it was not. For parametric analysis between more than two groups, Fisher’s one-way analysis of variance (ANOVA) with the Tukey-Kramer multiple-comparison test was used when the assumption of homogeneity of variance was satisfied. When the assumption of homogeneity of variances was not satisfied, Welch’s one-way ANOVA with the Games-Howell test was used alternatively. For nonparametric analyses, between two groups, the Mann-Whitney *U* test was used when the assumption of homogeneity of variance was met, and otherwise, the Brunner-Munzel test was used. For nonparametric analysis between more than two groups, Kruskal-Wallis tests with pairwise-comparison tests or Dunn’s test were used. For a many-to-one comparison, Steel’s multiple-comparison test was used. Data are presented as means ± SDs. Results with *P* values less than 0.05 were considered statistically significant.

## Supplementary Materials

### The PDF file includes:

Figs. S1 to S11 Table S1 Legends for movies S1 to S5 References
