# Supplementary material for: Functional landscape of mechanistic diversity in 27 claudin family members at tight junctions
Source: Sci Adv. 2025 Oct 31;11(44):eadx7431. doi: 10.1126/sciadv.adx7431 (PMC12577684; doi:10.1126/sciadv.adx7431)
Supplement: Supplementary file 1 — Figs. S1 to S11 Table S1 Legends for movies S1 to S5 References [file sciadv.adx7431_sm.pdf]

Science Advances
AAAS

Supplementary Materials for

Functional landscape of mechanistic diversity in 27 claudin family members at tight junctions

Hiroka Kashihara *et al.*

Corresponding author: Sachiko Tsukita, atsukita@med.teikyo-u.ac.jp, stsukitatjcl@gmail.com;
Atsushi Tamura, atamura@med.teikyo-u.ac.jp; Hiroo Tanaka, htanaka994@gmail.com

Sci. Adv. **11**, eadx7431 (2025)
DOI: 10.1126/sciadv.adx7431

The PDF file includes:

Figs. S1 to S11
Table S1
Legends for movies S1 to S5
References

Other Supplementary Material for this manuscript includes the following:

Movies S1 to S5

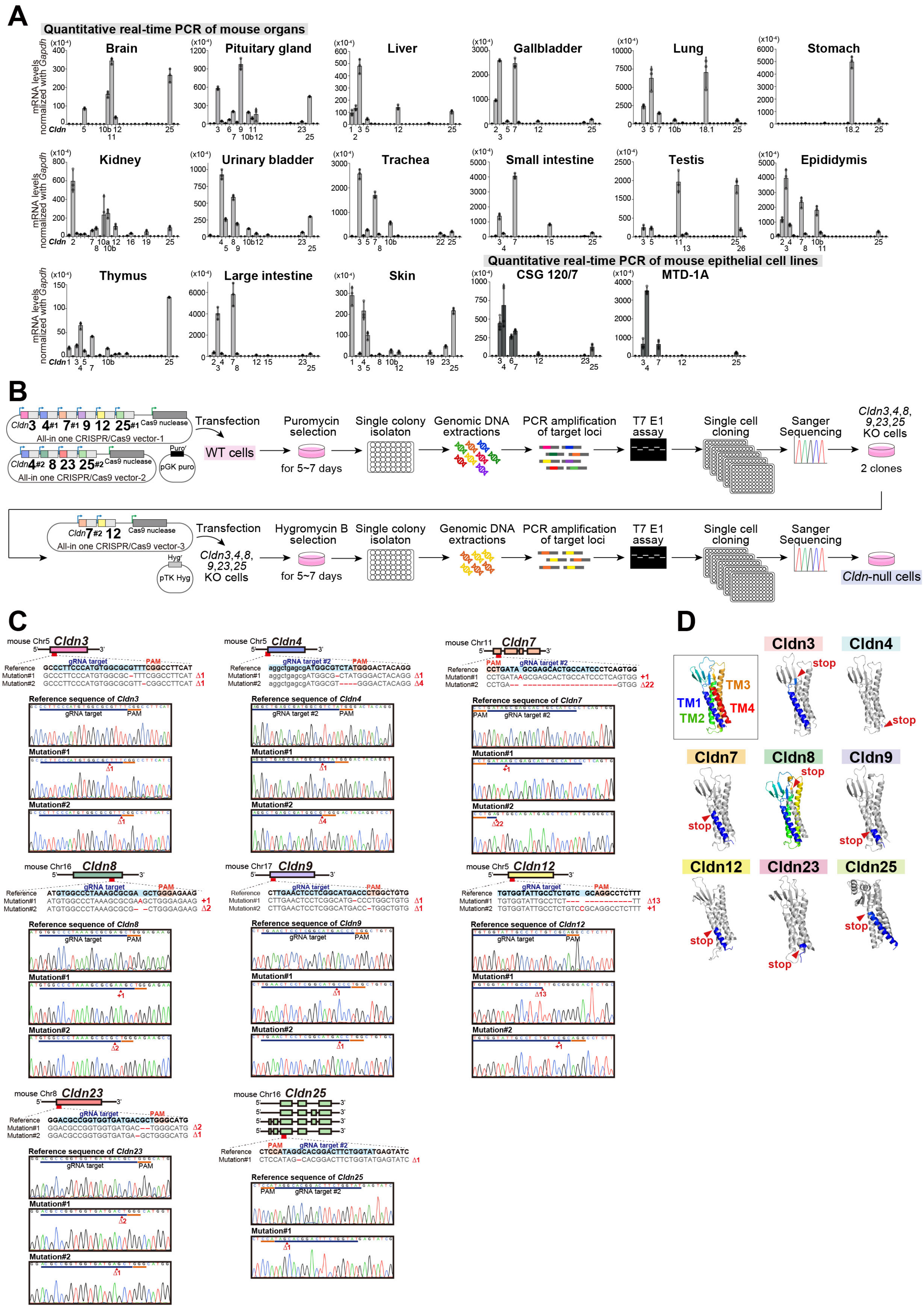
A
Quantitative real-time PCR of mouse organs
mRNA levels normalized with Gapdh
Brain
Pituitary gland
Liver
Gallbladder
Lung
Stomach
Kidney
Urinary bladder
Trachea
Small intestine
Testis
Epididymis
Thymus
Large intestine
Skin
Quantitative real-time PCR of mouse epithelial cell lines
CSG 120/7
MTD-1A
B
All-in one CRISPR/Cas9 vector-1
All-in one CRISPR/Cas9 vector-2
All-in one CRISPR/Cas9 vector-3
Cas9 nuclease
pGK puro
pTK Hyg
Transfection
WT cells
Puromycin selection
for 5~7 days
Single colony isolaton
Genomic DNA extractions
PCR amplification of target loci
T7 E1 assay
Single cell cloning
Sanger Sequencing
Cldn3,4,8,9,23,25 KO cells
2 clones
Hygromycin B selection
Cldn-null cells
C
Reference
Mutation#1
Mutation#2
gRNA target
PAM
Reference sequence of Cldn3
Reference sequence of Cldn4
Reference sequence of Cldn7
Reference sequence of Cldn8
Reference sequence of Cldn9
Reference sequence of Cldn12
Reference sequence of Cldn23
Reference sequence of Cldn25
D
Cldn3
Cldn4
Cldn7
Cldn8
Cldn9
Cldn12
Cldn23
Cldn25
TM1
TM2
TM3
TM4
stop

Fig. S1. Generation of *Cldn*-null epithelial cells. Related to Fig. 1.

(**A**) mRNA expressions of *Cldns* in WT mouse organs (n = 3 mice) and mouse cultured epithelial cell lines (n = 3 samples). mRNA levels were examined by quantitative real-time PCR (qPCR) and normalized to *Gapdh*, Values are means ± SD. The expression profiles of mouse organs are summarized in Fig. 1A.

(**B**) Generation of *Cldn*-null cells using all-in-one CRISPR/Cas9 vectors for multiplex genome editing of *Cldn3*, *4*, *7*, *8*, *9*, *12*, *23*, and *25*.

(**C**) Genomic DNA sequencing of *Cldn*-null cells revealing frameshift insertion/deletion (indel) mutations in the coding regions of *Cldn3*, *4*, *7*, *8*, *9*, *12*, *23*, and *25* (colored boxes), compared with WT cells. Indel mutations are indicated by the number of inserted (+) or deleted (Δ) bases. Chr, chromosome.

(**D**) Truncations of the Cldn3, 4, 7, 8, 9, 12, 23, and 25 proteins due to frameshift indel mutations in *Cldn*-null cells. Structures of mouse Cldn15 (crystal structure) as well as Cldn3, 4, 7, 8, 9, 23, and 25 (AlphaFold Protein Structure Database) showing similarities, including four transmembrane regions (TM1–4) and an extracellular β-sheet domain (box).

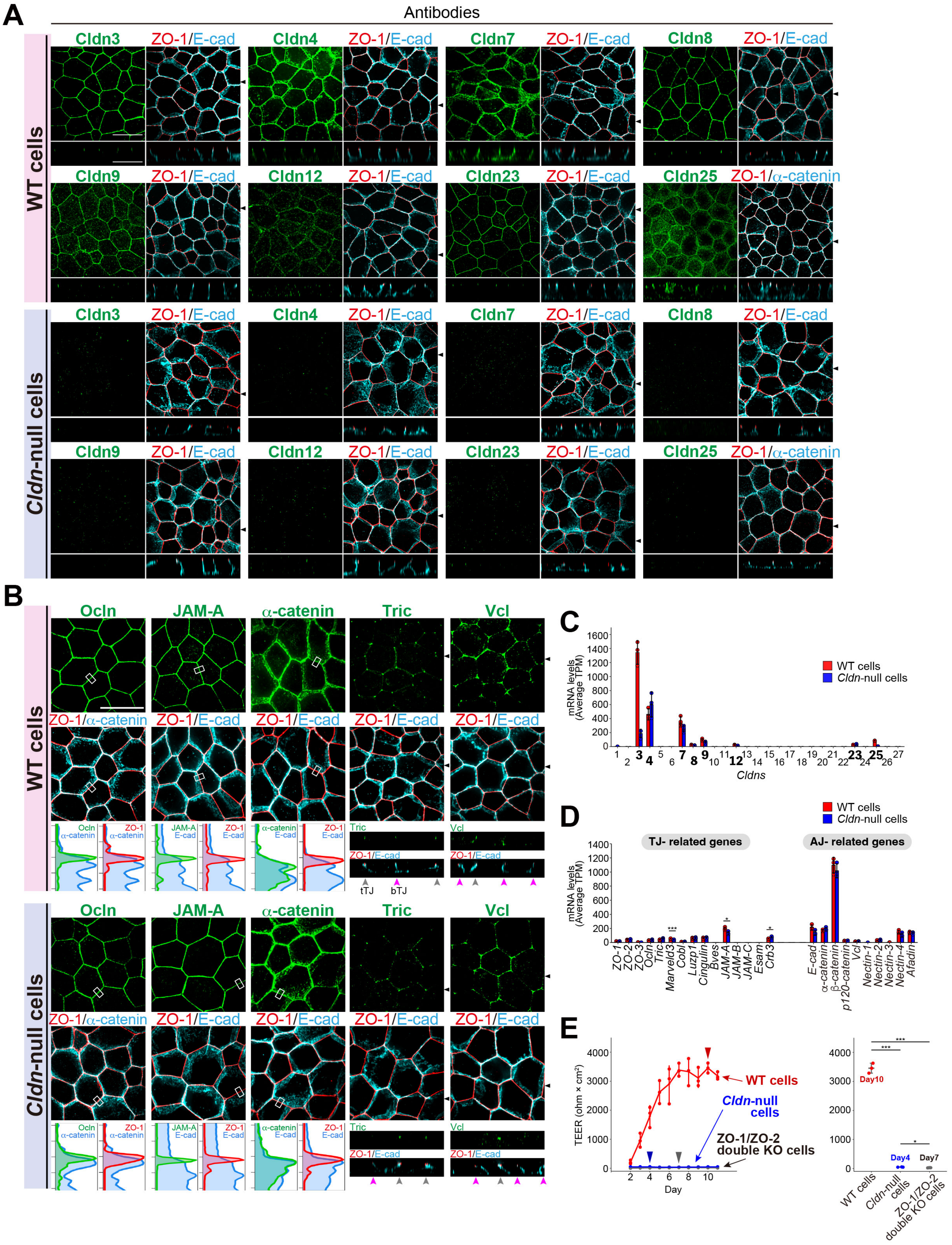

Fig. S2. Please refer to the next page for the captions.

Fig. S2. Super-resolution immunofluorescence microscopy (IF) of TJ-/AJ-proteins and mRNA expression profiles of TJ-/AJ-related genes in *Cldn*-null cells. Related to Fig. 1.

(**A**) Super-resolution immunofluorescence microscopy (IF) of WT and *Cldn*-null cells co-stained for Cldn3, 4, 7, 8, 9, 12, 23, or 25, with ZO-1 and either E-cadherin (E-cad) or α-catenin. Z-stack images (upper) and orthogonal views (lower) at arrowhead positions.

(**B**) IF of TJ proteins (occludin [Ocln], JAM-A, and tricellulin [Tric]) and adherens junction (AJ) proteins (α-catenin and vinculin [Vcl]) in WT and *Cldn*-null cells, co-stained with ZO-1 and either E-cad or α-catenin. Z-stack images (upper panels), and intensity plots for the rectangular areas or orthogonal views at arrowhead positions (lower panels) are shown. Tric and Vcl are more abundant in tricellular TJ (tTJ) than bicellular TJ (bTJ), as indicated by arrowheads, in both WT and *Cldn*-null cells.

(**C, D**) RNA-seq of *Cldns* (C) and TJ-/AJ-related genes (D) in WT and *Cldn*-null cells. mRNA levels are shown as transcripts per million (TPM, n = 3 libraries, respectively). The same data were used in Fig. 1B. Values are means ± SDs. ***$p < 0.001$, *$p < 0.05$, n.s.: not significant. Student's t-test, Welch's t-test, Mann-Whitney U test, or Brunner-Munzel test was used, depending on the normality of the data distribution and the homogeneity of variances.

(**E**) Trans-epithelial electrical resistance (TEER) of WT, *Cldn*-null, and ZO-1/ZO-2 double KO cells (n = 3 wells, respectively). Time courses of the TEER values from day 2 to day 11 (left) and the peak TEER values (right) are shown. Arrowheads indicate the individual peaks. The same data were used in Fig. 1H. Values are means ± SDs. ***p < 0.001, *$p < 0.05$ (Welch's one-way ANOVA with Games-Howell test).

Scale bars: 10μm.

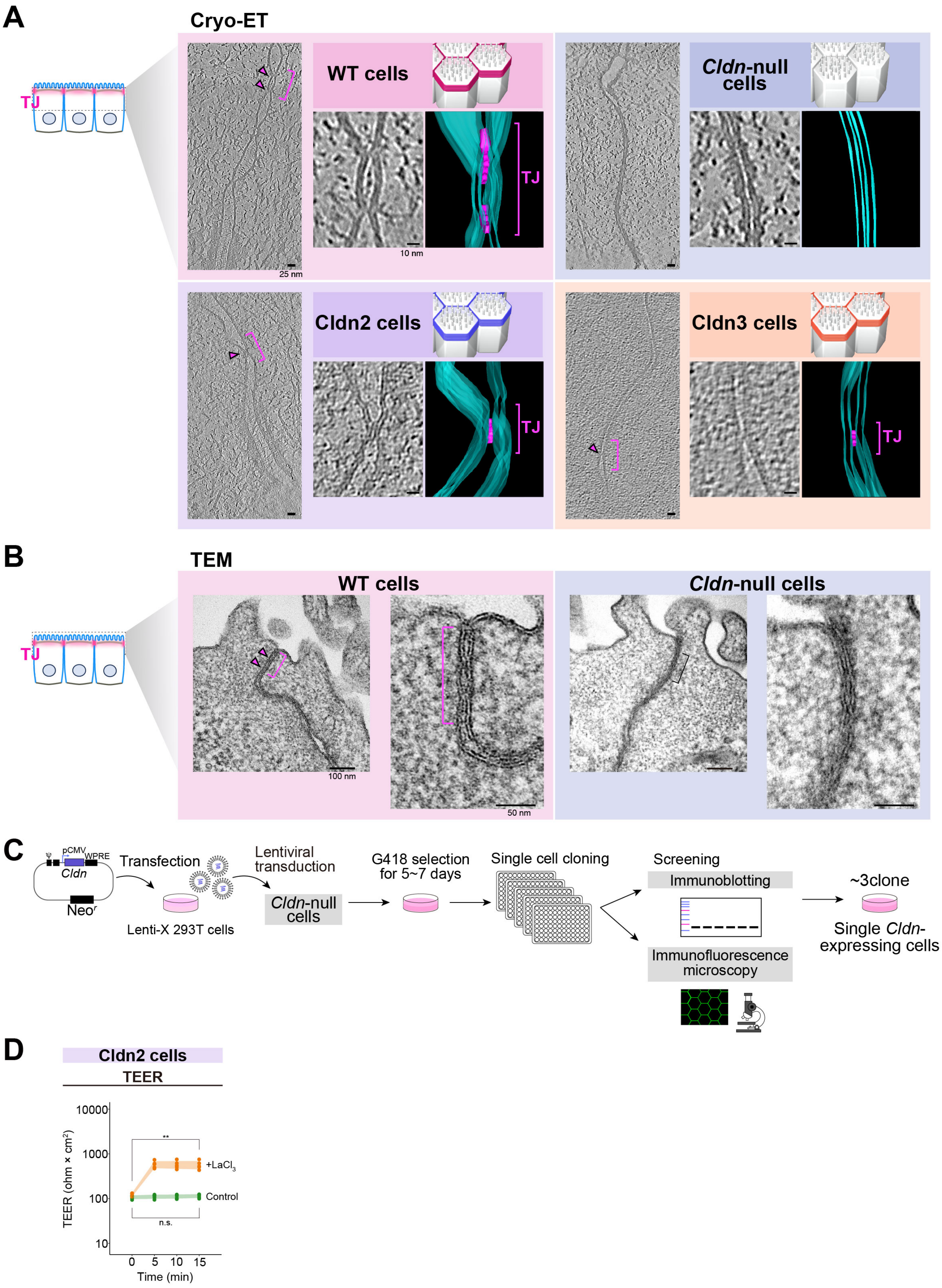

Fig. S3. Please refer to the next page for the captions.

Fig. S3. Generation of single *Cldn*-expressing epithelial cells. Related to Figs. 1 and 2.

(**A**) Cryo-electron tomography (Cryo-ET) of WT, *Cldn*-null cells, and single *Cldn2* or Cldn3-expressing cells (Cldn2 cells or Cldn3 cells). Arrowheads indicate kissing points of TJs. In Cryo-ET, tomographic slices are displayed, with 3D reconstructions from the most apical region of the lateral membranes shown as false-colored images. These reconstructions highlight the intercellular regions of TJs (magenta) and lipid bilayers (cyan). Low magnification (left panels) and high magnification (right panels), respectively. Scale bars: 25 nm (low magnification), 10 nm (high magnification). The same data were used in Figs. 1G and 2C.

(**B**) Thin-section electron microscopy (TEM) of WT and *Cldn*-null cells. Low magnification (left panels) and high magnification (right panels). Arrowheads indicate kissing points of TJs. Scale bars: 100 nm (low magnification), 50 nm (high magnification).

(**C**) Procedures for generating single *Cldn*-expressing cells for all *Cldn* family members (Cldn1–27 cells, respectively) through lentivirus-mediated gene transduction into *Cldn*-null cells. Following screening by immunoblotting and IF, two or three distinct cell clones were established for each member.

(**D**) Time-courses of the TEER values in Cldn2 cells from 0 to 15 minutess after $LaCl_3$ tratment are shown (n = 5 wells from control or $LaCl_3$-treated Cldn2 cells, repectively). To asses time-dependent changes in TEER values, Fisher's one-way ANOVA with the Tukey–Kramer multiple-comparison test (control) and Welch's one-way ANOVA with the Games–Howell test (LaCl3) were used. Values are means ± SDs (error bands). $^{**}p < 0.001$, n.s.: not significant.

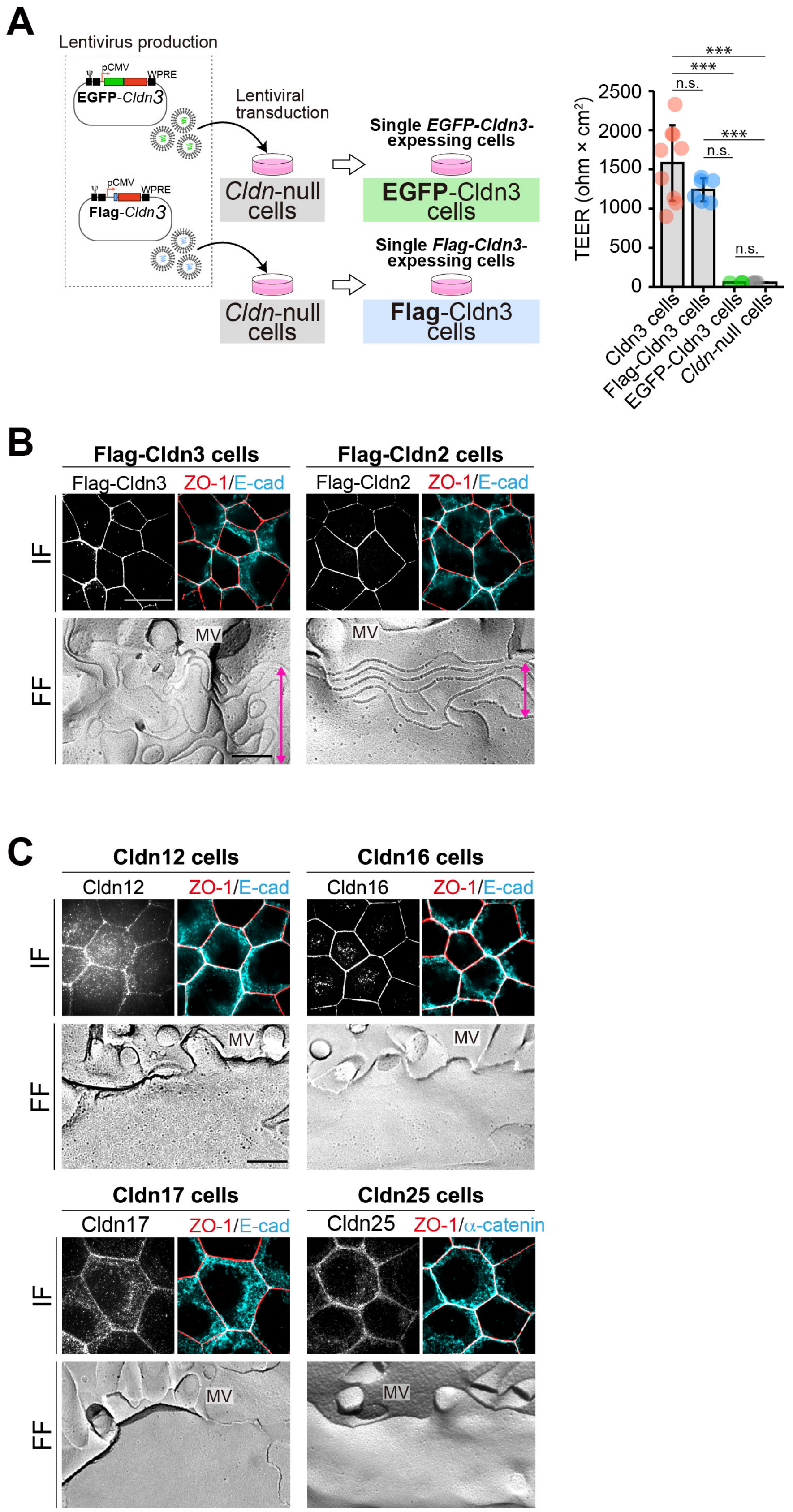

Fig. S4. Please refer to the next page for the captions.

Fig. S4. Infuluence of Flag-tag on TJ strand formation. Related to Fig. 3.

(**A**) TEER of Cldn3, Flag-Cldn3, and EGFP-Cldn3 cells as well as *Cldn*-null cells (n =3–9 wells, respectively). Values are means ± SDs. ***p < 0.001, n.s.: not significant (Welch's one-way ANOVA with the Games–Howell test).

(**B**) IF and FF of Flag-Cldn3 and Flag-Cldn2 cells. IF was performed using the anti-Flag antibody, with co-staining for ZO-1 and E-cad. FF shows TJ strands (bidirectional arrows).

(**C**) IF and FF of Cldn12/16/17/25 cells. IF was performed using specific anti-Cldn antibodies, with co-staining for ZO-1 and either E-cad or α-catenin. FF shows no TJ strands.

Scale bars: 10 μm (IF), 200 nm (FF).

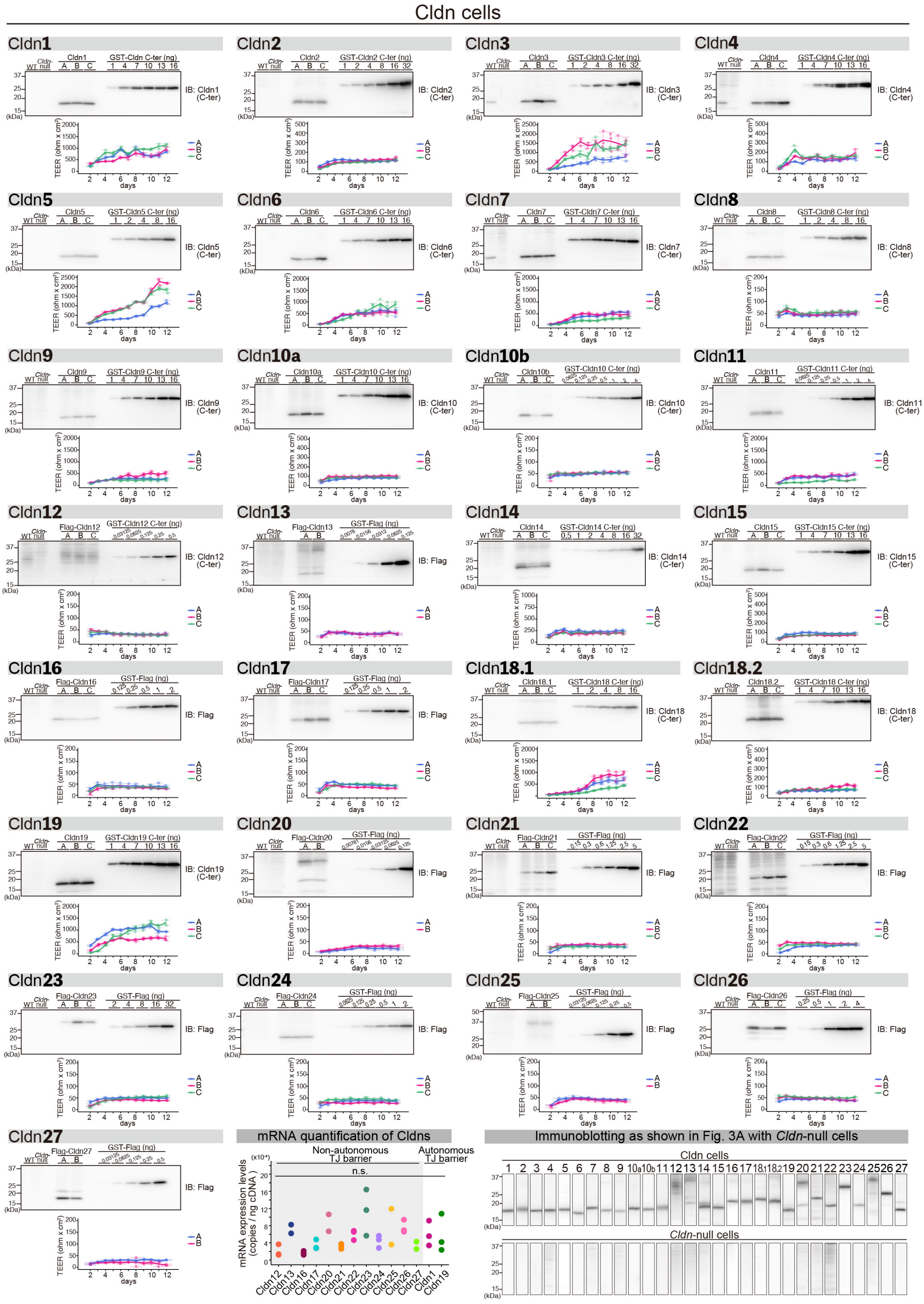

Fig. S5. Please refer to the next page for the captions.

Fig. S5. Protein expression levels of each Cldn in single *Cldn*-expressing cells. Related to Fig. 3.

Protein expression levels of individual Cldns in their respective Cldn cells (Cldn1–27 cells). Immunoblotting on Cldn1–27 cells (2–3 clones, labeled A–C) as well as WT and *Cldn*-null cells, using specific anti-Cldn antibodies for detecting Cldn1–12, 14, 15, 18.1, 18.2, and 19, along with the anti-Flag antibody for detecting other Cldns (Cldn13, 16, 17, and 20–27). For quantification, a dilution series of purified GST-Cldn C-terminal (C-ter) or GST-Flag proteins was utilized as a standard. These purified proteins were detected by specific anti-Cldn antibodies or the anti-Flag antibody. TEER values of Cldn1–27 cells during culture periods. qPCR of *Cldn1, 12, 13, 16, 17, 19, 20–27* in their respective Cldn cells (n = 2–3 clones, respectively). n.s.: not significant (Kruskal-Wallis test with Dunn's test).

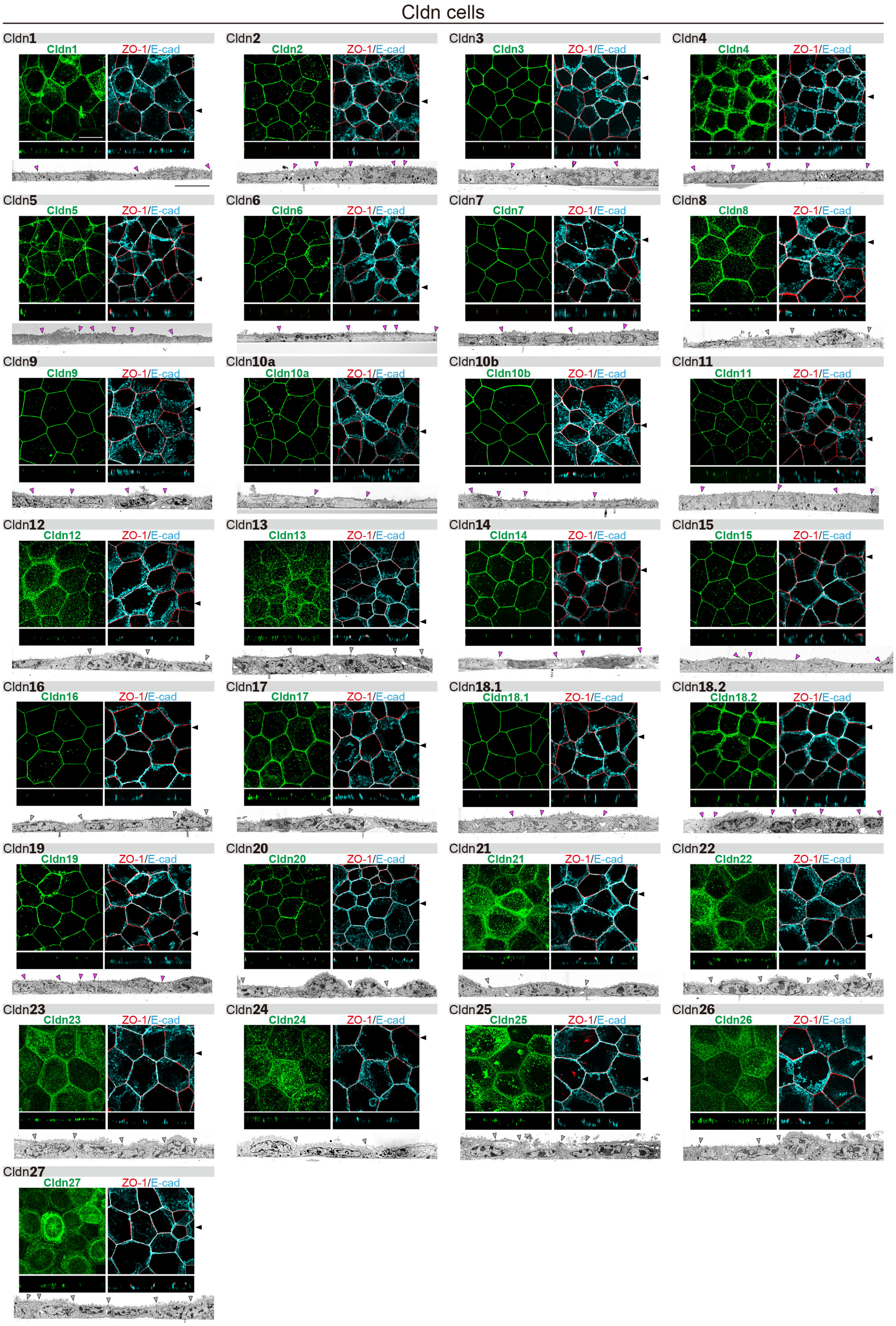

Fig. S6. Please refer to the next page for the captions.

Fig. S6. IF (low magnification) and TEM of single *Cldn*-expressing cells. Related to Fig. 3.

IF (low magnification) and TEM of Cldn1-27 cells. In IF, co-staining of individual Cldns in their respective cells with ZO-1 and E-cad is shown. Z-stack images (upper) and orthogonal views (lower) at arrowhead positions are shown. In TEM, magenta arrowheads denote TJ structures with kissing points, while gray arrowheads indicate structures resembling kissing points. Scale bars: 10 µm (IF), 10 µm (TEM).

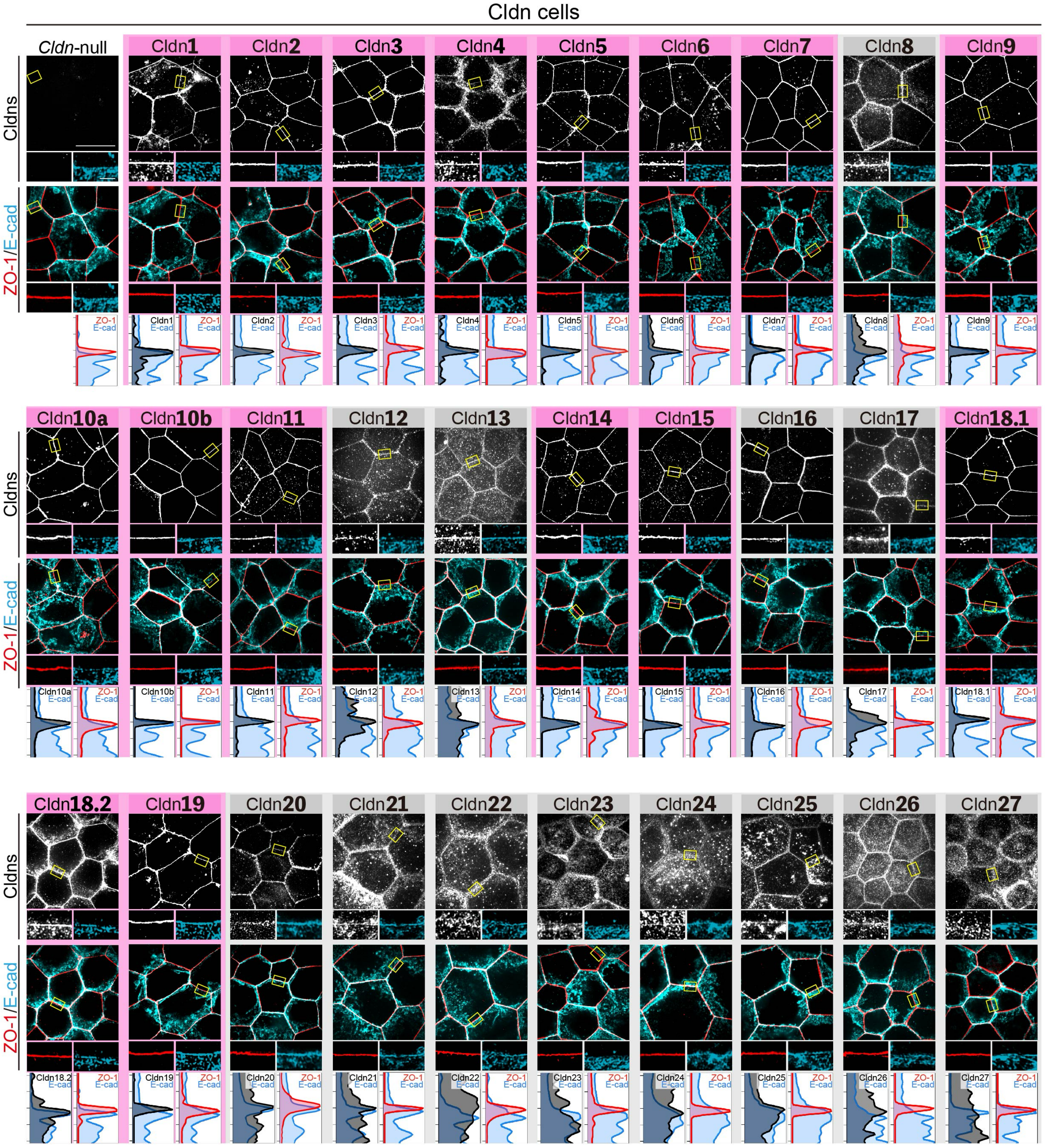

Fig. S7. Please refer to the next page for the captions.

Fig. S7. IF (high magnification) of single *Cldn*-expressing cells. Related to Fig. 3.

High-magnification images from IF of Cldn1–27 cells, using specific anti-Cldn antibodies for Cldn1–11, 14, 15, 18.1, 18.2, and 19, and the anti-Flag antibody for other Cldns (Cldn12, 13, 16, 17, and 20–27). Co-staining of individual Cldns with ZO-1 and E-cad in their respective cells, as shown in Fig. 3B, along with fluorescence plots for the rectangular areas. Co-stainning of Cldn3, ZO-1 and E-cad in *Cldn*-null cells is shown. Specific Cldn cells with or without TJ strands, as determined in Fig. 3B, are highlighted in pink and gray. Scale bars: 10 µm (low magnification), 1 µm (high magnification).

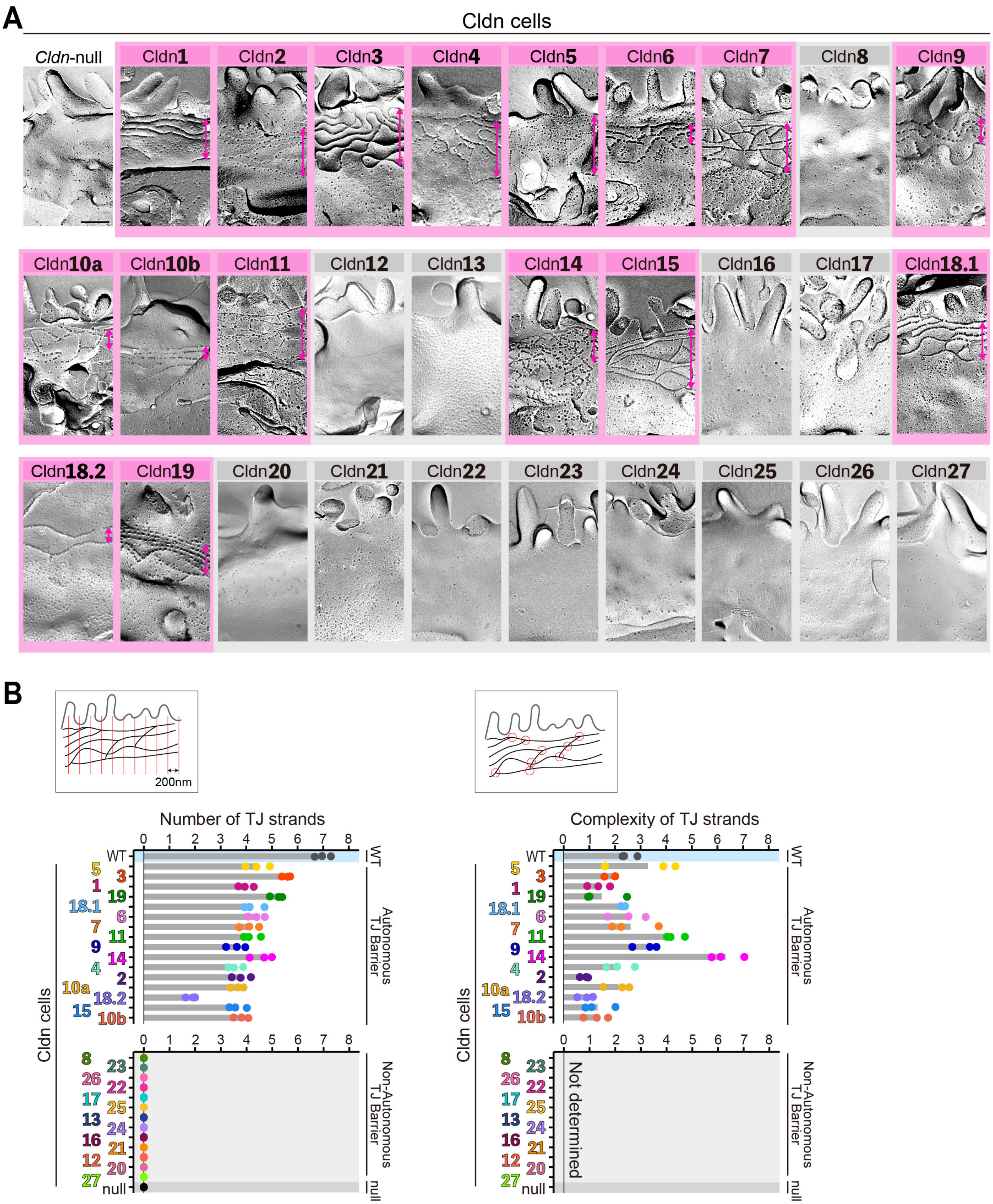

Fig. S8. Freeze-fracture electron microscopy (FF) of single *Cldn*-expressing cells. Related to Fig. 3.

(**A**) Freeze-fracture electron microscopy (FF) of Cldn1–27 cells. Wide fields images of Fig. 3B are shown. Scale bar: 200 nm.

(**B**) Morphometric analysis of FF images from Cldn1–27 cells in (A). As shown in the schematic images of TJ strands, the abundance of TJ strands (left) was quantified by manually enumerating the intersections between TJ strands and a line drawn along the axis from the apical to basal side at intervals of 200 nm. Each data point represents the average of 2–3 micrographs (total n = 4–9, respectively). The complexity of TJ strands (right) was assessed by manually enumerating branch points per unit length (1 μm) along the TJ strands (n = 2–3 micrographs, respectively).

Specific Cldn cells with or without TJ strands, as determined in Fig. 3B, are highlighted in pink and gray, respectively.

A

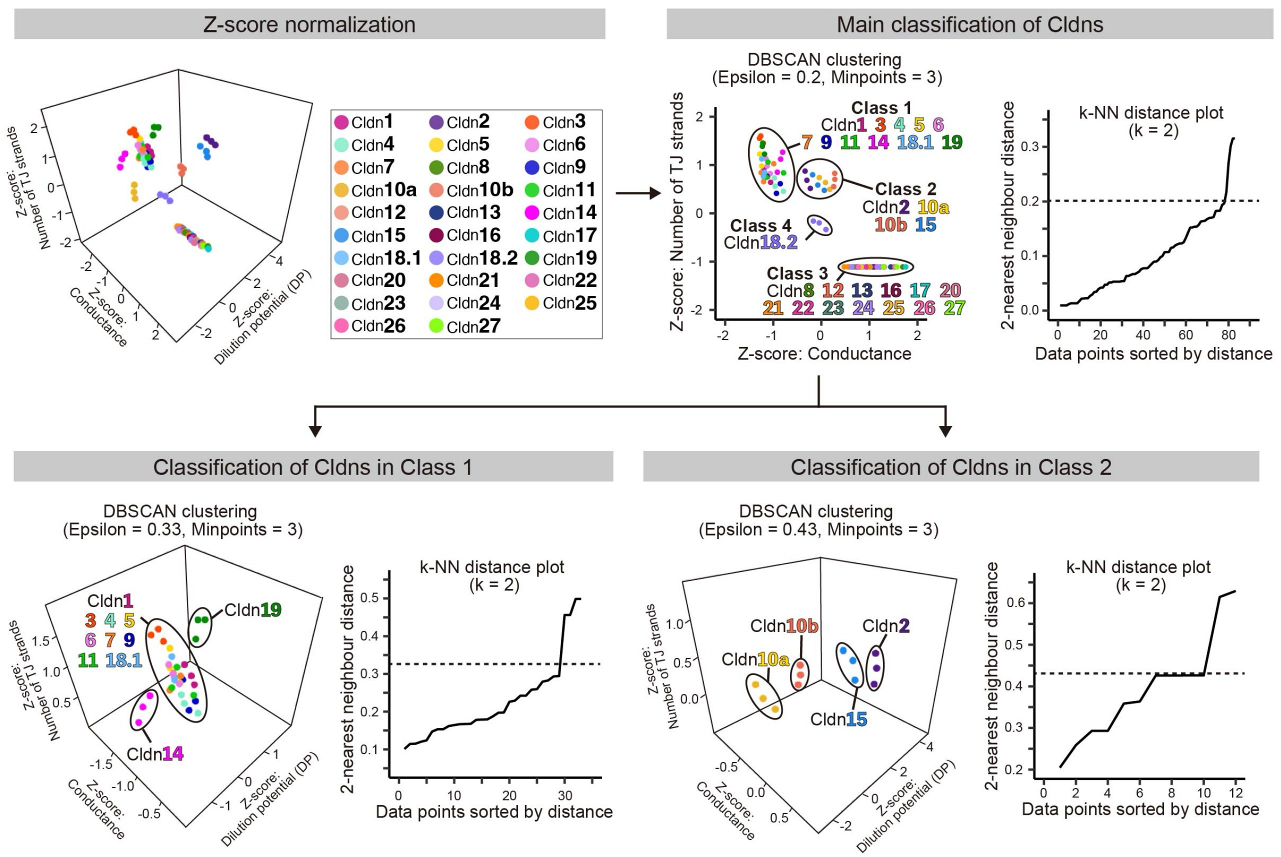

B

	This manuscript	Gonschior et al. Nat Commun. 2022	Previous accumulated reports
**Cell and/or organ type**	Single *Cldn*-expressing cell lines (Origin: *Cldn*-null epithelial cells)	Single *Cldn*-expressing cell lines (Origin: non-polarized epithelial like Cos-7 cells)	Various epithelial cell lines Various mouse organs
**Cldn expression**	Single Cldn	Single Cldn	Multiple Cldns
**Index**	• Electrical conductivity • Dilution potential (DP) • Number of TJ strands (FF)	• TJ-like meshwork patterns (STED)	• Electrical conductivity • Dilution potential
**Characterization**	27 *Cldn* family members	26 *Cldn* family members	20 *Cldn* family members
**Classification of Cldns**			

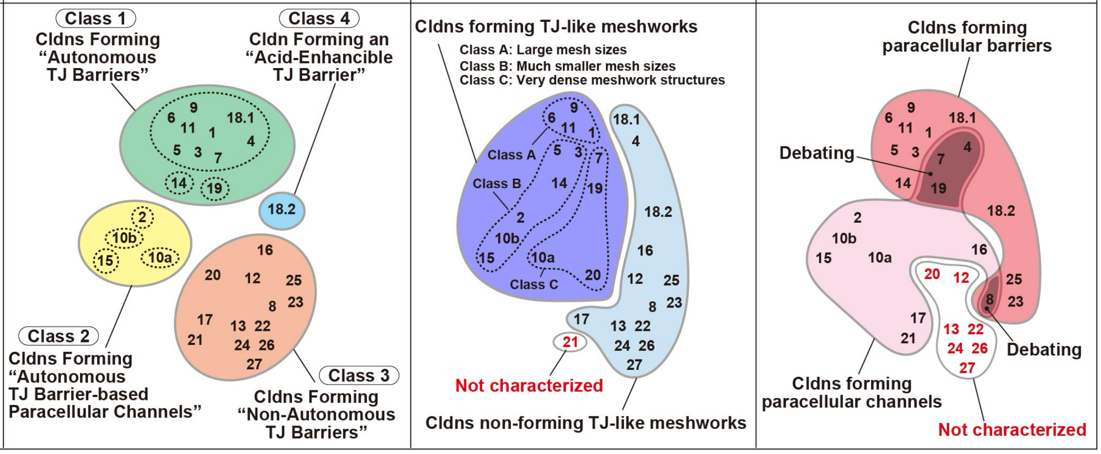

Fig. S9. Please refer to the next page for the captions.

Fig. S9. Classification of 27 Cldn family members. Related to Fig. 4.

(**A**) For the classification of Cldns, trans-epithelial electrical conductance, dilution potential (DP), and TJ strand numbers for each Cldn were standardized as a Z-score. Using the DBSCAN (Density-Based Spatial Clustering of Applications with Noise) algorithm, Cldns were initially classified based on two indexes: electrical conductance and the number of TJ strands, resulting in four main classes (Class 1–4). Subsequently, DP was incorporated to further sub-classify members in Class 1 and Class 2. The DBSCAN parameter minpoints was set to 3 and epsilon was determined using k-nearest neighbors (k-NN) distance plots.

(**B**) Classification of Cldns by Kashihara and Tanaka et al. (this manuscript), distinct from those described by Gonschior et al. (Nat Commun 2022) (*45*) and other groups.

STED, stimulated emission depletion microscopy.

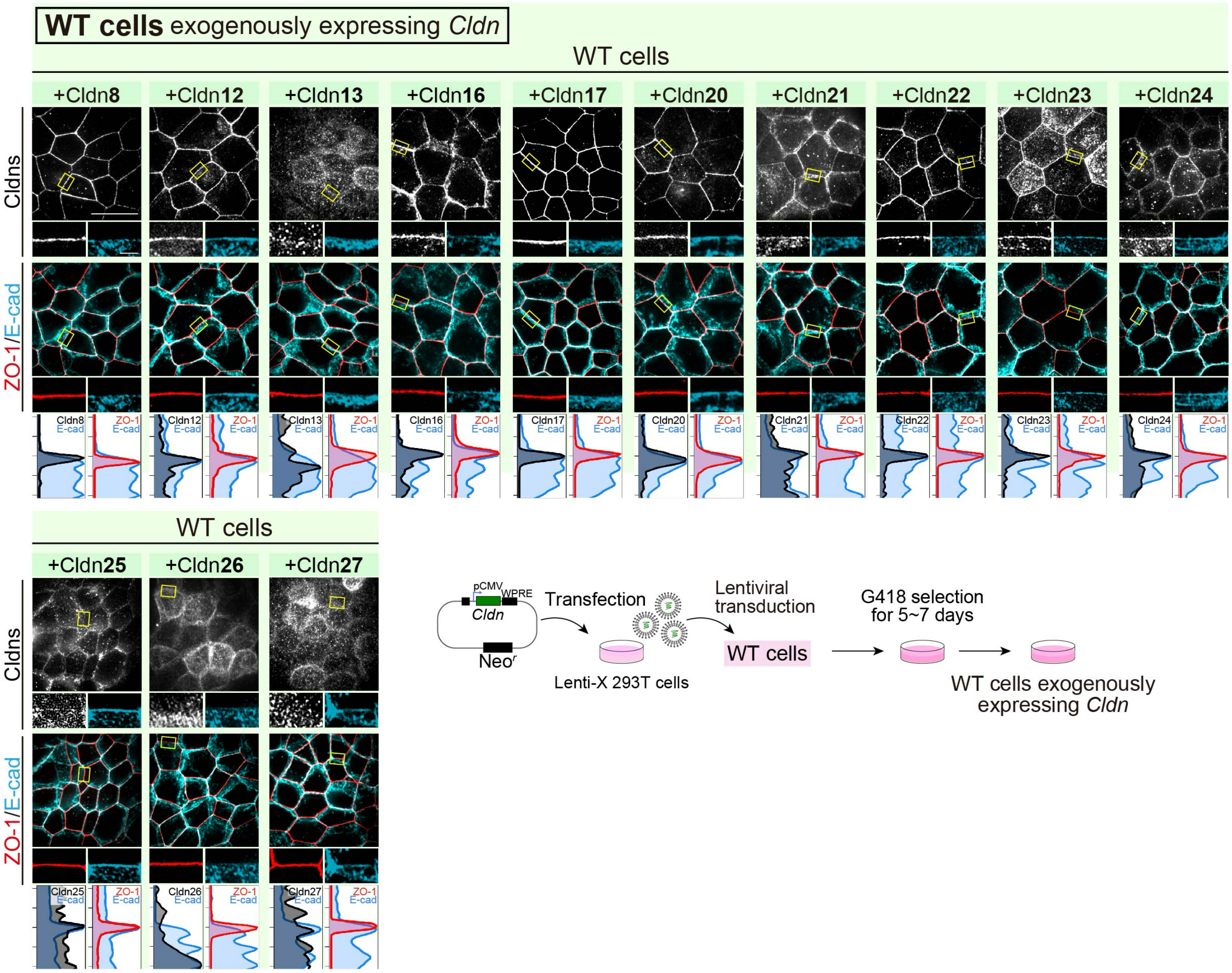

Fig. S10. Non-Autonomous TJ formation by Cldns in Class 3. Related to Fig. 4.

IF of WT cells exogenously expressing *Cldn8*, *12*, *13*, *16*, *17*, or *20–27* (Class 3), using the specific anti-Cldn antibody for the detection of Cldn8, and anti-Flag antibody for detection of other Cldns (Cldn12, 13, 16, 17, and 20–27). Co-staining of individual Cldns with ZO-1 and E-cad in their respective cells is presented. The bottom panels are magnified images of rectangular regions with fluorescence intensity plots. Scale bars: 10 μm (low magnification), 1 μm (high magnification).

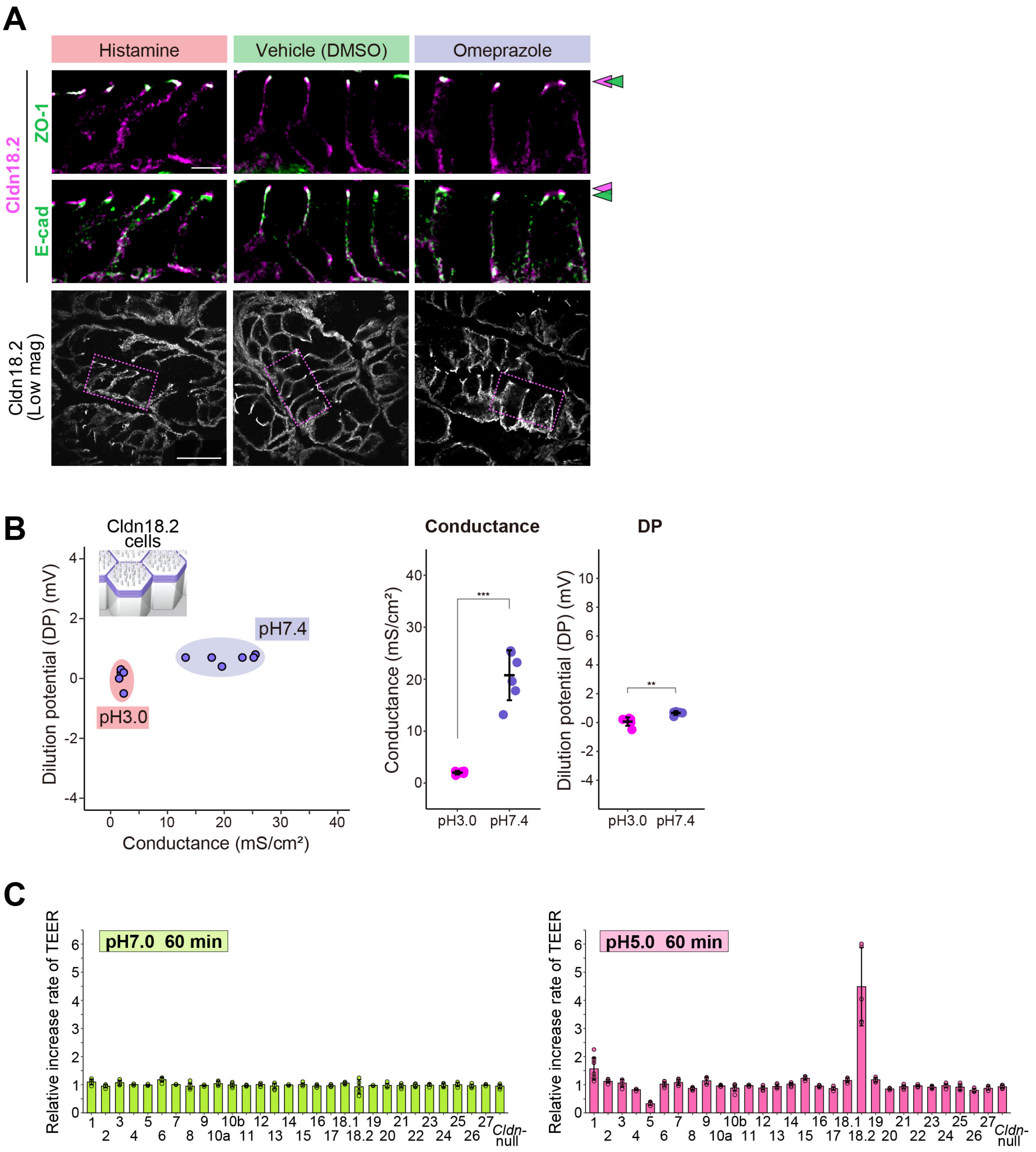

Fig. S11. An acid-enhancible TJ barrier in the stomach and Cldn18.2 cells. Related to Figs. 5 and 6.

(**A**) IF of stomachs from WT mice treated with histamine, omeprazole, or vehicle (DMSO) were co-stained by Cldn18.2 with ZO-1 or E-cad (E-cadherin). Top panels display magnified images of rectangular regions in bottom panels. Arrowheads indicate the concentrated signals of Cldn18.2 (magenta) and those of ZO-1 or E-cad (green). Scale bars: 20 µm (low magnification), 5 µm (high magnification).

(**B**) Trans-epithelial electrical conductance and DP of Cldn18.2 cells at pH 3.0 and 7.4 (n = 6 wells, respectively). Scatter plots (left) and dot plots (right) are shown. The same data were used for pH 3.0 in Fig. 6D. Values are means ± SDs. ***p < 0.001, ***p* < 0.01 (Welch's t-test for conductance, Mann-Whitney U test for DP).
(**C**) The relative increase rates of the TEER values 60 minutes after pH shifting from 7.0 to 7.0 (green, left) and from 7.0 to 5.0 (magenta, right) are shown (n = 5–9 wells of Cldn1–27 and Cldn-null cells, respectively).

Table S1.

Resources used in this manuscript are listed.

RESOURCE	SOURCE	IDENTIFIER
**Antibodies**		
Rabbit polyclonal anti-Claudin 1	Thermo Fisher Scientific	Cat# 51-9000<br>RRID: AB_2533916
Rabbit polyclonal anti-Claudin 2	Immuno-Biological Laboratories	Cat# 18825<br>RRID:AB_2341415
Rabbit polyclonal anti-Claudin 3	Thermo Fisher Scientific	Cat# 34-1700<br>RRID: AB_2533158
Rabbit polyclonal anti-Claudin 4	Thermo Fisher Scientific	Cat# 36-4800<br>RRID: AB_2533262
Mouse monoclonal anti-Claudin 4 (3E2C1)	Thermo Fisher Scientific	Cat# 32-9400<br>RRID: AB_2533096
Rabbit monoclonal anti-Claudin 5 (EPR7583)	Abcam	Cat# ab131259<br>RRID: AB_11157940
Rabbit polyclonal anti-Claudin 5/6	Morita et al., 1999. (*77*)	N/A
Rabbit polyclonal anti-Claudin 7	Immuno-Biological Laboratories	Cat# 18875<br>RRID: AB_494520
Rabbit polyclonal anti-Claudin 8	Immuno-Biological Laboratories	Cat# 18885<br>RRID: AB_529236
Goat polyclonal anti-Claudin 9	Santa Cruz Biotechnology	Cat# sc-17672<br>RRID: AB_2083126
Rabbit polyclonal anti-Claudin 10	Thermo Fisher Scientific	Cat# 38-8400<br>RRID: AB_2533386
Rabbit polyclonal anti-Claudin 11	Thermo Fisher Scientific	Cat# 36-4500<br>RRID: AB_2533259
Rabbit polyclonal anti-Claudin 12	Immuno-Biological Laboratories	Cat# 18801<br>RRID: AB_2341412
Goat polyclonal anti-Claudin 14	Everest Biotech	Cat# EB05529<br>RRID: AB_2079748
Rabbit polyclonal anti-Claudin 15	Immuno-Biological Laboratories	Cat# 18805<br>RRID:AB_2260813
Rabbit polyclonal anti-Claudin 16	Thermo Fisher Scientific	Ca# 34-5400<br>RRID: AB_2533174
Mouse monoclonal anti-Claudin 17 (410509)	R&D SYSTEMS	Ca# MAB4619
Rabbit monoclonal anti-Claudin 18 (34H14L15)	Thermo Fisher Scientific	Cat# 700178<br>RRID: AB_2532290
Rabbit polyclonal anti-Claudin 19	Miyamoto et al., 2005. (*20*)	N/A
Rabbit polyclonal anti-Claudin 23	This paper	N/A
Rat monoclonal anti-Claudin 25	This paper	N/A
Mouse monoclonal anti-ZO-1 (T8)	Itoh et al., 1993. (*78*)	N/A

Rabbit polyclonal anti-ZO-1	Thermo Fisher Scientific	Cat# 61-7300<br>RRID: AB_2533938
Rat monoclonal anti-E-cadherin (ECCD-2)	Shirayoshi et al., 1986. (*79*)	N/A
Rat monoclonal anti-Occludin (Moc37)	Saitou et al., 1997. (*80*)	N/A
Rabbit polyclonal anti-Tricellulin (N450)	Ikenouchi et al., 2005. (*81*)	N/A
Rabbit polyclonal anti-JAM cytoplasmic domain (C4)	Itoh et al., 2001. (*82*)	N/A
Rabbit polyclonal anti-α-catenin	Sigma-Aldrich	Cat# C2081<br>RRID: AB_476830
Mouse monoclonal anti-Vinculin (hVIN-1)	Sigma-Aldrich	Cat# V9131<br>RRID:AB_477629
Rabbit polyclonal anti-GAPDH	GeneTex	Cat# GTX100118<br>RRID: AB_1080976
Mouse monoclonal anti-DYKDDDDK tag (1E6)	FUJIFILM Wako Pure Chemical Corporation	Cat# 018-22381<br>RRID:AB_10659453
Donkey anti-Rabbit IgG (H+L), Alexa Fluor 488	Jackson ImmunoResearch	Cat# 711-545-152<br>RRID: AB_2313584
Donkey anti-Mouse IgG (H+L), Alexa Fluor 488	Thermo Fisher Scientific	Cat# A-21202<br>RRID: AB_141607
Donkey anti-Mouse IgG (H+L), Alexa Fluor 568	Thermo Fisher Scientific	Cat# A10037<br>RRID: AB_2534013
Donkey anti-Mouse IgG (H+L), Alexa Fluor 647	Thermo Fisher Scientific	Cat# A-31571<br>RRID: AB_162542
Donkey anti-Rat IgG (H+L), Cy3	Jackson ImmunoResearch	Cat# 712-165-153<br>RRID: AB_2340667
Donkey anti-Rat IgG (H+L), Alexa Fluor 647	Jackson ImmunoResearch	Cat# 712-605-153<br>RRID: AB_2340694
Donkey anti-Goat IgG (H+L), Alexa Fluor 488	Thermo Fisher Scientific	Cat# A-11055<br>RRID: AB_2534102
Donkey anti-Rabbit IgG, HRP	Cytiva	Cat# NA934<br>RRID: AB_772206
Sheep anti-Mouse IgG, HRP	Cytiva	Cat# NA931<br>RRID: AB_772210
Goat anti-Rat IgG, HRP	Cytiva	Cat# NA935<br>RRID:AB_772207
Mouse anti-Goat IgG, HRP	Santa Cruz Biotechnology	Cat# sc-2354<br>RRID:AB_628490
**Bacterial and virus strains**		
TaKaRa Competent Cells BL21	Takara Bio	Cat# 9126
**Chemicals, peptides, and recombinant proteins**		

Dulbecco's modified Eagle's medium (DMEM)	Shimadzu Diagnostics Corporation	Cat# 05919
Fetal bovine serum (FBS)	Thermo Fisher Scientific	Cat# 10270-106
T7 Endonuclease I reaction Mix	NIPPON GENE	Cat# 313-08801
ExoSAP-IT PCR Product Cleanup Reagent	Thermo Fisher Scientific	Cat# 78200.200.UL
Lipofectamine 3000	Thermo Fisher Scientific	Cat# L3000001
Puromycin dihydrochloride	Sigma-Aldrich	Cat# P8833<br>CAS: 58-58-2
Hygromycin B	FUJIFILM Wako Pure Chemical Corporation	Cat# 080-07683<br>CAS: 31282-04-9
Lentiviral High Titer Packaging Mix	Takara Bio	Cat# 6194
PEI MAX	Polysciences	Cat# 24765-1
Opti-MEM I Reduced Serum Medium	Thermo Fisher Scientific	Cat# 31985070
PEG 6000	FUJIFILM Wako Pure Chemical Corporation	Cat# 169-09125<br>CAS: 25322-68-3
G418 disulfate	Nacalai Tesque	Cat# 09380-44<br>CAS: 108321-42-2
PrimeSTAR Max DNA Polymerase	Takara Bio	Cat# R045A
ReverTra Ace qPCR RT Master Mix with gDNA Remover	TOYOBO	Cat# FSQ-301
PowerUP SYBR Green Master Mix for qPCR	Thermo Fisher Scientific	Cat# A25742
RNAlater Stabilization Solution	Thermo Fisher Scientific	Cat# AM7020
TRIzol Reagent	Thermo Fisher Scientific	Cat# 15596026
Ampicillin sodium salt	Nacalai Tesque	Cat# 19769-64<br>CAS: 69-52-3
Isopropyl 1-Thio-β-D-galactopyranoside (IPTG)	Nacalai Tesque	Cat# 19742-94<br>CAS: 367-93-1
Protease inhibitor cocktail	Nacalai Tesque	Cat# 03969-21
Glutathione Sepharose 4B	Cytiva	Cat# GE17-0756-01
Glutathione (Reduced Form)	FUJIFILM Wako Pure Chemical Corporation	Cat# 073-02013<br>CAS: 70-18-8
Coomassie Brilliant Blue (CBB) R-250	Nacalai Tesque	Cat# 09408-52<br>CAS: 6104-59-2
Bovine serum albumin (BSA)	Nacalai Tesque	Cat# 01860-36<br>CAS: 9048-46-8
Can Get Signal Solution 1 and 2	TOYOBO	Cat# NKB-101

Immobilon Western Chemiluminescent HRP Substrate	Merck	Cat# WBKLS0100
Can Get Signal immunostain Immunoreaction Enhancer Solution A	TOYOBO	Cat# NKB-501
ProLong Diamond Antifade Mountant	Thermo Fisher Scientific	Cat# P36961
Tissue-Tek O.C.T. Compound	Sakura Finetek Japan	Cat# 4583
EM 25% Glutaraldehyde	TAAB Laboratories Equipment	Cat# G004
Paraformaldehyde	FUJIFILM Wako Pure Chemical Corporation	Cat# 168-23255<br>CAS: 30525-89-4
Osmium Tetroxide (OsO4)	Heraeus South Africa (Pty)	Cat# 5027800<br>CAS: 20816-12-0
0.5% formvar solution	Nisshin EM	Cat# 604<br>CAS: 107-06-2
Platinum-carbon	JEOL	Cat# 781121370
Carbon	JEOL	Cat# 781121361
Photo-Flo 200 Solution	Eastman Kodak Company	Cat# 146 4510
Histamine Dihydrochloride	FUJIFILM Wako Pure Chemical Corporation	Cat# 081-03551<br>CAS: 56-92-8
Dimethyl sulfoxide (DMSO)	Nacalai Tesque	Cat# 08904-14<br>CAS: 67-68-5
Omeprazole	FUJIFILM Wako Pure Chemical Corporation	Cat# 158-03491<br>CAS: 73590-58-6
GST-Cldn1 C-term (188–211 aa)	This paper	N/A
GST-Cldn2 C-term (184–230 aa)	This paper	N/A
GST-Cldn3 C-term (186–219 aa)	This paper	N/A
GST-Cldn4 C-term (187–210 aa)	This paper	N/A
GST-Cldn5 C-term (187–218 aa)	This paper	N/A
GST-Cldn6 C-term (187–219 aa)	This paper	N/A
GST-Cldn7 C-term (188–211 aa)	This paper	N/A
GST-Cldn8 C-term (188–225 aa)	This paper	N/A
GST-Cldn9 C-term (187–217 aa)	This paper	N/A
GST-Cldn10 C-term (corresponding to 161–210 aa of Cldn10a, and 182–231 aa of Cldn10b)	This paper	N/A
GST-Cldn11 C-term (181–207 aa)	This paper	N/A
GST-Cldn12 C-term (198–244 aa)	This paper	N/A
GST-Cldn14 C-term (187–239 aa)	This paper	N/A
GST-Cldn15 C-term (185–227 aa)	This paper	N/A
GST-Cldn18 C-term (corresponding to 201–264 aa of Cldn18.1, and 201–264 aa of Cldn18.2)	This paper	N/A
GST-Cldn19 C-term (188–211 aa)	This paper	N/A

GST-Flag	This paper	N/A
**Critical commercial assays**		
Multiplex CRISPR/Cas9 Assembly Kit	Addgene deposited by Takashi Yamamoto. Sakuma et al., 2014. (*60*)	Cat# 1000000055
In-Fusion HD Cloning Kit	Takara Bio	Cat# 639650
RNeasy Mini Kit	QIAGEN N.V.	Cat# 74104
Pierce 660 nm Protein Assay Kit	Thermo Fisher Scientific	Cat# 22660
**Deposited data**		
Source data for RNA-seq	This paper	GSE274940
Source data for quantitative real-time PCR, immunofluorescence images, immunoblot images, electron microscopy images, Cryo-ET images, and physiological studies	This paper	Source data are available and prepared for deposition in Mendeley Data. For the initial submission, these data are accessible from the lead contact upon request.
**Experimental models: Cell lines**		
Mouse: EpH4 mouse mammary gland epithelial cells	Fialka et al., 1996. (*56*)	RRID: CVCL_0073
Mouse: *Cldn*-null cells	This paper	N/A
Mouse: Single *Cldn1*-expressing cells	This paper	N/A
Mouse: Single *Cldn2*-expressing cells	This paper	N/A
Mouse: Single *Flag-Cldn2*-expressing cells	This paper	N/A
Mouse: Single *Cldn3*-expressing cells	This paper	N/A
Mouse: Single *Flag-Cldn3*-expressing cells	This paper	N/A
Mouse: Single *EGFP-Cldn3*-expressing cells	This paper	N/A
Mouse: Single *Cldn4*-expressing cells	This paper	N/A
Mouse: Single *Cldn5*-expressing cells	This paper	N/A
Mouse: Single *Cldn6*-expressing cells	This paper	N/A
Mouse: Single *Cldn7*-expressing cells	This paper	N/A
Mouse: Single *Cldn8*-expressing cells	This paper	N/A
Mouse: Single *Cldn9*-expressing cells	This paper	N/A
Mouse: Single *Cldn10a*-expressing cells	This paper	N/A
Mouse: Single *Cldn10b*-expressing cells	This paper	N/A
Mouse: Single *Cldn11*-expressing cells	This paper	N/A
Mouse: Single *Cldn12*-expressing cells	This paper	N/A
Mouse: Single *Flag-Cldn12*-expressing cells	This paper	N/A
Mouse: Single *Flag-Cldn13*-expressing cells	This paper	N/A
Mouse: Single *Cldn14*-expressing cells	This paper	N/A
Mouse: Single *Cldn15*-expressing cells	This paper	N/A
Mouse: Single *Cldn16*-expressing cells	This paper	N/A

Mouse: Single *Flag-Cldn16*-expressing cells	This paper	N/A
Mouse: Single *Cldn17*-expressing cells	This paper	N/A
Mouse: Single *Flag-Cldn17*-expressing cells	This paper	N/A
Mouse: Single *Cldn18.1*-expressing cells	This paper	N/A
Mouse: Single *Cldn18.2*-expressing cells	This paper	N/A
Mouse: Single *Cldn19*-expressing cells	This paper	N/A
Mouse: Single *Flag-Cldn20*-expressing cells	This paper	N/A
Mouse: Single *Flag-Cldn21*-expressing cells	This paper	N/A
Mouse: Single *Flag-Cldn22*-expressing cells	This paper	N/A
Mouse: Single *Flag-Cldn23*-expressing cells	This paper	N/A
Mouse: Single *Flag-Cldn24*-expressing cells	This paper	N/A
Mouse: Single *Cldn25*-expressing cells	This paper	N/A
Mouse: Single *Flag-Cldn25*-expressing cells	This paper	N/A
Mouse: Single *Flag-Cldn26*-expressing cells	This paper	N/A
Mouse: Single *Flag-Cldn27*-expressing cells	This paper	N/A
Mouse: *ZO-1*/*ZO-2* double KO cells	Yano et al., 2021. (*59*)	N/A
Mouse: CSG120/7 mouse salivary gland carcinoma cells	Knowles & Franks., 1977. (*57*)	N/A
Mouse: MTD-1A mouse mammary gland epithelial cells	Hirano et al., 1987. (*58*)	RRID:CVCL_EG11
Human: Lenti-X 293T cells	Takara Bio	Cat# 632180
**Experimental models: Organisms/strains**		
Mouse: C57BL/6J mice	Japan SLC	
Mouse: *Cldn18.2*$^{-/-}$ (*Cldn18.2* KO) mice	Hayashi et al., 2012. (*22*)	N/A
**Oligonucleotides**		
Oligonucleotides for the guide RNA expression cassette targeting mouse *Cldn3*<br>Sense (5′→3′): CACCGCCTTCCCATGTGGCGCGTTT<br>Antisense (5′→3′): AAACAAACGCGCCACATGGGAAGG	This paper	N/A
Oligonucleotides for the guide RNA expression cassette targeting mouse *Cldn4* (*Cldn4#1*)<br>Sense (5′→3′): CACCGGGCCTCGCTTTACGTCGGCT<br>Antisense (5′→3′): AAACAGCCGACGTAAAGCGAGGCC	This paper	N/A
Oligonucleotides for the guide RNA expression cassette targeting mouse *Cldn4* (*Cldn4#2*)<br>Sense (5′→3′): CACCGAGGCTGAGCGATGGCGTCTA<br>Antisense (5′→3′): AAACTAGACGCCATCGCTCAGCCT	This paper	N/A
Oligonucleotides for the guide RNA expression cassette targeting mouse *Cldn7* (*Cldn7#1*)<br>Sense (5′→3′): CACCGGAGCTGCAAAATGTACGACT<br>Antisense (5′→3′): AAACAGTCGTACATTTTGCAGCTC	This paper	N/A
Oligonucleotides for the guide RNA expression cassette targeting mouse *Cldn7* (*Cldn7#2*)<br>Sense (5′→3′): CACCGGGATGGCAGTGCTCGCTATC<br>Antisense (5′→3′): AAACGATAGCGAGCACTGCCATCC	This paper	N/A

Oligonucleotides for the guide RNA expression cassette targeting mouse *Cldn8*<br>Sense (5′→3′): CACCGGTGGCCCTAAAGCGCGAGCT<br>Antisense (5′→3′): AAACAGCTCGCGCTTTAGGGCCAC	This paper	N/A
Oligonucleotides for the guide RNA expression cassette targeting mouse *Cldn9*<br>Sense (5′→3′): CACCGTGAACTCCTCGGCATGACCC<br>Antisense (5′→3′): AAACGGGTCATGCCGAGGAGTTCA	This paper	N/A
Oligonucleotides for the guide RNA expression cassette targeting mouse *Cldn12*<br>Sense (5′→3′): CACCGTGTGGTATTGCCTCTGTCGC<br>Antisense (5′→3′): AAACGCGACAGAGGCAATACCACA	This paper	N/A
Oligonucleotides for the guide RNA expression cassette targeting mouse *Cldn23*<br>Sense (5′→3′): CACCGACGCCGGTGGTGATGACGCT<br>Antisense (5′→3′): AAACAGCGTCATCACCACCGGCGTC	This paper	N/A
Oligonucleotides for the guide RNA expression cassette targeting mouse *Cldn25* (*Cldn25#1*)<br>Sense (5′→3′): CACCGTCTGATTTCCACCATCTACA<br>Antisense (5′→3′): AAACTGTAGATGGTGGAAATCAGA	This paper	N/A
Oligonucleotides for the guide RNA expression cassette targeting mouse *Cldn25* (*Cldn25#2*)<br>Sense (5′→3′): CACCGATACCAGAAGTCCGTGCCTA<br>Antisense (5′→3′): AAACTAGGCACGGACTTCTGGTATC	This paper	N/A
Quantitative real-time PCR primers for mouse *Cldn1*<br>Forward (5′→3′): CTGGAAGATGATGAGGTGCAGAAGA<br>Reverse (5′→3′): CCACTAATGTCGCCAGACCTGAA	Suzuki et al., 2019. (*62*)	N/A
Quantitative real-time PCR primers for mouse *Cldn2*<br>Forward (5′→3′): ATACTACCCTTTAGCCCTGACCGAGA<br>Reverse (5′→3′): CAGTAGGAGCACACATAACAGCTACCAC	Suzuki et al., 2019. (*62*)	N/A
Quantitative real-time PCR primers for mouse *Cldn3*<br>Forward (5′→3′): CACCACTACCAGCAGTCGATGAAC<br>Reverse (5′→3′): AGACTGTGTGTCGTCTGTCACCATC	Suzuki et al., 2019. (*62*)	N/A
Quantitative real-time PCR primers for mouse *Cldn4*<br>Forward (5′→3′): GGTAGCTCAGCTGTGACTTTGGACTC<br>Reverse (5′→3′): CTGGAGTAACGTGTAGGCTGAGTGAG	Suzuki et al., 2019. (*62*)	N/A
Quantitative real-time PCR primers for mouse *Cldn5*<br>Forward (5′→3′): TAACCTGAAAGGGCAGCTGGAGAAAC<br>Reverse (5′→3′): AGGTCCAGGCTAAGTCCTTTGGTTCAGTAG	Suzuki et al., 2019. (*62*)	N/A
Quantitative real-time PCR primers for mouse *Cldn6*<br>Forward (5′→3′): TGCCCACTCTATCATCCAGGACTTC<br>Reverse (5′→3′): AGGCCTGAGGCTGCCCAG	Suzuki et al., 2019. (*62*)	N/A
Quantitative real-time PCR primers for mouse *Cldn7*<br>Forward (5′→3′): ACGCCCATGAACGTTAAGTACGAG<br>Reverse (5′→3′): CTTTGCTTTCACTGCCTGGACA	Suzuki et al., 2019. (*62*)	N/A

Quantitative real-time PCR primers for mouse *Cldn8*<br>Forward (5′→3′): CTGGGGATAAAAGAGAAGGAGGCTGA<br>Reverse (5′→3′): AGGCTGCAAAGCAGGATAGCAGAAAG	Suzuki et al., 2019. (*62*)	N/A
Quantitative real-time PCR primers for mouse *Cldn9*<br>Forward (5′→3′): CTTGAGCTAACCCTCTGTAGTGGTTG<br>Reverse (5′→3′): CCAGAGTAAGAAAGTCCAGGAGAGCA	Suzuki et al., 2019. (*62*)	N/A
Quantitative real-time PCR primers for mouse *Cldn10a*<br>Forward (5′→3′): TCCCACACTTCAAGCCATGAGA<br>Reverse (5′→3′): GCAGACACTGGACAAAACTTCCAC	Suzuki et al., 2019. (*62*)	N/A
Quantitative real-time PCR primers for mouse *Cldn10b*<br>Forward (5′→3′): TGGGTGCTAGTGTCTTCCACACTG<br>Reverse (5′→3′): GAATCGGTAACGCAGATCTTCCAC	Suzuki et al., 2019. (*62*)	N/A
Quantitative real-time PCR primers for mouse *Cldn11*<br>Forward (5′→3′): CTGCCGAAAAATGGACGAACTG<br>Reverse (5′→3′): TGCACGTAGCCTGGAAGGATGA	Suzuki et al., 2019. (*62*)	N/A
Quantitative real-time PCR primers for mouse *Cldn12*<br>Forward (5′→3′): CAGACCAGTGTGTACTCAGACTTTCTACCC<br>Reverse (5′→3′): GAAGCAACATACTGACTGTCTCCTGACG	Suzuki et al., 2019. (*62*)	N/A
Quantitative real-time PCR primers for mouse *Cldn13*<br>Forward (5′→3′): GTCAACATCCCAGTATGCAGAGACTTTC<br>Reverse (5′→3′): GCTGGCCATCAAACATCTAAGGTATC	Suzuki et al., 2019. (*62*)	N/A
Quantitative real-time PCR primers for mouse *Cldn14*<br>Forward (5′→3′): GCAGCGTTGATAGCTGAAACTAGGTG<br>Reverse (5′→3′): CCAAGGCTGCTAGAAACTTTGCTG	Suzuki et al., 2019. (*62*)	N/A
Quantitative real-time PCR primers for mouse *Cldn15*<br>Forward (5′→3′): GCAGGGACCCTCCACATACTTG<br>Reverse (5′→3′): AGTTCATACTTGGTTCCAGCATACAGTG	Suzuki et al., 2019. (*62*)	N/A
Quantitative real-time PCR primers for mouse *Cldn16*<br>Forward (5′→3′): CTGGAGGTGAGCACTAAATGCAGAG<br>Reverse (5′→3′): AGTTACCACCAGCTTCAAGGGATGTTC	Suzuki et al., 2019. (*62*)	N/A
Quantitative real-time PCR primers for mouse *Cldn17*<br>Forward (5′→3′): CTTCCACCAGCTACGTCTAAGGCTTACTTC<br>Reverse (5′→3′): CTGAAGTACTCACAGTTTCTGGGGTGAC	Suzuki et al., 2019. (*62*)	N/A
Quantitative real-time PCR primers for mouse *Cldn18.1*<br>Forward (5′→3′): AGTATGAAGGGCTCTGGAGGAGTTG<br>Reverse (5′→3′): AGAACAATGCCCACGATCATCAG	Suzuki et al., 2019. (*62*)	N/A
Quantitative real-time PCR primers for mouse *Cldn18.2*<br>Forward (5′→3′): GTATTCAACTACCAAGGGCTATGGCGTTC<br>Reverse (5′→3′): ATCATCAGGGCTCGTACAGCTTGC	Suzuki et al., 2019. (*62*)	N/A
Quantitative real-time PCR primers for mouse *Cldn19*<br>Forward (5′→3′): CAGGTCTCTGTACTTTGACTGCTGTCTC<br>Reverse (5′→3′): CCAAATTCGTACCTGGCATTGAC	Suzuki et al., 2019. (*62*)	N/A
Quantitative real-time PCR primers for mouse *Cldn20*<br>Forward (5′→3′): GGTACACCAAGGAGATCATAGCGAAC<br>Reverse (5′→3′): ATGTACAGGGCTCCTCCAGGTTCATA	Suzuki et al., 2019. (*62*)	N/A

Quantitative real-time PCR primers for mouse *Cldn21*<br>Forward (5′→3′): CTGGGACTATTGGGACTTCTG<br>Reverse (5′→3′): AGGAGACTGGAAAGAGGGTAG	Suzuki et al., 2019. (*62*)	N/A
Quantitative real-time PCR primers for mouse *Cldn22*<br>Forward (5′→3′): CTCCCAGAACGTTCTAATGGGCTTAG<br>Reverse (5′→3′): AGTGCGGCAAGTAGTTTGTAAGGCAG	Suzuki et al., 2019. (*62*)	N/A
Quantitative real-time PCR primers for mouse *Cldn23*<br>Forward (5′→3′): TGGAGTCTGAGGGTGACTTGAACTTCTG<br>Reverse (5′→3′): AAGGAAGGCTTGACCTCCAGTTAGAGGAAG	Suzuki et al., 2019. (*62*)	N/A
Quantitative real-time PCR primers for mouse *Cldn24*<br>Forward (5′→3′): GATCATGGTTCATACCTAG<br>Reverse (5′→3′): TAAGGACACGACTCGGC	Suzuki et al., 2019. (*62*)	N/A
Quantitative real-time PCR primers for mouse *Cldn25*<br>Forward (5′→3′): ACGAGCAGTTCATGGAGAAG<br>Reverse (5′→3′): CAAAGCACATCAAGCCCAAG	Suzuki et al., 2019. (*62*)	N/A
Quantitative real-time PCR primers for mouse *Cldn26*<br>Forward (5′→3′): ATGAACCCTTTCTGGCAGG<br>Reverse (5′→3′): AACACCATCACGATCAGACTG	Suzuki et al., 2019. (*62*)	N/A
Quantitative real-time PCR primers for mouse *Cldn27*<br>Forward (5′→3′): ATCGTATGTGGTTGGGTCTG<br>Reverse (5′→3′): GGTGTGAGTAGCTGATGTAGATG	Suzuki et al., 2019. (*62*)	N/A
Quantitative real-time PCR primers for mouse *Gapdh*<br>Forward (5′→3′): AAGGTGGTGAAGCAGGCATCTGAG<br>Reverse (5′→3′): GGAAGAGTGGGAGTTGCTGTTGAAGTC	Suzuki et al., 2019. (*62*)	N/A
Quantitative real-time PCR primers for *Flag*<br>Forward (5′→3′): ATGGATTACAAGGATGACGAC<br>Reverse (5′→3′): CATCTCGAGTCCTCCCTTAT	This paper	N/A
**Recombinant DNA**		
pPGK-puro	Addgene deposited by Rudolf Jaenisch. Tucker et al., 1996. (*83*)	Cat# 11349<br>RRID:Addgene_11349
pTK-Hyg	Takara Bio	Cat# 631750
pLVSIN-CMV Neo	Takara Bio	Cat# 6181
pX330A	Addgene deposited by Takashi Yamamoto. Sakuma et al., 2014. (*60*)	Cat# 1000000055
pX330S	Addgene deposited by Takashi Yamamoto. Sakuma et al., 2014. (*60*)	Cat# 1000000055
pLVSIN Cldn1	This paper	N/A
pLVSIN Cldn2	This paper	N/A
pLVSIN Flag-Cldn2	This paper	N/A

pLVSIN Cldn3	This paper	N/A
pLVSIN Flag-Cldn3	This paper	N/A
pLVSIN EGFP-Cldn3	This paper	N/A
pLVSIN Cldn4	This paper	N/A
pLVSIN Cldn5	This paper	N/A
pLVSIN Cldn6	This paper	N/A
pLVSIN Cldn7	This paper	N/A
pLVSIN Cldn8	This paper	N/A
pLVSIN Cldn9	This paper	N/A
pLVSIN Cldn10a	This paper	N/A
pLVSIN Cldn10b	This paper	N/A
pLVSIN Cldn11	This paper	N/A
pLVSIN Cldn12	This paper	N/A
pLVSIN Flag-Cldn12	This paper	N/A
pLVSIN Flag-Cldn13	This paper	N/A
pLVSIN Cldn14	This paper	N/A
pLVSIN Cldn15	This paper	N/A
pLVSIN Cldn16	This paper	N/A
pLVSIN Flag-Cldn16	This paper	N/A
pLVSIN Cldn17	This paper	N/A
pLVSIN Flag-Cldn17	This paper	N/A
pLVSIN Cldn18.1	This paper	N/A
pLVSIN Cldn18.2	This paper	N/A
pLVSIN Cldn19	This paper	N/A
pLVSIN Flag-Cldn20	This paper	N/A
pLVSIN Flag-Cldn21	This paper	N/A
pLVSIN Flag-Cldn22	This paper	N/A
pLVSIN Flag-Cldn23	This paper	N/A
pLVSIN Flag-Cldn24	This paper	N/A
pLVSIN Cldn25	This paper	N/A
pLVSIN Flag-Cldn25	This paper	N/A
pLVSIN Flag-Cldn26	This paper	N/A
pLVSIN Flag-Cldn27	This paper	N/A
pGEX-6P-2	Merck	Cat# GE28-9546-50
pGEX-6P-2 Cldn1 C-term	This paper	N/A
pGEX-6P-2 Cldn2 C-term	This paper	N/A
pGEX-6P-2 Cldn3 C-term	This paper	N/A
pGEX-6P-2 Cldn4 C-term	This paper	N/A
pGEX-6P-2 Cldn5 C-term	This paper	N/A
pGEX-6P-2 Cldn6 C-term	This paper	N/A
pGEX-6P-2 Cldn7 C-term	This paper	N/A
pGEX-6P-2 Cldn8 C-term	This paper	N/A
pGEX-6P-2 Cldn9 C-term	This paper	N/A
pGEX-6P-2 Cldn10 C-term	This paper	N/A
pGEX-6P-2 Cldn11 C-term	This paper	N/A
pGEX-6P-2 Cldn12 C-term	This paper	N/A
pGEX-6P-2 Cldn14 C-term	This paper	N/A

pGEX-6P-2 Cldn15 C-term	This paper	N/A
pGEX-6P-2 Cldn18 C-term	This paper	N/A
pGEX-6P-2 Cldn19 C-term	This paper	N/A
pGEX-6P-2 Flag	This paper	N/A
**Software and algorithms**		
CRISPRdirect	Naito et al., 2015. (*63*)	https://crispr.dbcls.jp/
QuantStudio Design and Analysis Software version 1.5	Thermo Fisher Scientific	N/A
Illumina NovaSeq Control Software version 1.8	Illumina	N/A
Trimmomatic version 0.36	N/A	https://github.com/usadellab/Trimmomatic
FastQC version 0.11.9	N/A	https://www.bioinformatics.babraham.ac.uk/projects/fastqc/
STAR version 2.7.9a	N/A	https://github.com/alexdobin/STAR
RSEM version 1.3.3	N/A	https://github.com/deweylab/RSEM
edgeR version 3.42.4	N/A	https://bioconductor.org/packages/release/bioc/html/edgeR.html
limma version 3.56.2	N/A	https://bioconductor.org/packages/release/bioc/html/limma.html
Amersham Imager 680 Analysis Software version 2.0.0	Cytiva	N/A
MetaMorph version 7.1.0.1.161	Evident	N/A
cellSens Dimension Desktop 3.2 Software	Evident	N/A
JEOL SEM Center version 1.5	JEOL	N/A
JEOL TEM Center version 1.5.3	JEOL	N/A
MAPS	Thermo Fisher Scientific	https://www.thermofisher.com/jp/en/home/electron-microscopy/products/software-em-3d-vis/maps-software.html
AutoTEM	Thermo Fisher Scientific	https://www.thermofisher.com/jp/en/home/electron-microscopy/products/software-em-3d-vis/autotem-5-software.html
SerialEM	Mastronarde et al., 2005, (*68*) Schorb et al., 2019. (*69*)	https://bio3d.colorado.edu/SerialEM/

PACEtomo	Eisenstein et al., 2023. (*67*)	https://github.com/eisfabian/PACEtomo
IMOD	Kremer et al., 1996, (*70*) Mastronarde et al., 2008. (*71*)	https://bio3d.colorado.edu/imod/
Cryo-CARE	Buchholz et al., 2019. (*72*)	https://github.com/juglab/cryoCARE_pip
ImageJ version 1.53q	Schneider et al., 2012. (*84*)	https://imagej.nih.gov/ij/
R version 4.3.3	R Development Core Team	http://www.r-project.org
Excel	Microsoft	N/A
Blender version 3.6	N/A	https://www.blender.org/download/
**Other**		
mouse *Cldn1* gene	NCBI	Gene ID: 12737<br>RefSeq ID: NP_057883.1
mouse *Cldn2* gene	NCBI	Gene ID: 12738<br>RefSeq ID: NP_057884.1
mouse *Cldn3* gene	NCBI	Gene ID: 12739<br>RefSeq ID: NP_034032.1
mouse *Cldn4* gene	NCBI	Gene ID: 12740<br>RefSeq ID: NP_034033.1
mouse *Cldn5* gene	NCBI	Gene ID: 12741<br>RefSeq ID: NP_038833.2
mouse *Cldn6* gene	NCBI	Gene ID: 54419<br>RefSeq ID: NP_061247.1
mouse *Cldn7* gene	NCBI	Gene ID: 53624<br>RefSeq ID: NP_058583.1
mouse *Cldn8* gene	NCBI	Gene ID: 54420<br>RefSeq ID: NP_061248.1
mouse *Cldn9* gene	NCBI	Gene ID: 56863<br>RefSeq ID: NP_064689.2
mouse *Cldn10a* gene	NCBI	Gene ID: 58187<br>RefSeq ID: NP_001153568.1
mouse *Cldn10b* gene	NCBI	Gene ID: 58187<br>RefSeq ID: NP_067361.2
mouse *Cldn11* gene	NCBI	Gene ID: 18417<br>RefSeq ID: NP_032796.1
mouse *Cldn12* gene	NCBI	Gene ID: 64945<br>RefSeq ID: NP_075028.1
mouse *Cldn13* gene	NCBI	Gene ID: 57255<br>RefSeq ID: NP_065250.1
mouse *Cldn14* gene	NCBI	Gene ID: 56173<br>RefSeq ID: NP_062373.3

mouse *Cldn15* gene	NCBI	Gene ID: 60363<br>RefSeq ID: NP_068365.1
mouse *Cldn16* gene	NCBI	Gene ID: 114141<br>RefSeq ID: NP_444471.1
mouse *Cldn17* gene	NCBI	Gene ID: 239931<br>RefSeq ID: NP_852467.1
mouse *Cldn18.1* gene	NCBI	Gene ID: 56492<br>RefSeq ID: NP_062789.1
mouse *Cldn18.2* gene	NCBI	Gene ID: 56492<br>RefSeq ID: NP_001181850.1
mouse *Cldn19* gene	NCBI	Gene ID: 242653<br>RefSeq ID: NP_694745.1
mouse *Cldn20* gene	NCBI	Gene ID: 621628<br>RefSeq ID: NP_001095030.1
mouse *Cldn21* gene	NCBI	Gene ID: 100042785<br>RefSeq ID: NP_001371194.1
mouse *Cldn22* gene	NCBI	Gene ID: 75677<br>RefSeq ID: NP_083659.1
mouse *Cldn23* gene	NCBI	Gene ID: 71908<br>RefSeq ID: NP_082274.1
mouse *Cldn24* gene	NCBI	Gene ID: 100039801<br>RefSeq ID: NP_001104788.1
mouse *Cldn25* gene	NCBI	Gene ID: 224250<br>RefSeq ID: NP_741968.1
mouse *Cldn26* gene	NCBI	Gene ID: 74720<br>RefSeq ID: NP_083346.1
mouse *Cldn27* gene	NCBI	Gene ID: 546519<br>RefSeq ID: NP_001079004.1
Millex-HP 0.45 μm, PES 33 mm	Merck	Cat# SLHP033NK
QuantStudio 5 Real-Time PCR System (384-well block)	Thermo Fisher Scientific	Cat# A28140
Cryomill	Tokken	Cat# SK-100
Semi-permeable polyethylene terephthalate (PET) membrane filter, ThinCert Cell Culture Inserts for 24 Well Plates	Greiner Bio-One	Cat# 662641
Semi-permeable polyethylene terephthalate (PET) membrane filter, ThinCert Cell Culture Inserts for 12 Well Plates	Greiner Bio-One	Cat# 665641
Sonifier 250	Branson	N/A
Image scanner	Seiko Epson Corporation	Cat# GT-X970
Immobilon-P membrane, PVDF, 0.45 μm	Merck	Cat# IPVH00010<br>CAS: 24937-79-9
Chemiluminescence imager	Cytiva	Cat# Amersham Imager 680
Spinning disk super-resolution confocal microscope	Evident	Cat# SpinSR10

Cryostat	Leica Microsystems	Cat# CM1850
Ultra microtome	Leica Microsystems	Cat# Leica EM UC6
Electron microscope for thin-section electron microscopy	JEOL	Cat# JEM-1400Flash<br>Cat# JSM-7900F
Electron microscope for freeze-fracture electron microscopy	JEOL	Cat# JEM-1400Plus
Nitrogen slusher machine	JEOL	Cat# EM-19510SNPD
Freeze-fracture equipment	JEOL	Cat# EM19500-NFSDT
Millicell ERS-2 Voltohmmeter	Merck	Cat# MERS00002
Amplifier	NIHON KOHDEN CORPORATION	Cat# CEZ-9100
Silicon wafer strip	Canosis	Cat# SiD-2
Filter paper	ADVANTEC	Cat# 4-903-10
pH meter	HORIBA	Cat# F-71
Titanium grid	Ted Pella	Cat# 1GT100
Gold grid	Nisshin EM	Cat# 2612-1
Vitrobot Mark IV	Thermo Fisher Scientific	N/A
Aquilos 2 dual-beam instrument	Thermo Fisher Scientific	N/A
Krios G4 transmission electron microscope	Thermo Fisher Scientific	N/A

Movie S1. Cryo-electron tomography of WT and *Cldn*-null cells –Part 1.
Movie S2. Cryo-electron tomography of WT and *Cldn*-null cells –Part 2.
Related to Fig. 1 and Fig. S3. Cryo-electron tomography (Cryo-ET) of WT and *Cldn*-null cells. 3D reconstructions from the most apical region of the lateral membranes are shown as false-colored images. These reconstructions highlight the intercellular regions of TJs (magenta) and lipid bilayers (cyan). Scale bars: 50 nm (low magnification), 10 nm (high magnification).

Movie S3. Cryo-electron tomography of single *Cldn2*- and *Cldn3*-expressing cells –Part 1.
Movie S4. Cryo-electron tomography of single *Cldn2*- and *Cldn3*-expressing cells –Part 2.
Related to Fig. 2 and Fig. S3. Cryo-electron tomography (Cryo-ET) of single *Cldn2*- and *Cldn3*-expressing cells (Cldn2 cells and Cldn3 cells, respectively). 3D reconstructions from the most apical region of the lateral membranes are shown as false-colored images. These reconstructions highlight the intercellular regions of TJs (magenta) and lipid bilayers (cyan). Scale bars: 50 nm (low magnification), 10 nm (high magnification).

Movie S5. Classification of 27 Cldn family members.
Related to Fig. 4. 3D plot depicting trans-epithelial electrical conductance, dilution potential, and TJ strand number of single *Cldn*-expressing cells. The 27 Cldns are classified into four main classes (Class1–4) with subclasses.

REFERENCES AND NOTES

1. S. Tsukita, M. Furuse, M. Itoh, Multifunctional strands in tight junctions. *Nat. Rev. Mol. Cell Biol.* **2**, 285–293 (2001).

2. S. Tsukita, H. Tanaka, A. Tamura, The claudins: From tight junctions to biological systems. *Trends Biochem. Sci.* **44**, 141–152 (2019).

3. L. Meoli, D. Günzel, The role of claudins in homeostasis. *Nat. Rev. Nephrol.* **19**, 587–603 (2023).

4. J. M. Anderson, C. M. Van Itallie, Physiology and function of the tight junction. *Cold Spring Harb. Perspect. Biol.* **1**, a002584 (2009).

5. C. T. Capaldo, A. Nusrat, Claudin switching: Physiological plasticity of the tight junction. *Semin. Cell Dev. Biol.* **42**, 22–29 (2015).

6. M. A. Odenwald, J. R. Turner, The intestinal epithelial barrier: A therapeutic target? *Nat. Rev. Gastroenterol. Hepatol.* **14**, 9–21 (2017).

7. J. Piontek, S. M. Krug, J. Protze, G. Krause, M. Fromm, Molecular architecture and assembly of the tight junction backbone. *Biochim. Biophys. Acta Biomembr.* **1862**, 183279 (2020).

8. S. Citi, M. Fromm, M. Furuse, L. González-Mariscal, A. Nusrat, S. Tsukita, J. R. Turner, A short guide to the tight junction. *J. Cell Sci.* **137**, jcs261776 (2024).

9. M. Furuse, M. Hata, K. Furuse, Y. Yoshida, A. Haratake, Y. Sugitani, T. Noda, A. Kubo, S. Tsukita, Claudin-based tight junctions are crucial for the mammalian epidermal barrier. *J. Cell Biol.* **156**, 1099–1111 (2002).

10. T. Nitta, M. Hata, S. Gotoh, Y. Seo, H. Sasaki, N. Hashimoto, M. Furuse, S. Tsukita, Size-selective loosening of the blood-brain barrier in claudin-5-deficient mice. *J. Cell Biol.* **161**, 653–660 (2003).

11. S. Mazaud-Guittot, E. Meugnier, S. Pesenti, X. Wu, H. Vidal, A. Gow, B. Le Magueresse-Battistoni, Claudin 11 deficiency in mice results in loss of the sertoli cell epithelial phenotype in the testis1. *Biol. Reprod.* **82**, 202–213 (2010).

12. R. Tokumasu, K. Yamaga, Y. Yamazaki, H. Murota, K. Suzuki, A. Tamura, K. Bando, Y. Furuta, I. Katayama, S. Tsukita, Dose-dependent role of claudin-1 in vivo in orchestrating features of atopic dermatitis. *Proc. Natl. Acad. Sci. U.S.A.* **113,** E4061–E4068 (2016).

13. S. Citi, Intestinal barriers protect against disease. *Science* **359**, 1097–1098 (2018).

14. C. Greene, N. Hanley, M. Campbell, Claudin-5: Gatekeeper of neurological function. *Fluids Barriers CNS* **16**, 3 (2019).

15. H. Suzuki, T. Nishizawa, K. Tani, Y. Yamazaki, A. Tamura, R. Ishitani, N. Dohmae, S. Tsukita, O. Nureki, Y. Fujiyoshi, Crystal structure of a claudin provides insight into the architecture of tight junctions. *Science* **344**, 304–307 (2014).

16. H. Suzuki, K. Tani, A. Tamura, S. Tsukita, Y. Fujiyoshi, Model for the architecture of claudin-based paracellular ion channels through tight junctions. *J. Mol. Biol.* **427**, 291–297 (2015).

17. Y. Saitoh, H. Suzuki, K. Tani, K. Nishikawa, K. Irie, Y. Ogura, A. Tamura, S. Tsukita, Y. Fujiyoshi, Structural insight into tight junction disassembly by *Clostridium perfringens* enterotoxin. *Science* **347**, 775–778 (2015).

18. S. Nakamura, K. Irie, H. Tanaka, K. Nishikawa, H. Suzuki, Y. Saitoh, A. Tamura, S. Tsukita, Y. Fujiyoshi, Morphologic determinant of tight junctions revealed by claudin-3 structures. *Nat. Commun.* **10**, 816 (2019).

19. K. Mineta, Y. Yamamoto, Y. Yamazaki, H. Tanaka, Y. Tada, K. Saito, A. Tamura, M. Igarashi, T. Endo, K. Takeuchi, S. Tsukita, Predicted expansion of the claudin multigene family. *FEBS Lett.* **585**, 606–612 (2011).

20. T. Miyamoto, K. Morita, D. Takemoto, K. Takeuchi, Y. Kitano, T. Miyakawa, K. Nakayama, Y. Okamura, H. Sasaki, Y. Miyachi, M. Furuse, S. Tsukita, Tight junctions in Schwann cells of

peripheral myelinated axons: A lesson from claudin-19-deficient mice. *J. Cell Biol.* **169**, 527–538 (2005).

21. S. Milatz, S. M. Krug, R. Rosenthal, D. Günzel, D. Müller, J.-D. Schulzke, S. Amasheh, M. Fromm, Claudin-3 acts as a sealing component of the tight junction for ions of either charge and uncharged solutes. *Biochim. Biophys. Acta* **1798**, 2048–2057 (2010).

22. D. Hayashi, A. Tamura, H. Tanaka, Y. Yamazaki, S. Watanabe, K. Suzuki, K. Suzuki, K. Sentani, W. Yasui, H. Rakugi, Y. Isaka, S. Tsukita, Deficiency of claudin-18 causes paracellular H+ leakage, up-regulation of interleukin-1β, and atrophic gastritis in mice. *Gastroenterology* **142**, 292–304 (2012).

23. H. Tanaka, M. Takechi, H. Kiyonari, G. Shioi, A. Tamura, S. Tsukita, Intestinal deletion of Claudin-7 enhances paracellular organic solute flux and initiates colonic inflammation in mice. *Gut* **64**, 1529–1538 (2015).

24. H. Tanaka, M. Imasato, Y. Yamazaki, K. Matsumoto, K. Kunimoto, J. Delpierre, K. Meyer, M. Zerial, N. Kitamura, M. Watanabe, A. Tamura, S. Tsukita, Claudin-3 regulates bile canalicular paracellular barrier and cholesterol gallstone core formation in mice. *J. Hepatol.* **69**, 1308–1316 (2018).

25. S. Amasheh, N. Meiri, A. H. Gitter, T. Schöneberg, J. Mankertz, J. D. Schulzke, M. Fromm, Claudin-2 expression induces cation-selective channels in tight junctions of epithelial cells. *J. Cell Sci.* **115**, 4969–4976 (2002).

26. C. M. Van Itallie, S. Rogan, A. Yu, L. S. Vidal, J. Holmes, J. M. Anderson, Two splice variants of claudin-10 in the kidney create paracellular pores with different ion selectivities. *Am. J. Physiol. Renal Physiol.* **291**, F1288–F1299 (2006).

27. A. S. L. Yu, M. H. Cheng, S. Angelow, D. Günzel, S. A. Kanzawa, E. E. Schneeberger, M. Fromm, R. D. Coalson, Molecular basis for cation selectivity in claudin-2-based paracellular pores: Identification of an electrostatic interaction site. *J. Gen. Physiol.* **133**, 111–127 (2009).

28. C. R. Weber, G. H. Liang, Y. Wang, S. Das, L. Shen, A. S. L. Yu, D. J. Nelson, J. R. Turner, Claudin-2-dependent paracellular channels are dynamically gated. *eLife* **4**, e09906 (2015).

29. H. Tanaka, Y. Yamamoto, H. Kashihara, Y. Yamazaki, K. Tani, Y. Fujiyoshi, K. Mineta, K. Takeuchi, A. Tamura, S. Tsukita, Claudin-21 has a paracellular channel role at tight junctions. *Mol. Cell. Biol.* **36**, 954–964 (2016).

30. R. Rosenthal, D. Günzel, J. Piontek, S. M. Krug, C. Ayala-Torres, C. Hempel, D. Theune, M. Fromm, Claudin-15 forms a water channel through the tight junction with distinct function compared to claudin-2. *Acta Physiol.* **228**, e13334 (2020).

31. A. Tamura, H. Hayashi, M. Imasato, Y. Yamazaki, A. Hagiwara, M. Wada, T. Noda, M. Watanabe, Y. Suzuki, S. Tsukita, Loss of claudin-15, but not claudin-2, causes Na+ deficiency and glucose malabsorption in mouse small intestine. *Gastroenterology* **140**, 913–923 (2011).

32. M. Wada, A. Tamura, N. Takahashi, S. Tsukita, Loss of claudins 2 and 15 from mice causes defects in paracellular Na^+ flow and nutrient transport in gut and leads to death from malnutrition. *Gastroenterology* **144**, 369–380 (2013).

33. K. Matsumoto, M. Imasato, Y. Yamazaki, H. Tanaka, M. Watanabe, H. Eguchi, H. Nagano, H. Hikita, T. Tatsumi, T. Takehara, A. Tamura, S. Tsukita, Claudin 2 deficiency reduces bile flow and increases susceptibility to cholesterol gallstone disease in mice. *Gastroenterology* **147**, 1134–1145.e10 (2014).

34. P.-Y. Tsai, B. Zhang, W.-Q. He, J.-M. Zha, M. A. Odenwald, G. Singh, A. Tamura, L. Shen, A. Sailer, S. Yeruva, W.-T. Kuo, Y.-X. Fu, S. Tsukita, J. R. Turner, IL-22 upregulates epithelial claudin-2 to drive diarrhea and enteric pathogen clearance. *Cell Host Microbe* **21**, 671–681.e4 (2017).

35. J. N. Curry, M. Saurette, M. Askari, L. Pei, M. B. Filla, M. R. Beggs, P. S. Rowe, T. Fields, A. J. Sommer, C. Tanikawa, Y. Kamatani, A. P. Evan, M. Totonchi, R. T. Alexander, K. Matsuda, A. S. Yu, Claudin-2 deficiency associates with hypercalciuria in mice and human kidney stone disease. *J. Clin. Invest.* **130**, 1948–1960 (2020).

36. T. Otani, T. P. Nguyen, S. Tokuda, K. Sugihara, T. Sugawara, K. Furuse, T. Miura, K. Ebnet, M. Furuse, Claudins and JAM-A coordinately regulate tight junction formation and epithelial polarity. *J. Cell Biol.* **218**, 3372–3396 (2019).

37. K. Reinhard, B. Rengstl, P. Oehm, K. Michel, A. Billmeier, N. Hayduk, O. Klein, K. Kuna, Y. Ouchan, S. Wöll, E. Christ, D. Weber, M. Suchan, T. Bukur, M. Birtel, V. Jahndel, K. Mroz, K. Hobohm, L. Kranz, M. Diken, K. Kühlcke, Ö. Türeci, U. Sahin, An RNA vaccine drives expansion and efficacy of claudin-CAR-T cells against solid tumors. *Science* **367**, 446–453 (2020).

38. N. Roehlen, A. Saviano, H. El Saghire, E. Crouchet, Z. Nehme, F. Del Zompo, F. Jühling, M. A. Oudot, S. C. Durand, F. H. T. Duong, S. Cherradi, V. Gonzalez Motos, N. Almeida, C. Ponsolles, L. Heydmann, T. Ostyn, A. Lallement, P. Pessaux, E. Felli, A. Cavalli, J. Sgrignani, C. Thumann, O. Koutsopoulos, B. C. Fuchs, Y. Hoshida, M. Hofmann, M. Vyberg, B. M. Viuff, E. D. Galsgaard, G. Elson, A. Toso, M. Meyer, R. Iacone, T. Schweighoffer, G. Teixeira, S. Moll, C. De Vito, T. Roskams, I. Davidson, D. Heide, M. Heikenwälder, M. B. Zeisel, J. Lupberger, L. Mailly, C. Schuster, T. F. Baumert, A monoclonal antibody targeting nonjunctional claudin-1 inhibits fibrosis in patient-derived models by modulating cell plasticity. *Sci. Transl. Med.* **14**, eabj4221 (2022).

39. I. Nakayama, C. Qi, Y. Chen, Y. Nakamura, L. Shen, K. Shitara, Claudin 18.2 as a novel therapeutic target. *Nat. Rev. Clin. Oncol.* **21**, 354–369 (2024).

40. C. R. Stadler, U. Ellinghaus, L. Fischer, H. Bähr-Mahmud, M. Rao, C. Lindemann, A. Chaturvedi, C. Scharf, I. Biermann, B. Hebich, A. Malz, G. Beresin, G. Falck, A. Häcker, A. Houben, M. Erdeljan, K. Wolf, M. Kullmann, P. Chang, Ö. Türeci, U. Şahin, Preclinical efficacy and pharmacokinetics of an RNA-encoded T cell-engaging bispecific antibody targeting human claudin 6. *Sci. Transl. Med.* **16**, eadl2720 (2024).

41. Y. Okamura, S. Tsukita, Morphology of freeze-substituted myelinated axon in mouse peripheral nerves. *Brain Res.* **383**, 146–158 (1986).

42. K. Umeda, J. Ikenouchi, S. Katahira-Tayama, K. Furuse, H. Sasaki, M. Nakayama, T. Matsui, S. Tsukita, M. Furuse, S. Tsukita, ZO-1 and ZO-2 independently determine where claudins are polymerized in tight-junction strand formation. *Cell* **126**, 741–754 (2006).

43. A. S. L. Yu, M. H. Cheng, R. D. Coalson, Calcium inhibits paracellular sodium conductance through claudin-2 by competitive binding. *J. Biol. Chem.* **285**, 37060–37069 (2010).

44. K. Sato, I. Matsumoto, K. Suzuki, A. Tamura, A. Shiraishi, H. Kiyonari, J. Kasamatsu, H. Yamamoto, T. Miyasaka, D. Tanno, A. Miyahara, T. Zong, T. Kagesawa, A. Oniyama, K. Kawamura, Y. Kitai, A. Umeki, E. Kanno, H. Tanno, K. Ishii, S. Tsukita, K. Kawakami, Deficiency of lung-specific claudin-18 leads to aggravated infection with Cryptococcus deneoformans through dysregulation of the microenvironment in lungs. *Sci. Rep.* **11**, 21110 (2021).

45. H. Gonschior, C. Schmied, R. E. Van der Veen, J. Eichhorst, N. Himmerkus, J. Piontek, D. Günzel, M. Bleich, M. Furuse, V. Haucke, M. Lehmann, Nanoscale segregation of channel and barrier claudins enables paracellular ion flux. *Nat. Commun.* **13**, 4985 (2022).

46. B. Zhou, P. Flodby, J. Luo, D. R. Castillo, Y. Liu, F.-X. Yu, A. McConnell, B. Varghese, G. Li, N.-O. Chimge, M. Sunohara, M. N. Koss, W. Elatre, P. Conti, J. M. Liebler, C. Yang, C. N. Marconett, I. A. Laird-Offringa, P. Minoo, K. Guan, B. R. Stripp, E. D. Crandall, Z. Borok, Claudin-18-mediated YAP activity regulates lung stem and progenitor cell homeostasis and tumorigenesis. *J. Clin. Invest.* **128**, 970–984 (2018).

47. N. Shashikanth, M. M. France, R. Xiao, X. Haest, H. E. Rizzo, J. Yeste, J. Reiner, J. R. Turner, Tight junction channel regulation by interclaudin interference. *Nat. Commun.* **13**, 3780 (2022).

48. J. Hou, A. Renigunta, M. Konrad, A. S. Gomes, E. E. Schneeberger, D. L. Paul, S. Waldegger, D. A. Goodenough, Claudin-16 and claudin-19 interact and form a cation-selective tight junction complex. *J. Clin. Invest.* **118**, 619–628 (2008).

49. Y. Gong, V. Renigunta, N. Himmerkus, J. Zhang, A. Renigunta, M. Bleich, J. Hou, Claudin-14 regulates renal Ca++ transport in response to CaSR signalling via a novel microRNA pathway. *EMBO J.* **31**, 1999–2012 (2012).

50. J. Hou, A. Renigunta, J. Yang, S. Waldegger, Claudin-4 forms paracellular chloride channel in the kidney and requires claudin-8 for tight junction localization. *Proc. Natl. Acad. Sci. U.S.A.* **107**, 18010–18015 (2010).

51. S. M. Krug, D. Günzel, M. P. Conrad, R. Rosenthal, A. Fromm, S. Amasheh, J. D. Schulzke, M. Fromm, Claudin-17 forms tight junction channels with distinct anion selectivity. *Cell. Mol. Life Sci.* **69**, 2765–2778 (2012).

52. M. R. Beggs, K. Young, W. Pan, D. D. O'Neill, M. Saurette, A. Plain, J. Rievaj, M. R. Doschak, E. Cordat, H. Dimke, R. T. Alexander, Claudin-2 and claudin-12 form independent, complementary pores required to maintain calcium homeostasis. *Proc. Natl. Acad. Sci. U.S.A.* **118**, e2111247118 (2021).

53. A. Raya-Sandino, K. M. Lozada-Soto, N. Rajagopal, V. Garcia-Hernandez, A.-C. Luissint, J. C. Brazil, G. Cui, M. Koval, C. A. Parkos, S. Nangia, A. Nusrat, Claudin-23 reshapes epithelial tight junction architecture to regulate barrier function. *Nat. Commun.* **14**, 6214 (2023).

54. Y. Hashimoto, C. Besmond, N. Boddaert, A. Munnich, M. Campbell, A loss of function mutation in CLDN25 causing Pelizaeus-Merzbacher-like leukodystrophy. *Hum. Mol. Genet.* **33**, 1055–1063 (2024).

55. D.-W. Wang, W.-H. Zhang, G. Danil, K. Yang, J.-K. Hu, The role and mechanism of claudins in cancer. *Front. Oncol.* **12**, 1051497 (2022).

56. I. Fialka, H. Schwarz, E. Reichmann, M. Oft, M. Busslinger, H. Beug, The estrogen-dependent c-JunER protein causes a reversible loss of mammary epithelial cell polarity involving a destabilization of adherens junctions. *J. Cell Biol.* **132**, 1115–1132 (1996).

57. M. A. Knowles, L. M. Franks, Stages in neoplastic transformation of adult epithelial cells by 7,12-dimethylbenz(a)anthracene in vitro. *Cancer Res.* **37**, 3917–3924 (1977).

58. S. Hirano, A. Nose, K. Hatta, A. Kawakami, M. Takeichi, Calcium-dependent cell-cell adhesion molecules (cadherins): Subclass specificities and possible involvement of actin bundles. *J. Cell Biol.* **105**, 2501–2510 (1987).

59. T. Yano, K. Tsukita, H. Kanoh, S. Nakayama, H. Kashihara, T. Mizuno, H. Tanaka, T. Matsui, Y. Goto, A. Komatsubara, K. Aoki, R. Takahashi, A. Tamura, S. Tsukita, A microtubule-LUZP1 association around tight junction promotes epithelial cell apical constriction. *EMBO J.* **40**, e104712 (2021).

60. T. Sakuma, A. Nishikawa, S. Kume, K. Chayama, T. Yamamoto, Multiplex genome engineering in human cells using all-in-one CRISPR/Cas9 vector system. *Sci. Rep.* **4**, 5400 (2014).

61. D. Y. Guschin, A. J. Waite, G. E. Katibah, J. C. Miller, M. C. Holmes, E. J. Rebar, A rapid and general assay for monitoring endogenous gene modification. *Methods Mol. Biol.* **649**, 247–256 (2010).

62. K. Suzuki, K. Sentani, H. Tanaka, T. Yano, K. Suzuki, M. Oshima, W. Yasui, A. Tamura, S. Tsukita, Deficiency of stomach-type claudin-18 in mice induces gastric tumor formation independent of H pylori infection. *Cell. Mol. Gastroenterol. Hepatol.* **8**, 119–142 (2019).

63. Y. Naito, K. Hino, H. Bono, K. Ui-Tei, CRISPRdirect: Software for designing CRISPR/Cas guide RNA with reduced off-target sites. *Bioinformatics* **31**, 1120–1123 (2015).

64. A. Rigort, F. J. B. Bäuerlein, E. Villa, M. Eibauer, T. Laugks, W. Baumeister, J. M. Plitzko, Focused ion beam micromachining of eukaryotic cells for cryoelectron tomography. *Proc. Natl. Acad. Sci. U.S.A.* **109**, 4449–4454 (2012).

65. G. Wolff, R. W. A. L. Limpens, S. Zheng, E. J. Snijder, D. A. Agard, A. J. Koster, M. Bárcena, Mind the gap: Micro-expansion joints drastically decrease the bending of FIB-milled cryo-lamellae. *J. Struct. Biol.* **208**, 107389 (2019).

66. W. J. H. Hagen, W. Wan, J. A. G. Briggs, Implementation of a cryo-electron tomography tilt-scheme optimized for high resolution subtomogram averaging. *J. Struct. Biol.* **197**, 191–198 (2017).

67. F. Eisenstein, H. Yanagisawa, H. Kashihara, M. Kikkawa, S. Tsukita, R. Danev, Parallel cryo electron tomography on in situ lamellae. *Nat. Methods* **20**, 131–138 (2023).

68. D. N. Mastronarde, Automated electron microscope tomography using robust prediction of specimen movements. *J. Struct. Biol.* **152**, 36–51 (2005).

69. M. Schorb, I. Haberbosch, W. J. H. Hagen, Y. Schwab, D. N. Mastronarde, Software tools for automated transmission electron microscopy. *Nat. Methods* **16**, 471–477 (2019).

70. J. R. Kremer, D. N. Mastronarde, J. R. McIntosh, Computer visualization of three-dimensional image data using IMOD. *J. Struct. Biol.* **116**, 71–76 (1996).

71. D. N. Mastronarde, Correction for non-perpendicularity of beam and tilt axis in tomographic reconstructions with the IMOD package. *J. Microsc.* **230**, 212–217 (2008).

72. T.-O. Buchholz, M. Jordan, G. Pigino, F. Jug, "Cryo-CARE: Content-Aware Image Restoration for cryo-transmission electron microscopy data," in *2019 IEEE 16th International Symposium on Biomedical Imaging (ISBI 2019)* (IEEE, 2019), pp. 502–506.

73. J. Jumper, R. Evans, A. Pritzel, T. Green, M. Figurnov, O. Ronneberger, K. Tunyasuvunakool, R. Bates, A. Žídek, A. Potapenko, A. Bridgland, C. Meyer, S. A. A. Kohl, A. J. Ballard, A. Cowie, B. Romera-Paredes, S. Nikolov, R. Jain, J. Adler, T. Back, S. Petersen, D. Reiman, E. Clancy, M. Zielinski, M. Steinegger, M. Pacholska, T. Berghammer, S. Bodenstein, D. Silver, O. Vinyals, A. W. Senior, K. Kavukcuoglu, P. Kohli, D. Hassabis, Highly accurate protein structure prediction with AlphaFold. *Nature* **596**, 583–589 (2021).

74. N. A. Baker, D. Sept, S. Joseph, M. J. Holst, J. A. McCammon, Electrostatics of nanosystems: Application to microtubules and the ribosome. *Proc. Natl. Acad. Sci. U.S.A.* **98**, 10037–10041 (2001).

75. M. Ester, H.-P. Kriegel, J. Sander, X. Xu, "A density-based algorithm for discovering clusters in large spatial databases with noise," in *Proceedings of the Second International Conference on Knowledge Discovery and Data Mining* (AAAI Press, 1996), pp. 226–231.

76. M. Hahsler, M. Piekenbrock, D. Doran, dbscan: Fast density-based clustering with R. *J. Stat. Softw.* **91**, 1–30 (2019).

77. K. Morita, H. Sasaki, M. Furuse, S. Tsukita, Endothelial claudin: Claudin-5/TMVCF constitutes tight junction strands in endothelial cells. *J. Cell Biol.* **147**, 185–194 (1999).

78. M. Itoh, A. Nagafuchi, S. Yonemura, T. Kitani-Yasuda, S. Tsukita, S. Tsukita, The 220-kD protein colocalizing with cadherins in nonepithelial cells is identical to ZO-1, a tight junction-associated protein in epithelial cells: cDNA cloning and immunoelectron microscopy. *J. Cell Biol.* **121**, 491–502 (1993).

79. Y. Shirayoshi, K. Hatta, M. Hosoda, S. Tsunasawa, F. Sakiyama, M. Takeichi, Cadherin cell adhesion molecules with distinct binding specificities share a common structure. *EMBO J.* **5**, 2485–2488 (1986).

80. M. Saitou, Y. Ando-Akatsuka, M. Itoh, M. Furuse, J. Inazawa, K. Fujimoto, S. Tsukita, Mammalian occludin in epithelial cells: Its expression and subcellular distribution. *Eur. J. Cell Biol.* **73**, 222–231 (1997).

81. J. Ikenouchi, M. Furuse, K. Furuse, H. Sasaki, S. Tsukita, S. Tsukita, Tricellulin constitutes a novel barrier at tricellular contacts of epithelial cells. *J. Cell Biol.* **171**, 939–945 (2005).

82. M. Itoh, H. Sasaki, M. Furuse, H. Ozaki, T. Kita, S. Tsukita, Junctional adhesion molecule (JAM) binds to PAR-3: A possible mechanism for the recruitment of PAR-3 to tight junctions. *J. Cell Biol.* **154**, 491–498 (2001).

83. K. L. Tucker, C. Beard, J. Dausmann, L. Jackson-Grusby, P. W. Laird, H. Lei, E. Li, R. Jaenisch, Germ-line passage is required for establishment of methylation and expression patterns of imprinted but not of nonimprinted genes. *Genes Dev.* **10**, 1008–1020 (1996).

84. C. A. Schneider, W. S. Rasband, K. W. Eliceiri, NIH Image to ImageJ: 25 years of image analysis. *Nat. Methods* **9**, 671–675 (2012).
